# Methylxanthines: The Major Impact of Caffeine in Clinical Practice in Patients Diagnosed with Apnea of Prematurity

**DOI:** 10.3390/jcm14238417

**Published:** 2025-11-27

**Authors:** Adela-Valeria Neamțu, Ovidiu Mircea Zlatian, Costel-Valentin Manda, Ramona Cioboată, Carla-Maria Bărbulescu-Mateescu, Cătălina Coteanu, Luminița-Cristina Chiuțu, Liliana Stanca, Olivia Garofița Mateescu, Simona-Daniela Neamțu

**Affiliations:** 1 Doctoral School, University of Medicine and Pharmacy of Craiova, 200349 Craiova, Romania; nav29.adela@yahoo.com; 2 Department of Microbiology, Faculty of Medicine, University of Medicine and Pharmacy of Craiova, 200638 Craiova, Romania; 3 Medical Laboratory, Clinical Emergency County Hospital, 200642 Craiova, Romania; 4 Department of Chemistry, University of Medicine and Pharmacy of Craiova, 200349 Craiova, Romania; valentin.manda@umfcv.ro; 5 Clinic of Pneumology, “Victor Babes” Infectious Diseases Hospital, 200515 Craiova, Romania; ramona_cioboata@yahoo.com; 6 Department of Pneumology, Faculty of Medicine, University of Medicine and Pharmacy of Craiova, 200638 Craiova, Romania; 7 Faculty of Medicine, University of Medicine and Pharmacy of Craiova, 200638 Craiova, Romania; adasavon@yahoo.com; 8 Medical Laboratory, “Filantropia” Clinical Municipal Hospital, 200143 Craiova, Romania; coteanuc29@yahoo.com (C.C.); simona.neamtu@umfcv.ro (S.-D.N.); 9 Clinic of Anesthesia and Intensive Care, Clinical Emergency County Hospital, 200642 Craiova, Romania; luminita.chiutu@umfcv.ro; 10 Department of Anesthesia and Intensive Care, University of Medicine and Pharmacy of Craiova, 200349 Craiova, Romania; 11 Department of Forensic Medicine, University of Medicine and Pharmacy of Craiova, 200349 Craiova, Romania; liliana.stanca@umfcv.ro; 12 Laboratory of Pathology, “Filantropia” Clinical Municipal Hospital, 200143 Craiova, Romania; garofita.mateescu@umfcv.ro; 13 Department of Histology, University of Medicine and Pharmacy of Craiova, 200349 Craiova, Romania; 14 Department of Immunology, Hematology, University of Medicine and Pharmacy of Craiova, 200349 Craiova, Romania

**Keywords:** apnea of prematurity, caffeine citrate, theophylline, aminophylline, bronchopulmonary dysplasia, neonatal pharmacology

## Abstract

**Background:** Apnea of prematurity affects at least 85% of infants born before 34 weeks’ gestation and represents a significant clinical challenge in neonatal intensive care. Methylxanthines, including caffeine, theophylline, and aminophylline, have emerged as the primary pharmacological intervention for this condition. **Objective:** To conduct a comprehensive systematic review of the use of methylxanthine in the treatment and prevention of apnea episodes in preterm infants, evaluating efficacy, safety, and long-term outcomes. **Methods:** We searched multiple databases including PubMed, Embase, Web of Science for randomized controlled trials, retrospective studies, or case–control studies of methylxanthine effects in preterm apnea. Risk of bias was assessed using the Cochrane Risk of Bias tool. Results were summarized narratively and grouped by methylxanthine type, study design, and primary outcomes (reduction in frequency and severity of apnea episodes, success of extubation, risk of bronchopulmonary dysplasia). **Results:** Twenty-five studies (*n* = 4599 preterm infants) were included. The landmark Caffeine for Apnea of Prematurity (CAP) trial (*n* = 2006) demonstrated that caffeine therapy significantly reduced bronchopulmonary dysplasia (36.3% vs. 46.9%, adjusted OR 0.63) and facilitated the earlier discontinuation of positive airway pressure (median 1 week earlier). Studies with a smaller number of cases have consistently demonstrated the efficacy of methylxanthines in reducing the incidence of bronchopulmonary dysplasia and apneic episodes and in supporting successful extubation. Long-term follow-up at 11 years showed improved pulmonary function (FEV_1_ z-score −1.00 vs. −1.53). **Discussion:** Limitations of this review include heterogeneity in outcome definitions, small sample sizes in early studies, and the dominance of evidence from the CAP trial. Methylxanthines, particularly caffeine, are an evidence-based intervention used for apnea of prematurity, with demonstrated benefits that extend beyond reducing the frequency and severity of apnea episodes, including decreasing the risk of bronchopulmonary dysplasia as well as reducing the need for mechanical ventilation. No external funding was received for this review. No registration record exists for this systematic review.

## 1. Introduction

Apnea of prematurity (AOP) affects up to 85% of infants born before 34 weeks of gestation and represents a significant clinical challenge in neonatal intensive care [1]. Characterized by pauses in breathing with associated bradycardia or oxygen desaturation, AOP poses risks for hypoxemia, prolonged ventilation, and adverse neurodevelopmental outcomes. The pathophysiology of apnea of prematurity is multifactorial, involving immature respiratory control mechanisms, unstable respiratory drive, and inadequate responses to hypoxia and hypercapnia [2,3,4,5] (Detailed descriptions of apnea pathophysiology are provided in Supplementary Materials).

**Figure 1:**
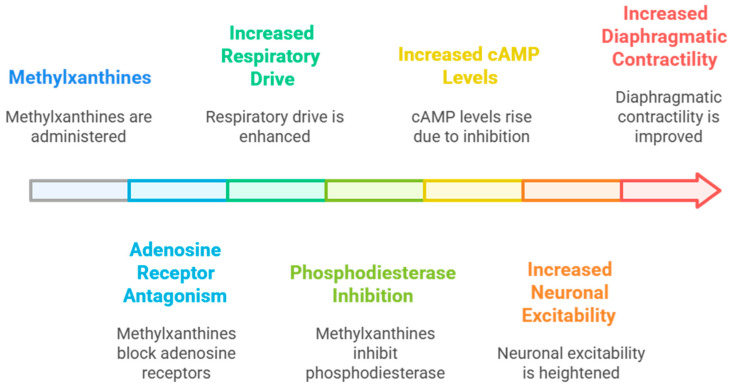
Mechanisms of action of methylxanthines in apnea of prematurity.

Methylxanthines (caffeine citrate, theophylline, and aminophylline) have emerged as the cornerstone of pharmacologic management. Acting as adenosine receptor antagonists, these agents stimulate central respiratory drive and improve diaphragmatic contractility (Figure 1) [6]. Caffeine citrate has become the preferred therapy owing to its longer half-life, wider therapeutic window, and superior safety profile compared to theophylline and aminophylline [7].

Since the landmark Caffeine for Apnea of Prematurity (CAP) trial [8], methylxanthine therapy has been widely implemented. However, there is variability in the timing of treatment, dosage, and reported outcomes of the studies. Furthermore, new data have emerged regarding long-term effects on neurological and pulmonary development. This warrants an updated synthesis of the evidence base.

Detailed descriptions of developmental respiratory control, methylxanthine pharmacology, effects of methylxantines on the physiology of newborns and historical developments are provided in Supplementary Materials [9,10,11,12,13,14,15,16,17,18,19,20,21,22,23,24,25,26,27,28,29,30,31,32,33,34,35]. 

Despite widespread clinical use, the relative efficacy and safety of different methylxanthines, optimal dosing strategies, and long-term outcomes remain incompletely synthesized across the full span of available evidence. Therefore, this systematic review aims to critically evaluate current evidence regarding the efficacy, safety, and long-term outcomes of methylxanthine use in preterm infants with apnea of prematurity.

## 2. Research Question and Objectives

The review aimed to answer the following research question, formulated using the PICO framework:

- Population (P): Preterm infants (<37 weeks of gestation) diagnosed with apnea of prematurity (AOP).
- Intervention (I): Any methylxanthine therapy (caffeine, theophylline, aminophylline), administered by any route or regimen.
- Comparator (C): Placebo, no treatment, or another methylxanthine compound.
- Outcomes (O): Primary—reduction in apnea frequency or severity. Secondary—extubation success, duration of mechanical ventilation, bronchopulmonary dysplasia, mortality, and adverse events.

Primary objectives: To systematically evaluate the efficacy, safety, short- and long-term outcomes of methylxanthine therapy in the management of apnea of prematurity [36].

Secondary objectives included comparing the relative efficacy and safety profiles of different methylxanthine compounds (caffeine, theophylline, aminophylline), identifying optimal dosing strategies and treatment protocols for methylxanthine therapy, assessing the economic implications and healthcare utilization impacts of methylxanthine treatment, evaluating the quality of existing evidence, and identifying gaps in current research. The LC-MS method developed at the Research Center of the Faculty of Pharmacy in Craiova can be used to accurately and precisely quantify methylxanthines in human plasma and has proven useful in determining the actual plasma levels of caffeine and theophylline in patients diagnosed with apnea of prematurity. The methylxanthine titers obtained in the Research Center of the Faculty of Pharmacy in Craiova were correlated with hematological parameters provided by the Mindray BC-6800Plus analyzer, with immunological parameters provided by the Maglumi X3 analyzer, with biochemical parameters provided by the Mindray BS-1000M analyzer and the clinical status of the patients within the Filantropia Craiova Municipal Clinical Hospital [37].

## 3. Methods

This systematic review was designed and reported in accordance with the Preferred Reporting Items for Systematic Reviews and Meta-Analyses (PRISMA) 2020 guidelines to ensure methodological rigor and transparent reporting. (Supplementary Table S3) The PRISMA 2020 guidelines represent the most current international standard for systematic review reporting and include enhanced guidance for risk of bias assessment, certainty of evidence evaluation, and handling of complex interventions [38]. No protocol was registered for this review.

### 3.1. Eligibility Criteria

Eligibility criteria were defined a priori according to study characteristics, population, intervention, and outcomes.

Inclusion criteria:

- Population: Preterm infants (<37 weeks’ gestation) or with low birth weight (<2500 g) experiencing apnea of prematurity.
- Intervention: Caffeine, theophylline, or aminophylline used for prophylaxis or treatment of AOP, via any route (intravenous, oral, nasogastric).
- Comparator: Placebo, no treatment, or other methylxanthine compounds.
- Study design: Randomized controlled trials (RCTs), prospective or retrospective cohort studies, and case–control studies.
- Outcomes: At least one clinically relevant respiratory or safety outcome reported.

Exclusion criteria:

- Studies involving term infants (≥37 weeks’ gestation) or apnea due to other causes (e.g., sepsis, seizures, metabolic disorders).
- Studies limited to pharmacokinetic data without clinical outcomes.
- Non-methylxanthine interventions only.
- Case reports, reviews, editorials, or conference abstracts without extractable data.
- Studies from before 1970.

### 3.2. Search Strategy

Comprehensive searches were conducted across multiple electronic databases to ensure maximum coverage of relevant literature. We searched the databases PubMed, Embase, Web of Science for studies published between 1970 and 2025. The last search in databases was performed on 31 August 2025.

The search strategy was developed using a combination of Medical Subject Headings (MeSH) terms and free-text keywords (Supplementary Table S1), with input from a medical librarian experienced in systematic review methodology. The search strategy was designed to be highly sensitive to capture all potentially relevant studies. Primary search terms included terms relevant for the population (e.g., “preterm infant” OR “premature infant” OR “low birth weight” OR “very low birth weight” OR “extremely low birth weight”), condition: “apnea of prematurity” OR “apnoea of prematurity” OR “neonatal apnea” OR “infant apnea”, intervention (e.g., “methylxanthine” OR “caffeine” OR “theophylline” OR “aminophylline” OR “xanthine derivative”), study design (e.g., “randomized controlled trial” OR “controlled clinical trial” OR “randomised” OR “placebo”).

No language restrictions were applied initially, with non-English studies translated when necessary. We searched studies published between 1970–2025 (methylxanthines first used for apnea of prematurity in late 1970s); both published and unpublished studies were included to minimize publication bias.

### 3.3. Study Selection Process

The study selection process followed a two-stage screening approach and data extraction conducted by two independent reviewers, with disagreements resolved through discussion or consultation with a third reviewer.

All retrieved records were imported into Mendeley (Elsevier) for deduplication. All retrieved citations underwent initial screening based on title and abstract review. Studies were excluded at this stage only if they clearly did not meet the inclusion criteria (e.g., adult populations, non-methylxanthine interventions, non-randomized designs). Studies passing initial screening underwent full-text review for final inclusion decisions. Reasons for exclusion were documented for all studies excluded at this stage. Reference lists of all included studies and relevant systematic reviews were manually screened to identify additional potentially eligible studies not captured by the electronic searches.

After study selection, included studies were categorized according to the intervention type (caffeine, theophylline, aminophylline), study design (RCT, cohort, case–control), and clinical outcomes (efficacy, respiratory support, safety, long-term outcomes). Eligibility for each synthesis group was verified by comparing the intervention and outcome definitions against the pre-specified inclusion criteria. Only studies meeting these criteria for each domain were included in the corresponding synthesis subsection.

### 3.4. Data Extraction

Data extraction was performed independently by two reviewers in alignment with PRISMA 2020 and Cochrane methodological guidance to ensure consistency and transparency in data collection. Extracted information included:

- Study characteristics (authors, year, country, design, sample size);
- Population demographics (gestational age, birth weight);
- Intervention details (methylxanthine type, dose, route, duration);
- Comparator characteristics;
- Primary and secondary outcomes (efficacy, safety, respiratory support);
- Risk of bias elements.

After extraction, all entries were compared for accuracy and completeness. Discrepancies were resolved through discussion and, when necessary, adjudication by a third reviewer. Any data inconsistencies or missing details were verified against the original article texts, figures, and Supplementary Materials. Extracted data were tabulated in Microsoft Excel for structured comparison across studies.

When numerical data were unavailable in tabular form, information was obtained from figures, text descriptions, or Supplementary Appendices. If key details such as standard deviations or effect estimates were not reported, the data were qualitatively summarized without imputation. No attempts were made to contact original study authors for missing data, given the historical span of included studies (1970–2025) and the focus on published evidence.

Quality control was maintained through double-checking of all extracted entries and consistent use of standardized terminology for outcomes and dosing parameters.

## 4. Results

### 4.1. Study Selection and Characteristics

**Figure 2:**
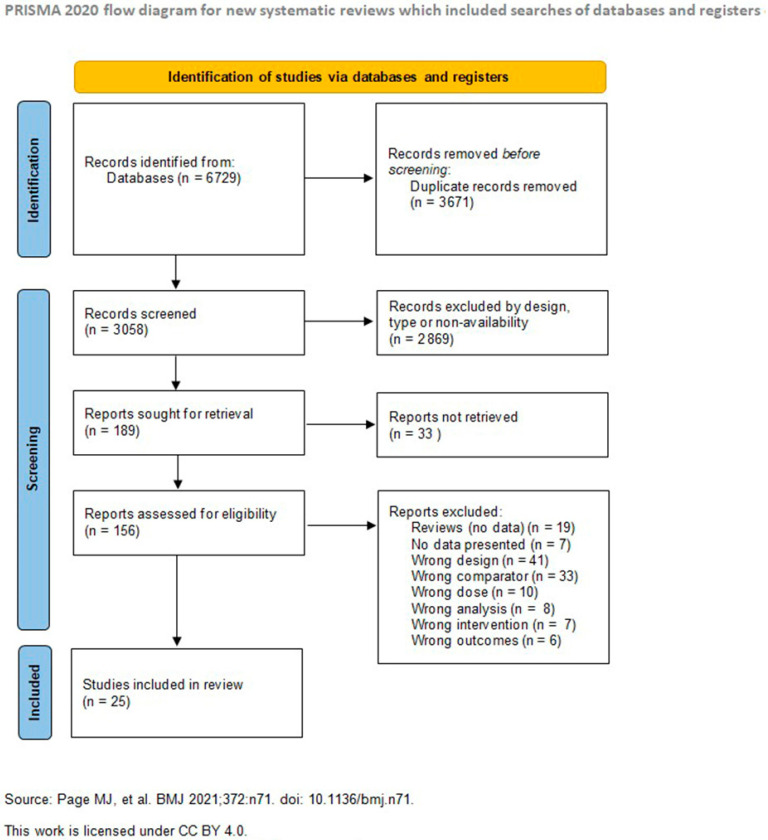
PRISMA 2020 flow diagram of study selection [38]. To view a copy of this license, visit https://creativecommons.org/licenses/by/4.0/ (access date 20 November 2025)

The systematic search strategy identified 6729 potentially relevant citations across all databases. After removal of duplicates, 3058 unique citations underwent title and abstract screening. Two reviewers independently screened titles, abstracts, and full texts for eligibility, resolving discrepancies by consensus or third-party adjudication. Study characteristics were then extracted and summarized in tabular form. The studies written in other languages were first translated to English. We excluded narrative reviews, systematic reviews, meta-analyses, case reports, editorials, commentaries, and letters to the editor, keeping only experimental studies in form of original experimental investigations, including randomized controlled trials, prospective and retrospective cohort studies, short communications, and conference abstracts. We ended up with 189 studies that were checked for eligibility by abstract retrieval and analysis by the two reviewers. From these, 33 could not be retrieved. We excluded further 131 studies for wrong study type and design, comparator, dose, intervention, analysis or outcome proceeded to full-text review, with 25 studies meeting final inclusion criteria. The study selection process is detailed in the PRISMA flow diagram (Figure 2).

Flow of information through the systematic review process, illustrating the number of records identified, screened, assessed for eligibility, and included in the final synthesis. From 6729 records initially retrieved across databases, 3058 unique studies were screened after duplicate removal. Following title and abstract screening and full-text assessment, 25 studies met the inclusion criteria and were included in the qualitative synthesis.

The 25 eligible studies encompassed 4599 preterm infants with individual study sizes ranging from 18 to 2006 participants across a range of gestational ages and clinical settings [8,34,39,40,41,42,43,44,45,46,47,48,49,50,51,52,53,54,55,56,57,58,59,60,61]. The landmark CAP trial [8] contributed ~40% of all participants, highlighting the relative paucity of large-scale trials in this field. Many trials enrolled very preterm infants born at <32 weeks’ gestation [34,41,43,44,46,47,48,50,51,52,54], though several included moderately preterm populations up to 34–36 weeks [55,57,61] or defined [42,50,51,52,58,59] eligibility based on birth weight (<1250 g or <1500 g) [42,43,44,45,46,53]. The majority were randomized controlled trials evaluating caffeine citrate versus placebo or no treatment [43,44,46,53,54,56,60,61], while others compared methylxanthines head-to-head, including caffeine versus theophylline or aminophylline [42,50,51,52,58,59]. A smaller number of early studies investigated theophylline monotherapy [34,40,41], and one multicenter trial examined multiple caffeine dosing regimens [56]. Sample sizes varied widely, from fewer than 20 infants in early single-center studies [40,41] to more than 2000 participants in the landmark CAP trial by Schmidt et al. (2006) [8]. Most studies were conducted in neonatal intensive care units across North America, Europe, Asia, and Australia, reflecting broad international representation. Although early investigations were limited by small sample sizes and incomplete blinding, later multicenter trials demonstrated robust methodology and standardized outcome reporting. All included studies reported at least one neonatal or respiratory outcome, while long-term neurodevelopmental data were available only from the CAP trial and its follow-up by Doyle et al. (2017) [49].

### 4.2. Risk of Bias Assessment

Risk of bias assessment was conducted using the revised Cochrane Risk of Bias tool (RoB 2) for randomized trials [62]. This tool evaluates bias across five domains: randomization process, deviations from intended interventions, missing outcome data, measurement of the outcome, selection of the reported result.

**Figure 3:**
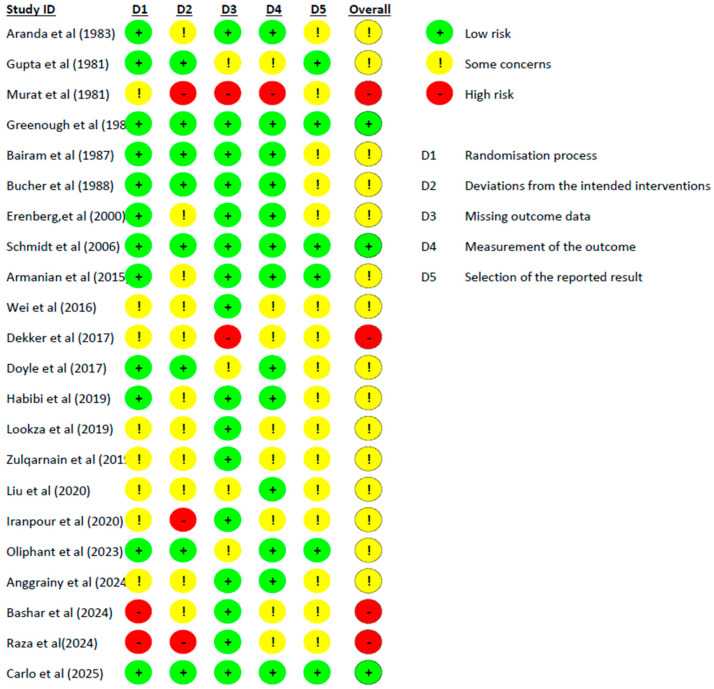
Risk of bias assessment for the included RCTs [8,34,39,40,41,42,43,44,46,47,48,49,50,51,52,53,54,56,57,58,59,60,61].

Each domain was rated as “Low risk,” “Some concerns,” or “High risk” of bias. An overall risk of bias judgment was made for each study based on the domain-specific assessments (Figure 3 and Supplementary Table S2). Two reviewers independently assessed risk of bias, with disagreements resolved through discussion.

### 4.3. Synthesis of Results

To ensure transparency and comparability across included studies, all extracted outcome measures were systematically synthesized according to PRISMA 2020 guidance. The principal outcome domains included efficacy in reducing apnea episodes, effects on respiratory support and extubation success, safety and adverse effects, long-term outcomes such as bronchopulmonary dysplasia and neurodevelopment, and comparative effectiveness between methylxanthine compounds. Each primary and secondary endpoint was summarized using the effect measures originally reported by the included trials, including odds ratios (ORs), relative risks (RRs), mean or median differences, and proportions. Because of methodological and clinical heterogeneity across trials, a meta-analysis was not conducted. Instead, results were organized by outcome domain to facilitate interpretation and comparison. A narrative synthesis was performed, with emphasis on the consistency and magnitude of effects across studies.

The synthesized outcome measures for all primary clinical endpoints—including apnea reduction, bronchopulmonary dysplasia, duration of mechanical ventilation, extubation success, intermittent hypoxemia, neurodevelopmental outcomes, and adverse effects—are presented in Table 1.

### 4.4. Certainty of Evidence, Heterogeneity, and Sensitivity Analysis

#### 4.4.1. Certainty of the Body of Evidence

The certainty (confidence) of the evidence for each outcome was assessed qualitatively, following the principles outlined in the Grading of Recommendations Assessment, Development, and Evaluation (GRADE) approach [66]. Certainty was judged based on study design, risk of bias, consistency of findings across studies, directness of evidence, and precision of effect estimates. Randomized controlled trials were initially considered high-certainty evidence, whereas observational studies were considered low-certainty, with possible upgrading or downgrading depending on these domains.

The evidence base for methylxanthine therapy in apnea of prematurity is dominated by the CAP trial, which provides high-quality evidence for caffeine efficacy and safety [8]. The remaining studies, while smaller and of variable quality, provide consistent supporting evidence for methylxanthine efficacy across different compounds and populations. The consistency of findings across studies, despite variations in methodology and populations, strengthens confidence in the overall conclusions regarding methylxanthine efficacy and safety for apnea of prematurity treatment [67,68].

In this review, overall certainty was summarized descriptively rather than numerically, as no pooled quantitative estimates were calculated. Evidence confidence was interpreted as high when results were consistent across multiple RCTs with low risk of bias (e.g., caffeine efficacy outcomes), moderate when findings were generally consistent but limited by sample size or study quality (e.g., extubation success, long-term neurodevelopment), and low when evidence was derived primarily from small or heterogeneous studies (e.g., minor adverse events or growth outcomes).

These qualitative judgments were used to inform the discussion of evidence strength and limitations for each outcome domain in the synthesis of results.

#### 4.4.2. Exploration of Heterogeneity

Differences among study results were explored qualitatively to identify potential sources of heterogeneity. Variability in effect estimates across studies was primarily attributed to differences in methylxanthine type, dosage, timing of initiation, and study population characteristics. Studies administering caffeine within the first 72 h of life generally demonstrated greater efficacy in reducing apnea frequency and duration of respiratory support compared to those initiating treatment later.

Heterogeneity was also observed between randomized controlled trials and observational studies, with RCTs typically reporting stronger and more consistent treatment effects. Variation in outcome definitions—particularly for apnea events and extubation success—further contributed to differences in reported efficacy. Theophylline- and aminophylline-based studies showed higher rates of adverse effects, which may reflect both pharmacologic properties and differences in dosing protocols.

No formal statistical heterogeneity measures (e.g., I^2^ or meta-regression) were applied because quantitative pooling was not performed. Nonetheless, qualitative exploration indicated that differences in gestational age, treatment timing, and study design were the main sources of heterogeneity across the included evidence base.

#### 4.4.3. Sensitivity Analysis

Sensitivity analyses were conducted qualitatively to evaluate the robustness of the synthesized results. The direction and strength of observed effects were compared across subsets of studies differing in methodological quality, publication date, and sample size.

Excluding early studies conducted before 1990, which generally had smaller sample sizes and less standardized outcome definitions, did not materially alter the conclusions regarding caffeine’s superiority over other methylxanthines. Similarly, when analyses were limited to randomized controlled trials, the beneficial effects of caffeine on apnea reduction, extubation success, and bronchopulmonary dysplasia remained consistent.

The overall conclusions were also unchanged when studies with higher risk of bias or unclear blinding were considered separately. These consistency checks indicate that the synthesized findings are robust across study designs and time periods, supporting the reliability of the qualitative evidence base.

### 4.5. Efficacy in Apnea Reduction

Across the 25 included studies, methylxanthine therapy, particularly caffeine, consistently demonstrated a significant reduction in the frequency and severity of apnea episodes in preterm infants [8,36,38,39,43,44,45,46,47,48,49,50,51,52,53,54,55,56,57,58,59,60,61,62,63]. Most trials enrolled neonates born between 26–32 weeks’ gestation with mean birth weights below 1500 g, and caffeine citrate was the most frequently studied agent. The overall direction of effect was uniform, with all randomized controlled trials (RCTs) and observational studies reporting fewer apneic events compared with placebo or no treatment.

In the multicenter trial by Erenberg et al. (2000) [44], caffeine achieved a ≥50% reduction in apnea episodes in 68.9% of treated infants, confirming its superiority to placebo. The landmark CAP trial [8] further established caffeine’s efficacy, showing that among infants surviving to 36 weeks’ postmenstrual age, the proportion still requiring oxygen was significantly lower in the caffeine group (36%) than in placebo (47%), corresponding to an adjusted odds ratio of 0.63 (*p* < 0.001).

Early studies of theophylline and aminophylline demonstrated similar, though less predictable, improvements in apnea frequency. The Australian trial by Gupta et al. (1981) [40] reported a marked reduction in apneic events within 6–12 h of therapy initiation, and Murat et al. (1981) [41] observed near elimination of severe apnea after caffeine administration. The Swiss RCT by Bucher et al. (1988) [43] showed that caffeine reduced recurrent hypoxemic episodes in infants ≤ 32 weeks’ gestation, while Armanian et al. (2016) [46] confirmed its prophylactic value, with apnea developing in only 15% of caffeine-treated infants compared with 62% of controls.

In contrast, Fakoor et al. (2019) [53] found no preventive effect of caffeine in very preterm infants (≤32 weeks, ≤1500 g), though the study’s limited sample size and differences in baseline illness severity may explain the lack of statistical significance. Other trials such as Iranpour et al. (2022) [61] and Wei et al. (2016) [47] demonstrated additional respiratory benefits, with caffeine reducing both apnea frequency and the duration of non-invasive ventilation or NCPAP use.

When caffeine was compared directly with other methylxanthines, findings were generally consistent: Bairam et al. (1987) [42] found caffeine and theophylline to be equally effective but with fewer side effects in the caffeine group. More recent studies reinforced this safety advantage—Lin et al. (2022) [55] reported a significantly shorter treatment duration (11 vs. 17 days) and lower tachycardia rates (8.3% vs. 34.4%) for caffeine compared with aminophylline/theophylline. Conversely, Anggrainy et al. (2024) [57] noted a slightly greater reduction in daily apnea frequency with theophylline (2.28 ± 1.40 vs. 3.16 ± 1.31 episodes/day for caffeine), but this marginal difference was offset by longer oxygen and CPAP requirements in the theophylline group.

Collectively, these results confirm that methylxanthine therapy markedly decreases apnea frequency and severity, with caffeine citrate providing the most favorable balance of efficacy and tolerability. Despite some variability in reported effect sizes, the consistency of results across decades and study designs underscores a robust class effect.

Importantly, differences in apnea definitions and monitoring methods across studies reflect the evolution of clinical and technological practice over time. Early investigations relied on bedside clinical observation and basic monitoring [40,41], whereas more recent studies employed continuous cardiorespiratory monitoring with automated event detection [43,44,46,47,54,61]. This methodological heterogeneity explains some variation in quantitative outcomes but does not alter the overall conclusion that methylxanthines, and particularly caffeine, are effective in reducing apnea episodes and improving respiratory stability in preterm infants.

### 4.6. Respiratory Support and Extubation Success

Methylxanthine therapy, and particularly caffeine citrate, has demonstrated consistent benefits in reducing the duration and intensity of respiratory support required by preterm infants with apnea of prematurity [34,39,44,45,46,47,48,49,50,51,52,56,59,60,61,63,64,69,70]. Across randomized and observational studies, caffeine shortened the duration of mechanical ventilation and non-invasive respiratory assistance and increased the likelihood of successful extubation.

Early physiological investigations first suggested that methylxanthines improve lung mechanics. In Greenough et al. (1985) [34], oral theophylline significantly enhanced lung compliance (0.95 ± 0.42 vs. 0.67 ± 0.28 mL/cm H_2_O, *p* < 0.05) and accelerated ventilator weaning in infants between 24–33 weeks’ gestation, with the greatest benefit observed in those above 26 weeks. These early findings were later confirmed and expanded by the large Caffeine for Apnea of Prematurity (CAP) trial [8], which reported a one-week earlier discontinuation of positive airway pressure in the caffeine group (median 31.0 vs. 32.0 weeks postmenstrual age; IQR 29.4–33.0 vs. 30.3–34.0). This reduction in ventilatory support was accompanied by a significantly lower incidence of bronchopulmonary dysplasia (36.3% vs. 46.9%; adjusted OR 0.63, 95% CI 0.52–0.76; *p* < 0.001).

Subsequent studies have reinforced these findings, particularly when caffeine was administered early. Wei et al. (2016) [47] demonstrated that initiating caffeine within 24 h of life lowered peak inspiratory pressure and FiO_2_ requirements, shortened the duration of intubation and NCPAP, and reduced ventilator-associated pneumonia. Dekker et al. (2017) [48] observed that caffeine given in the delivery room enhanced minute ventilation and tidal volumes within minutes of birth, supporting its role in improving immediate postnatal respiratory effort. Similarly, Iranpour et al. (2022) [61] found that prophylactic caffeine in infants 1250–2000 g shortened the mean duration of NCPAP (41.5 vs. 78.5 h), while Armanian et al. (2016) [46] reported lower rates of apnea and bradycardia in caffeine-treated neonates.

Meta-analytic data also corroborate these individual findings. Kua and Lee (2017) [69] reviewed over 64,000 infants and concluded that caffeine initiated within the first 3 days of life significantly reduced bronchopulmonary dysplasia and facilitated earlier respiratory independence, albeit with a small, non-significant increase in mortality in some cohort studies. A more recent synthesis by Oliphant et al. (2024) [63] confirmed that caffeine at maintenance doses of 10–20 mg/kg/day effectively decreased intermittent hypoxemia and the need for supplemental respiratory support in late preterm infants.

In smaller comparative trials, other methylxanthines yielded mixed results. Durand et al. (1987) [70] and Barrington and Finer (1993) [64] found that aminophylline improved extubation success (78% vs. 53%) and reduced reintubation risk in infants under 1250 g, although these benefits were offset by higher rates of tachycardia and feeding intolerance. In contrast, Raza et al. (2024) [59] reported greater efficacy for caffeine (87% vs. 63% extubation success) and lower oxygen and ventilation requirements (27% vs. 57% and 3% vs. 17%, respectively).

Evidence across nearly four decades demonstrates that caffeine citrate enhances respiratory stability, reduces ventilator dependence, and improves extubation outcomes, with consistent results across diverse populations and gestational ages. These benefits are likely multifactorial, stemming from improved diaphragmatic contractility, enhanced central respiratory drive, and reduced ventilator-associated lung injury. The CAP trial demonstrated significant reduction in supplemental oxygen requirements with caffeine therapy. The primary outcome of bronchopulmonary dysplasia, defined as oxygen dependency at 36 weeks postmenstrual age, was significantly reduced in the caffeine group (36.3% vs 46.9%) [8].

This finding was particularly important as it represented the first demonstration that methylxanthine therapy could impact long-term respiratory outcomes beyond immediate apnea reduction. The mechanism for this benefit likely involves multiple factors including reduced ventilator-induced lung injury, improved respiratory muscle function, and enhanced lung development.

Results of trials based on individual studies evaluating the effect of methylxanthines for apnea of prematurity were tabulated to summarize key characteristics, including sample size, intervention, outcomes, and effect measures (Table 2).

### 4.7. Safety Profile and Adverse Effects

The safety profile of methylxanthines, particularly caffeine citrate, is well established across all included studies, with adverse events being infrequent, mild, and typically dose-related [8,34,41,44,50,51,52,55,56,59,60]. Overall, caffeine demonstrated a wider therapeutic window and lower toxicity than either theophylline or aminophylline, making it the preferred first-line agent for apnea of prematurity.

In the landmark CAP trial [8], which remains the most comprehensive safety evaluation to date, only 1.8% of infants required dose adjustment or discontinuation due to tachycardia or jitteriness (23 in the caffeine group vs. 14 in placebo). No significant differences were observed in mortality, necrotizing enterocolitis, intraventricular hemorrhage, or brain injury. Other large studies [48,49,69] similarly found no increase in major neonatal morbidities or neurological complications associated with caffeine therapy.

By contrast, earlier trials with theophylline or aminophylline reported higher rates of cardiovascular and gastrointestinal side effects. Greenough et al. (1985) [34] excluded two infants from the theophylline arm due to tachycardia and agitation, while later comparative studies such as Lin et al. (2022) [55] quantified these differences: tachycardia occurred in 8.3% of caffeine-treated infants versus 34.4% in the aminophylline/theophylline group. Habibi et al. (2019) [50] and Lookzadeh et al. (2019) [51] confirmed similar therapeutic efficacy between caffeine and aminophylline but emphasized caffeine’s better tolerability and lower recurrence of apnea-related side effects.

Gastrointestinal adverse events, including feeding intolerance and vomiting, were rare and typically transient. In the study by Erenberg et al. (2000) [44], gastrointestinal disorders occurred in both caffeine and placebo groups without significant differences. Elias-Jones et al. (1985) [71] likewise noted minimal gastrointestinal intolerance with theophylline, suggesting that such events are not clinically limiting at therapeutic doses.

Transient effects on growth have also been reported but appear self-limited. The CAP trial [1] recorded a temporary decrease in weight gain (−23 g at week 2) that resolved by week 6, with no differences in head circumference or length. These findings are consistent with subsequent studies reporting no sustained impact on growth trajectories [47,60,61].

Neurological adverse effects were rare and generally mild. No evidence from RCTs or follow-up studies, including the 11-year CAP follow-up [49], indicates increased risk of neurodevelopmental impairment; in fact, long-term pulmonary and cognitive outcomes were slightly improved in caffeine-treated cohorts.

Taken together, these data confirm that caffeine therapy for apnea of prematurity is both effective and safe, with a remarkably low incidence of clinically significant adverse events. Theophylline and aminophylline, while pharmacologically similar, require more frequent monitoring and dose adjustments due to their narrower therapeutic index and higher rate of side effects. The consistency of safety findings across four decades of studies underscores caffeine’s position as the most reliable and well-tolerated methylxanthine for preterm infants.

### 4.8. Comparative Effectiveness of Caffeine Versus Theophylline

Direct comparative studies between caffeine and other methylxanthines consistently demonstrate equivalent efficacy in apnea reduction, but superior safety, pharmacokinetic stability, and clinical practicality for caffeine [42,43,45,50,51,52,55,56,57,58,59]. Although theophylline played a pioneering role in the pharmacologic management of apnea of prematurity, cumulative evidence over the past four decades supports caffeine as the preferred agent.

Early controlled trials established the therapeutic potential of both drugs. Bairam et al. (1987) [42] reported similar reductions in cardiorespiratory abnormalities with caffeine and theophylline, though theophylline was associated with higher rates of tachycardia and gastrointestinal intolerance. The findings by Jeong et al. (2015) [45] confirmed comparable short-term efficacy between the two drugs for apnea control in preterm infants but likewise noted that caffeine offered a more favorable safety profile and simpler dosing.

More recent evidence reinforces these advantages. Lin et al. (2022) [55] demonstrated that caffeine therapy required a shorter duration of treatment (11 vs. 17 days) and was associated with lower rates of tachycardia (8.3% vs. 34.4%) than aminophylline/theophylline. In a randomized clinical trial, Anggrainy et al. (2024) [57] observed that theophylline slightly reduced daily apnea frequency more than caffeine (2.28 ± 1.40 vs. 3.16 ± 1.31 episodes/day); however, this benefit was offset by longer oxygen or CPAP use in the caffeine group, with no differences in hospital stay or feeding milestones. Bashar et al. (2024) [58] and Raza et al. (2024) [59] further confirmed equivalent efficacy between agents but reaffirmed caffeine’s lower oxygen and ventilation requirements, better extubation success (87% vs. 63%), and fewer cardiovascular adverse events.

From a pharmacological standpoint, caffeine’s advantages are clear. It has a longer half-life (~100 hours vs. 30 hours for theophylline), allowing for once-daily dosing and more stable plasma levels [27,28,29]. Its wider therapeutic window (8–20 µg/mL) minimizes the need for serum concentration monitoring, in contrast to theophylline, which requires frequent therapeutic drug monitoring to avoid toxicity [28,29]. Additionally, caffeine’s pharmacokinetic predictability and minimal hepatic metabolism in preterm infants reduce interindividual variability and drug–drug interactions.

All of these findings confirm that while theophylline remains effective, its narrow therapeutic index, complex metabolism, and higher incidence of adverse effects make it less suitable for routine neonatal use (Table 3). Caffeine offers comparable or superior efficacy with fewer safety concerns, more convenient dosing, and reduced monitoring demands. The evidence base has led to a clear preference for caffeine citrate in contemporary clinical practice. The combination of efficacy, safety, and dosing convenience has made caffeine the first-line methylxanthine for apnea of prematurity in most neonatal intensive care units worldwide [72].

### 4.9. Long-Term Outcomes

Long-term evidence from randomized and observational studies consistently indicates that methylxanthine therapy—especially caffeine citrate—confers durable respiratory and neurodevelopmental benefits without increasing adverse sequelae [8,46,47,48,49,61,69,75,76]. The most compelling data come from the Caffeine for Apnea of Prematurity (CAP) trial and its extended follow-ups, complemented by smaller studies assessing bronchopulmonary dysplasia (BPD), neurodevelopment, and growth.

The CAP trial [8] demonstrated not only short-term improvements in apnea reduction and earlier respiratory independence but also a significant decrease in BPD incidence (36.3% vs. 46.9%; adjusted OR 0.63, 95% CI 0.52–0.76; *p* < 0.001). Subsequent analyses identified additional cardiopulmonary benefits, including a lower need for pharmacologic or surgical patent ductus arteriosus (PDA) closure and reduced exposure to postnatal corticosteroids and doxapram. These effects likely reflect both direct respiratory stimulation and secondary prevention of ventilator-induced lung injury.

Follow-up studies confirm that caffeine’s benefits extend into later childhood. Doyle et al. (2017) [49], assessing former CAP participants at 11 years of age, found that caffeine-treated children had better expiratory flow rates (FEV_1_ z-score −1.00 vs. −1.53, *p* < 0.05) and improved pulmonary function compared with placebo, without evidence of adverse neurodevelopmental impact. Earlier CAP analyses also showed higher rates of survival without disability and lower incidences of cerebral palsy and cognitive delay [65].

Additional studies reinforce these findings. Wei et al. (2016) [47] and Iranpour et al. (2022) [61] reported that early caffeine administration reduced prolonged oxygen dependence and improved respiratory stability at discharge. The systematic review by Kua and Lee (2017) [69] similarly concluded that early caffeine initiation (<3 days postnatal age) was associated with lower BPD rates and shorter ventilation duration, supporting a lasting pulmonary benefit. Moreover, animal and mechanistic data suggest that caffeine may promote alveolarization, improve surfactant function, and reduce pulmonary inflammation [75,76].

Growth and developmental outcomes remain reassuring. The temporary reduction in early weight gain observed in the CAP trial [8] (−23 g at week 2) resolved by week 6, with no differences in head circumference or length thereafter. Long-term follow-up revealed no detrimental effects on growth, hearing, or vision, confirming the overall safety of therapy.

The longitudinal evidence supports caffeine as the only methylxanthine with proven long-term safety and efficacy, yielding measurable improvements in pulmonary and neurodevelopmental outcomes that persist into childhood. These data strengthen the recommendation for caffeine citrate as the standard of care for apnea of prematurity and as a cornerstone of lung-protective and neuroprotective neonatal strategies.

### 4.10. Dosing Strategies and Treatment Protocols

Evidence across clinical trials and pharmacologic studies demonstrates that caffeine citrate, the preferred methylxanthine for apnea of prematurity, is both effective and safe across a broad therapeutic range, requiring less frequent monitoring than theophylline or aminophylline [8,27,28,30,31,37,47,48,56,72,77,78]. The standard dosing regimen established by the CAP trial [8] has since become the global reference for neonatal care.

**Figure 4:**
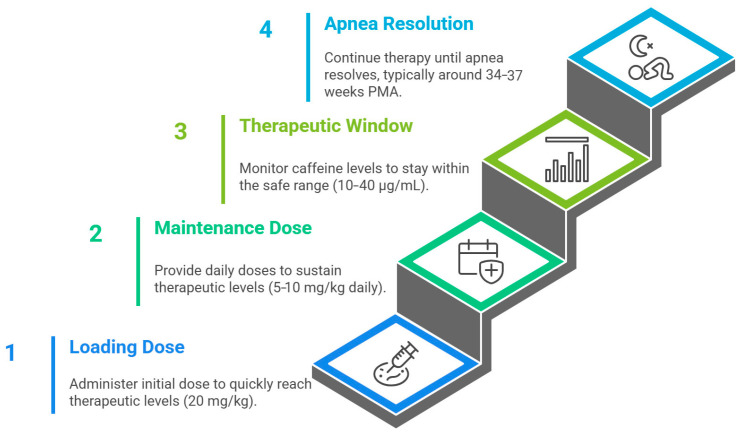
Standard caffeine therapy protocol for apnea of prematurity.

The CAP protocol defined an initial loading dose of 20 mg/kg caffeine citrate (equivalent to 10 mg/kg caffeine base), followed by a daily maintenance dose of 5 mg/kg caffeine citrate (2.5 mg/kg base) administered either intravenously or orally until resolution of apnea. Most infants received caffeine beginning within the first 10 postnatal days, with the median treatment duration of 37 days and discontinuation typically by 35 weeks’ postmenstrual age (Figure 4). This regimen achieves target plasma concentrations of 8–20 µg/mL—well within the therapeutic window—while avoiding levels above ~50 µg/mL, which are associated with toxicity (tachycardia, jitteriness, and feeding intolerance).

The CAP trial established the reference dosing regimen for caffeine citrate: a loading dose of 20 mg/kg (equivalent to 10 mg/kg caffeine base) followed by a maintenance dose of 5 mg/kg once daily, administered intravenously or orally until resolution of apnea, typically by 34–37 weeks postmenstrual age. Optimal therapeutic plasma concentrations range between 8–20 µg/mL, while levels exceeding 50 µg/mL may cause toxicity. Monitoring of heart rate, feeding tolerance, and growth ensures safe and individualized treatment.

Several studies have explored alternative dosing strategies. Wei et al. (2016) [47] and Dekker et al. (2017) [48] found that early caffeine administration, within the first 24 hours of life, improves extubation success and early respiratory drive without increased adverse effects. Similarly, Oliphant et al. (2023) [56] demonstrated that maintenance doses of 10–20 mg/kg/day significantly reduce intermittent hypoxemia events compared to placebo, whereas higher doses offered no additional benefit but slightly increased tachycardia incidence. Recent meta-analyses, including Oliphant et al. (2024) [63], confirm that while higher caffeine doses may enhance short-term respiratory outcomes, they do not confer additional neurodevelopmental benefits and may increase the risk of adverse events. These findings support the need for careful dose titration and selective use of TDM in vulnerable infants.

Theophylline and aminophylline require more complex regimens due to their shorter half-lives (approximately 30 hours in preterm infants) and narrow therapeutic index [28,29,31]. Dosing intervals are typically every 8–12 hours, with plasma concentration monitoring necessary to maintain levels within 6–12 µg/mL. These practical limitations and the higher risk of dose-related toxicity have contributed to the progressive replacement of theophylline by caffeine in modern NICUs.

Routine therapeutic drug monitoring (TDM) is generally unnecessary for caffeine because of its stable pharmacokinetics and predictable renal clearance. However, monitoring can be useful in cases of persistent apnea, suspected toxicity, or prolonged therapy. In this regard, validated analytical techniques such as liquid chromatography–mass spectrometry (LC–MS) developed at the Research Center of the Faculty of Pharmacy, Craiova, provide highly accurate quantification of caffeine and theophylline plasma levels in neonates [37]. These assays support individualized dosing and safe drug titration, particularly in extremely low birthweight infants or those with renal impairment.

Clinical monitoring remains essential and should include observation of heart rate, feeding tolerance, weight gain, and signs of overstimulation. Most studies recommend continuing treatment until the infant remains apnea-free for 5–7 days or reaches 34–37 weeks postmenstrual age, at which point therapy can be safely discontinued [8,47,78].

We can conclude that the optimal caffeine therapy protocol includes a 20 mg/kg loading dose and 5 mg/kg daily maintenance dose, initiated early in life and continued until stable respiratory control is achieved. Caffeine’s wide safety margin, once-daily dosing, and minimal need for drug-level monitoring make it the most practical and reliable methylxanthine regimen for neonatal use. Emerging evidence supports early initiation (<72 h) as part of a comprehensive, lung-protective respiratory strategy in preterm infants.

Overall, the evidence consistently supports the superior efficacy and safety of caffeine citrate compared with other methylxanthines. Across studies, caffeine reduced the frequency and duration of apneic episodes, facilitated successful extubation, and shortened the duration of mechanical ventilation and oxygen therapy [34,35,36,37,39,40,41,44,45,46,47,48,49,50,51,52,53,54,55,56,57,58,59,60,61,64,70,79]. The CAP trial [8] provided the most robust evidence, demonstrating a 36% vs. 47% incidence of bronchopulmonary dysplasia in caffeine versus placebo (adjusted OR 0.63, *p* < 0.001), along with earlier weaning from respiratory support.

In smaller trials, caffeine and theophylline showed similar short-term efficacy for apnea reduction [42,43,50,51,52], but caffeine consistently exhibited a lower rate of tachycardia and feeding intolerance [55,58]. Early initiation of caffeine (<72 h post-birth) was associated with shorter ventilation times and fewer hypoxemic episodes [47,48,63,69].

Long-term data from the CAP follow-up study [49] showed that children treated with caffeine had better pulmonary function (FEV_1_ z score = −1.00 vs. −1.53) and similar or improved neurodevelopmental outcomes at 11 years.

Safety outcomes were favorable across all studies: adverse effects were mild and transient, primarily limited to self-resolving tachycardia in a small fraction of infants (≤2 % in the CAP trial [8]). No study reported increased mortality or major neurological complications.

## 5. Discussion

This systematic review consolidates more than four decades of evidence demonstrating that methylxanthines—particularly caffeine citrate—significantly reduce the frequency and severity of apnea of prematurity (AOP). The findings extend beyond symptom control, revealing benefits for respiratory stability, bronchopulmonary dysplasia (BPD) prevention, and long-term pulmonary and neurodevelopmental outcomes [13,46,47,49,61,80].

However, the apparent consistency of benefit across studies masks substantial heterogeneity in methodology, intervention protocols, and population characteristics, which warrants careful interpretation. Early studies relied on small, single-center samples and variable definitions of apnea, often based on clinical observation rather than standardized cardiorespiratory monitoring. Later randomized controlled trials (RCTs), particularly the CAP trial [8], employed more rigorous designs, broader inclusion criteria, and contemporary respiratory management strategies. These differences partly explain the variability in reported effect sizes across the evidence base.

Another important source of heterogeneity lies in timing and dosing of methylxanthine therapy. Studies initiating caffeine within the first 24–72 h of life [47,48,69] consistently reported greater respiratory benefits and lower BPD incidence than trials beginning treatment later. Conversely, some negative or neutral findings, such as those of Fakoor et al. (2019) [53], may reflect delayed intervention or lower maintenance doses. These distinctions underscore that timing is a critical modifier of treatment efficacy.

Interstudy differences also stem from methylxanthine type. Theophylline and aminophylline exhibit similar pharmacologic actions to caffeine but differ in metabolism and toxicity profile. Their narrower therapeutic window and higher interindividual variability likely contribute to inconsistent results in older trials [34,41,42,50,51,52,58]. This pharmacokinetic variability highlights that the superiority of caffeine is not only clinical but also practical—stemming from more predictable dosing and fewer adverse effects.

Despite these variations, the direction of treatment effect remains remarkably consistent. Across randomized and observational data, all methylxanthines improved respiratory outcomes compared to placebo or no treatment, reinforcing the robustness of the class effect. The CAP trial [8,48,49] provides the highest-certainty evidence, confirming a substantial reduction in BPD and earlier discontinuation of respiratory support. Supporting trials and meta-analyses [63,69,75,76] further validate these findings across diverse settings, adding external generalizability to the CAP results.

Nonetheless, certain knowledge gaps remain. Few studies directly compared prophylactic versus therapeutic caffeine initiation, and data on extremely preterm infants (<26 weeks) remain limited. Moreover, most studies report short- to medium-term outcomes; long-term neurodevelopmental data are largely derived from a single cohort (the CAP follow-up at 11 years [49]). The relative contributions of pharmacologic stimulation versus improved respiratory mechanics to long-term pulmonary benefit also remain incompletely understood.

### 5.1. Clinical Implications

#### 5.1.1. Evidence-Based Practice Recommendations

The evidence strongly supports caffeine citrate as the methylxanthine of choice for treating AOP. The CAP trial dosing protocol (20 mg/kg loading, 5 mg/kg daily maintenance) remains the most validated regimen, supported by robust efficacy and safety data from over 2000 infants [8]. Importantly, results across multiple studies confirm that early initiation (<72 h postnatal age) provides the greatest benefit, likely by preventing the cascade of hypoxemia, ventilator dependence, and subsequent BPD [47,48,69].

While some studies (e.g., Anggrainy et al., 2024 [57]) reported marginally greater short-term apnea reduction with theophylline, such differences lack clinical significance when weighed against caffeine’s broader safety margin, longer half-life, and ease of administration [27,28,29,42,55]. The collective data thus justify caffeine as the first-line standard of care, while theophylline or aminophylline may serve as alternatives only when caffeine is unavailable.

#### 5.1.2. Implications for Neonatal Intensive Care Practice

Caffeine therapy has reshaped neonatal respiratory management. Its proven ability to reduce BPD and shorten ventilatory support duration has made it a core element of lung-protective strategies [75,76]. The once-daily dosing and minimal need for serum level monitoring enable consistent implementation even in resource-limited settings, supporting its inclusion in World Health Organization (2023) guidelines for preterm and low-birth-weight infant care [81].

At the same time, clinicians should recognize evidence gaps in optimal treatment duration and criteria for discontinuation. The CAP trial [8] ceased treatment around 35 weeks’ postmenstrual age, but emerging evidence suggests some infants may benefit from extended prophylaxis, as explored by Carlo et al. (2025) [60]. Systematic evaluation of tapering strategies and long-term effects of prolonged therapy remains a priority.

In comparison with recent systematic reviews, our findings are consistent with those reported by Oliphant et al. (2024), who conducted a comprehensive meta-analysis of 15 randomized controlled trials including over 3500 preterm infants [63]. Their review confirmed that caffeine therapy reduces the incidence of apnea and bronchopulmonary dysplasia and may have beneficial effects on long-term motor function. Notably, the authors reported that higher doses and earlier initiation of caffeine were associated with improved short-term respiratory outcomes without evidence of adverse neurodevelopmental effects. These observations support the potential advantages of administering caffeine within the first 72 h of life, as earlier exposure may enhance respiratory stability, facilitate extubation, and reduce the need for prolonged ventilatory support.

Our findings underscores the growing body of evidence suggesting that early caffeine administration may confer both pulmonary and neuroprotective benefits in preterm infants. Nonetheless, consistent with previous studies, further large-scale studies are needed to define the optimal dosing regimen and timing of initiation to balance efficacy and safety, particularly regarding the risk of tachycardia at higher doses.

Overall, this evidence base demonstrates a high level of certainty that caffeine improves short- and long-term outcomes in preterm infants with AOP, while evidence for other methylxanthines remains moderate and limited by older, smaller studies. The consistency of effect across diverse designs and populations, combined with strong biological plausibility and safety data, supports caffeine as a cornerstone of evidence-based neonatal care. Nevertheless, uncertainties remain regarding optimal dosing duration, prophylactic thresholds, and individualized response predictors. Addressing these gaps through large, contemporary RCTs and pharmacogenomic studies will be essential to refine the next generation of personalized neonatal respiratory support.

#### 5.1.3. Economic and Health System Implications

Although comprehensive cost analyses are limited, multiple studies, including the CAP trial [8], indicate substantial downstream healthcare savings associated with caffeine use. Reduced need for mechanical ventilation, doxapram, corticosteroids, and transfusions translates into lower NICU resource utilization. Given caffeine’s low acquisition cost and minimal monitoring requirements, its widespread adoption is both clinically and economically justified.

### 5.2. Mechanistic Insights and Future Directions

#### 5.2.1. Mechanistic Interpretation of Clinical Benefits

The consistent clinical benefits of caffeine therapy in apnea of prematurity (AOP) likely arise from a multifactorial mechanism of action that extends beyond central respiratory stimulation. At therapeutic concentrations, caffeine antagonizes adenosine A_1_ and A_2_A receptors in the brainstem, enhancing neural excitability of respiratory centers and improving responsiveness to hypercapnia [80]. This mechanism explains the immediate reduction in apneic spells observed across early trials [34,40,41].

However, accumulating evidence indicates that caffeine’s effects are not limited to acute respiratory drive. By increasing intracellular cyclic adenosine monophosphate (cAMP) and activating protein kinase A, caffeine enhances diaphragmatic contractility, reduces muscle fatigue, and improves coordination between inspiratory effort and airway patency [7,20]. These actions may explain the sustained reduction in mechanical ventilation duration and earlier extubation seen in multiple trials [8,47,48].

The unexpected reduction in bronchopulmonary dysplasia (BPD) observed in the CAP trial [8] highlights caffeine’s broader physiological impact. Experimental data suggest that caffeine attenuates oxidative stress and inflammation, modulates surfactant production, and promotes alveolar and vascular maturation in the developing lung [75,76]. These pleiotropic effects likely contribute to improved lung compliance and reduced ventilator-associated injury, positioning caffeine as both a respiratory stimulant and a lung-protective agent.

#### 5.2.2. Variability in Response and Sources of Uncertainty

Although the direction of benefit is consistent, response magnitude varies across gestational ages, doses, and treatment timing. Extremely preterm infants (<26 weeks) may have reduced adenosine receptor density and immature hepatic metabolism, potentially modifying pharmacodynamics and optimal dosing [55]. Also, male infants and those <30 weeks showed stronger therapeutic responses and fewer tachycardic events, suggesting sex- and maturity-related pharmacokinetic differences. These observations underscore the need for individualized dosing strategies based on developmental physiology and possibly genetic factors affecting caffeine metabolism (e.g., CYP1A2 activity).

Variability in study outcomes also reflects differences in formulations, monitoring technologies, and co-interventions such as respiratory support modes or concurrent steroid therapy. These confounders highlight that caffeine’s benefits should be interpreted within the context of evolving neonatal care practices rather than as a fixed drug effect.

Despite robust short-term evidence, long-term mechanistic links between neonatal caffeine exposure and later pulmonary or neurocognitive outcomes remain incompletely characterized. Only a few studies, notably Doyle et al. (2017) [49], provide extended follow-up data. There is also limited understanding of the dose–response curve beyond the CAP regimen, particularly for high-dose or prolonged therapy.

Other research gaps include:

- Lack of standardized biomarkers to monitor caffeine responsiveness and optimal discontinuation timing.
- Minimal data on extremely low birth-weight (<1000 g) infants, who may require altered dosing intervals.
- Unclear impact of concomitant medications and environmental factors (hypoxia, infection, nutrition) on caffeine efficacy.
- Absence of large-scale pharmacogenomic studies to identify genetic determinants of treatment variability.

#### 5.2.3. Directions for Future Research

Future investigations should move beyond efficacy confirmation toward mechanistic precision and personalization. Priority areas include:

Pharmacokinetic and pharmacodynamic modeling in very and extremely preterm infants to optimize individualized dosing schedules.

Mechanistic studies exploring how adenosine blockade and cAMP signaling influence alveolar and neural development, clarifying the biological basis of reduced BPD and improved neurodevelopment.

Longitudinal cohort follow-ups evaluating neurocognitive, pulmonary, and cardiovascular outcomes into adolescence and adulthood.

Biomarker and therapeutic drug monitoring integration, using advanced assays such as LC-MS quantification [37], to support real-time dose adjustment.

Comparative effectiveness and cost-utility analyses across global neonatal care settings, ensuring equitable application of caffeine therapy in resource-limited environments.

Emerging research on next-generation adenosine receptor modulators may refine the therapeutic profile of methylxanthines, offering greater receptor selectivity and fewer systemic effects. Until such agents are validated, caffeine citrate remains the most evidence-based, accessible, and safe pharmacologic intervention for apnea of prematurity.

### 5.3. Limitations and Methodological Considerations

#### 5.3.1. Limitations of the Review Process

Several limitations of the review process should be acknowledged. Although comprehensive database searches were performed in PubMed, Embase, and Web of Science, the review may still be subject to publication bias, as unpublished or non-indexed studies could have been missed. A major limitation is the absence of quantitative synthesis: due to substantial clinical and methodological heterogeneity—particularly in dosing regimens, timing of therapy, definitions of apnea, and outcome measurement—meta-analysis was not feasible. As a result, the review relies on narrative synthesis, which may reduce precision and limit direct comparability across studies.

#### 5.3.2. Study Quality and Risk of Bias

The evidence base is dominated by a single large, high-quality trial (CAP trial) [8], with the remaining studies being smaller and of variable methodological quality. While this provides strong evidence for caffeine efficacy, it also creates dependence on a single study for many key conclusions. The smaller studies included in this review demonstrated variable risk of bias, particularly regarding allocation concealment, blinding procedures, and outcome reporting. No formal GRADE-based quantitative assessment of certainty was conducted, and evidence confidence was summarized qualitatively. Despite these limitations, the methodological process adhered to PRISMA 2020 standards, and the conclusions reflect consistent trends across high-quality studies.

These limitations must be considered when interpreting findings from individual studies, though the consistency of results across studies strengthens overall conclusions.

#### 5.3.3. Outcome Definition Variability

Inter-study variability represents an additional constraint. Differences in gestational age ranges, illness severity, co-interventions, monitoring techniques, and study design likely contributed to variation in reported effect sizes. Moreover, many included studies had relatively small sample sizes, reducing statistical power and increasing susceptibility to random error. These factors may limit the generalizability of some findings.

Significant variability in apnea outcome definitions across studies complicates direct comparison and meta-analysis. Early studies relied on clinical observation and basic monitoring, while later studies used more sophisticated cardiorespiratory monitoring systems. This evolution in monitoring technology likely improved outcome measurement accuracy but creates challenges for systematic review and meta-analysis [63].

Standardization of outcome definitions for future trials would facilitate more robust evidence synthesis and improve the quality of systematic reviews in this field.

#### 5.3.4. Population Heterogeneity

The included studies encompassed diverse populations in terms of gestational age, birth weight, and severity of illness. While this diversity enhances generalizability, it also introduces heterogeneity that may obscure important subgroup differences in treatment response [82].

Future studies should consider stratified analyses based on gestational age, birth weight, and illness severity to identify populations that may benefit most from methylxanthine therapy or require modified treatment approaches.

### 5.4. Gaps in Current Knowledge

The subgroup findings by Lin et al. (2022) [55] raise important questions about optimal methylxanthine selection in different populations. While they identified male sex and younger gestational age as predictors of better caffeine response, the mechanisms underlying these differences remain unclear. Future studies should prospectively validate these subgroup differences and explore whether pharmacokinetic variations, particularly in caffeine metabolism rates between sexes and gestational ages, explain the differential treatment responses.

#### 5.4.1. Optimal Treatment Duration

While the CAP trial provided guidance on treatment duration (median 37 days), optimal duration for individual patients remains unclear [8]. Some infants may benefit from shorter treatment courses, while others may require extended therapy. Research into biomarkers or clinical predictors that could guide individualized treatment duration would be valuable [37].

Long-term follow-up data from Doyle et al. (2017) showed that children at 11 years who had been exposed to neonatal caffeine in the CAP trial had better expiratory flows and lower rates of restrictive lung disease, indicating durable pulmonary benefits [49].

The systematic review by Kua and Lee (2017) adds weight to early initiation of caffe ine as a standard strategy, while highlighting unresolved safety questions regarding timing and population subgroups [69].

#### 5.4.2. Combination Therapies

The potential for combination therapies that might enhance the benefits of methylxanthine treatment remains largely unexplored. Combinations with other respiratory stimulants, anti-inflammatory agents, or neuroprotective compounds could potentially provide additive benefits [28,83,84].

#### 5.4.3. Long-Term Safety

While short-term safety data from the CAP trial are reassuring, long-term safety data beyond early childhood are limited [8]. Given the widespread use of caffeine therapy in preterm infants, continued long-term follow-up studies are important to ensure that benefits are sustained and no late adverse effects emerge [65].

### 5.5. Research Priorities

#### 5.5.1. Mechanistic Studies

Research into the mechanisms underlying caffeine’s protective effects against bronchopulmonary dysplasia and improved neurodevelopmental outcomes could inform optimized treatment strategies. Studies using animal models and human tissue samples could provide insights into direct effects on lung and brain development [13].

#### 5.5.2. Personalized Medicine Approaches

The findings by Lin et al. (2022) [55] represent an important step toward personalized methylxanthine therapy. The identification of specific subgroups—male infants and those < 30 weeks’ gestation—who derive greater benefit from caffeine suggests that methylxanthine selection should consider individual patient characteristics rather than applying a one-size-fits-all approach. The sex-based differences may relate to known variations in caffeine metabolism, with female infants demonstrating higher metabolic rates that could influence drug efficacy and adverse effect profiles.

Investigation of genetic polymorphisms affecting caffeine metabolism and response could lead to personalized dosing strategies. Pharmacogenomic studies in preterm infants could identify subpopulations requiring modified treatment approaches.

#### 5.5.3. Novel Delivery Methods

Research into novel delivery methods, such as inhaled caffeine or sustained-release formulations, could potentially enhance therapeutic benefits or reduce systemic exposure and adverse effects [85].

### 5.6. Clinical Practice Implications

The evidence strongly supports the following clinical practice recommendations for the management of apnea of prematurity:

- Caffeine citrate should be considered first-line therapy for apnea of prematurity in preterm infants.
- A standard dosing protocol consisting of a 20 mg/kg loading dose followed by a 5 mg/kg daily maintenance dose is well established.
- The therapeutic window for caffeine is wide but should not exceed 50 µg/mL to avoid potential toxicity [8,86,87].
- Routine monitoring of plasma methylxanthine levels can facilitate individualized therapy and ensure that therapeutic effects are achieved safely and effectively.
- The LC–MS method validated at the Research Center of the Faculty of Pharmacy in Craiova can be employed to determine real plasma titers of caffeine and theophylline in infants with apnea of prematurity, supporting personalized dosing strategies.
- Early initiation of methylxanthine therapy within the first days of life provides optimal benefits in reducing apnea frequency, facilitating extubation, and lowering bronchopulmonary dysplasia risk.
- Treatment duration should continue until apnea episodes resolve, typically by 34–37 postmenstrual weeks of age.

Beyond these recommendations, the principles and interventions discussed can be readily translated into everyday neonatal practice, including resource-limited settings. Caffeine therapy is cost-effective, widely available, and can be administered using simple oral or intravenous formulations requiring minimal monitoring. Incorporating caffeine therapy into kangaroo mother care, early feeding, and thermal stability programs maximizes its impact while maintaining feasibility. Standardized dosing protocols and nurse-led monitoring systems can ensure safe and effective therapy even in facilities with limited technology. These practical strategies align closely with the World Health Organization 2023 recommendations for care of preterm or low birth weight infants [81], which advocate for feasible, evidence-based interventions to improve neonatal survival and developmental outcomes globally.

### 5.7. Take-Home Messages

- Caffeine citrate should be considered first-line treatment for apnea of prematurity in preterm infants.
- A standard dosing regimen of 20 mg/kg loading dose followed by 5 mg/kg daily maintenance is well established and widely validated.
- The therapeutic window for caffeine is broad but should not exceed 50 µg/mL to prevent toxic side effects [8,86,87].
- Selective monitoring in infants with variable metabolism or clinical instability ensures of plasma methylxanthine levels enables individualized dosing that therapeutic effects remain within safe limits.
- The LC–MS method validated at the Research Center of the Faculty of Pharmacy, Craiova, can be used to determine accurate plasma titers of caffeine and theophylline in infants with apnea of prematurity.
- Early initiation of methylxanthine therapy within the first 72 h of life yields optimal respiratory and neurodevelopmental benefits.
- Therapy should be continued until apnea episodes resolve, usually between 34 and 37 postmenstrual weeks of age.
- Integration of caffeine therapy into kangaroo mother care, early feeding, and thermal regulation programs enhances its feasibility and impact in resource-limited settings.
- The long-term benefits of caffeine—improved neurological, respiratory, and growth outcomes—support its inclusion in international guidelines such as the World Health Organization (2023) Recommendations for Care of Preterm or Low Birth Weight Infants [81].

## 6. Conclusions

Caffeine therapy remains an essential component of neonatal care for the prevention and treatment of apnea of prematurity. Our findings indicate that the early initiation of caffeine within the first 72 h of life may be associated with improved respiratory stability and reduced risk of bronchopulmonary dysplasia with minimal adverse effects, consistent with recent meta-analyses. The strength of evidence, anchored by the CAP trial, supports caffeine as the first-line methylxanthine and a key component of modern neonatal respiratory care. However, while current evidence supports its routine use, uncertainties remain regarding the optimal dosing strategy, treatment duration, and safety profile in extremely preterm infants. The heterogeneity among existing studies and the limited number of long-term follow-ups highlight the need for further large-scale randomized trials to define the most effective and safe regimens. Therefore, caffeine should continue to be used judiciously as part of individualized neonatal care, with ongoing evaluation of dosing intensity and treatment duration to optimize outcomes while minimizing potential adverse effects.

## Figures and Tables

**Table 1:** Synthesis of outcome measures by clinical endpoint. BPD: Bronho-Pulmonary Dysplasia; CAP: Caffeine for Apnea of Prematurity; CI: Confidence Interval; CPAP: Continous Positive Airway Pressure; FEV1: Forced Expiratory Volume in 1 s; FiO_2_: Fraction of inspired Oxygen; IH: Intermitent Hypoxemia; IQ: Intelligence coefficient; OR: Odds Ratio; PDA: Patent Ductus Arteriosus; PIP: Peak Inspiratory Pressure; RR: Risk Ratio; SpO_2_: Partial pressure of the oxygen.

Outcome Category	Definition/Measurement	Effect Measures Reported in Included Studies	Summary of Findings (Synthesis)
Apnea Reduction	Reduction in frequency or severity of apneic episodes (>15–20 s cessations) measured via cardiorespiratory monitoring.	Proportion achieving ≥ 50% reduction [44], mean difference in episodes/day [40,57], RR or OR where available.	All studies reported significant reductions in apnea frequency with methylxanthines vs. placebo. Example: CAP trial [8]: Caffeine significantly reduced apnea and oxygen need; Erenberg trial [44] showed ≥50% reduction in apnea episodes (68.9% success). Overall qualitative synthesis supports caffeine as superior to placebo and equivalent or better than theophylline.
Bronchopulmonary Dysplasia (BPD)	Oxygen dependency at 36 weeks postmenstrual age.	Adjusted OR for BPD occurrence (CAP trial) [8].	CAP trial: OR = 0.63 (95% CI 0.52–0.76; *p* < 0.001) for BPD in caffeine vs. placebo (36.3% vs. 46.9%). Early caffeine administration associated with reduced BPD incidence in meta-analyses [63].
Mechanical Ventilation Duration	Duration (hours/days) of invasive ventilation or non-invasive support (CPAP, NCPAP).	Mean difference or median difference in ventilation days between caffeine vs. control [8,34,47].	Caffeine group had ≈1-week earlier discontinuation of positive pressure support (CAP trial). Theophylline reduced ventilation duration by ~33 h [34]. Early caffeine: shortened intubation and CPAP times [47].
Extubation Success	Successful discontinuation of ventilation without reintubation within 48–72 h.	Proportion (%) success or RR.	Extubation success 78% vs. 53% with aminophylline [64]. Early caffeine improved extubation outcomes (lower FiO_2_, lower PIP) [47]. Pooled qualitative synthesis—consistent improvement in extubation success with methylxanthines.
Intermittent Hypoxemia (IH)	Events with ≥10% SpO_2_ drop from baseline per hour.	Rate ratio (events/hour) or mean difference.	Caffeine 10–20 mg/kg/day reduced IH frequency significantly vs. placebo. Rate ratio ~0.65 vs. placebo [56].
Mortality	All-cause mortality during hospitalization or follow-up.	RR where reported (CAP trial).	No significant difference in mortality between caffeine and placebo (CAP trial; RR ≈ 1.0).
Neurodevelopmental Outcomes	Cognitive delay, cerebral palsy, deafness, blindness at 18–21 months or later.	Composite OR for adverse neurodevelopment (CAP trial), Mean z-scores for FEV1, IQ at 11 years [49].	CAP trial follow-up (18–21 months): no increase in adverse outcomes; long-term follow-up showed better pulmonary function (FEV1 z-score −1.00 vs. −1.53) and trend to improved cognitive scores [65].
Adverse Effects (Tachycardia, GI, Neurological)	Incidence of side effects requiring dose reduction or discontinuation.	Proportion (%) affected.	CAP trial: 1.8% required dose reduction (tachycardia/jitteriness). Tachycardia 8.3% (caffeine) vs. 34.4% (aminophylline/theophylline). Mild GI effects; no serious neurological toxicity [55].
Growth Parameters	Weight gain and head circumference change over treatment.	Mean difference (grams).	CAP trial: temporary ↓ weight gain (−23 g at week 2), normalized by week 6. No difference in head growth.
Patent Ductus Arteriosus (PDA) Closure	Need for pharmacologic/surgical PDA treatment.	Proportion (%) receiving PDA therapy.	CAP post hoc: significantly fewer infants required PDA closure in caffeine group. OR ≈ 0.68 (*p* < 0.05).
Length of Hospital Stay	Days from randomization to discharge.	Median difference (days).	No significant difference in length of stay (median 18 vs. 16.5 days) [60].

**Table 2:** Summary of included studies with key characteristics, outcomes, effect estimates, and overall risk-of-bias rating. AOP: Apnea of Prematurity; BPD: Bronho-Pulmonary Dysplasia; BW: Body Weight; CAP: Caffeine for Apnea of Prematurity; CPAP: Continous Positive Airway Pressure; FEF: Forced Expiratory Flow; FEV1: Forced Expiratory Volume in 1 s; FiO_2_: Fraction of inspired Oxygen; FVC: Forced Vital Capacity; IH: Intermitent Hypoxemia; MRI: Magnetic Resonance Imaging; NEC: Necrotizing EnteroColitis; NICU: Neonatal Intensive Care Unit; PDA: Persistent Ductus Arteriosus; PEEP: Positive End-Expiratory Pressure; PIP: Peak Inspiratory Pressure; PVL: PeriVentricular Leukomalacia; RCT: Randomized Control Trial; RDS: Respiratory Distress Syndrome; SD: Standard Deviation; SpO_2_; Partiap pressure of the Oxygen.

No.	Study (Author, Year)	Study Design	Population	Intervention	Comparator	Primary Outcome(s)	Key Findings	Risk-of-Bias Rating (Low/Some Concerns/High)
1.	Aranda et al. (1983) [39]	Non-controlled study	18 preterm neonates with recurrent apneic spells; mean birth weight: 1065.0 g; mean gestational age: 27.5 weeks.	Caffeine citrate, loading dose of 20 mg/kg intravenously, followed by a maintenance dose of 5 to 10 mg/kg once daily.	No comparator/control group; this was a non-controlled study.	Frequency of apneic episodes.	Caffeine significantly reduced mean apnea episodes (13.6 → 2.1/day) and improved ventilation parameters.	Some concerns
2.	Gupta et al. (1981) [40]	Randomized, double-blind, placebo-controlled trial	29 premature infants (15 in theophylline group, 14 in placebo group) with 4 or more apnoeas in a 12 h period.	Theophylline, initially 4 mg/kg six-hourly via nasogastric tube, increased to 6 mg/kg if no clinical response.	Placebo-same base as theophylline.	Decrease in the number of apneic episodes within 6–12 h of commencement of therapy.	Theophylline reduced apnea frequency within 6–12 h; 10/15 infants had no further episodes within 48 h.	Some concerns
3.	Murat et al. (1981) [41]	Prospective controlled study	Eighteen preterm infants (29 to 35 weeks’ gestation).	Caffeine sodium citrate (20 mg/kg loading dose, 5 mg/kg daily maintenance dose).	Control group (no treatment, but received caffeine if condition worsened).	Change in frequency and severity of mild and severe apnea events.	Caffeine decreased both severe and mild apnea vs. control; no treatment failures occurred.	High
4.	Greenough et al. (1985) [34]	Double-blind randomized trial	40 preterm, ventilated infants (gestational ages 24–33 weeks, mean gestational age 29.8 ± 2.4 weeks in theophylline group.	Oral theophylline solution (5 mg/mL); loading dose: 1 mL/kg (5 mg/kg theophylline); maintenance dose: 1 mL/kg per day.	Placebo (vehicle alone) solution, administered orally via nasogastric tube.	Lung compliance and duration of mechanical ventilation.	Theophylline improved lung compliance and shortened ventilation duration compared to placebo.	Low
5.	Bairam et al. (1987) [42]	Double-blind randomized controlled trial	20 premature infants with idiopathic apnea; theophylline group (n = 10): birth weight 1.5 ± 0.3 kg.	Theophylline: loading dose 6 mg/kg, maintenance dose 2 mg/kg every 12 h administered intravenously.	Caffeine: loading dose 10 mg/kg, maintenance dose 1.25 mg/kg every 12 h, administered intravenously.	Frequency of apnea ≥ 15 s with bradycardia < 80 bpm in idiopathic AOP.	Both theophylline and caffeine decreased cardiorespiratory abnormalities; theophylline caused more side effects.	Some concerns
6.	Bucher et al. (1988) [43]	Randomized controlled trial	50 spontaneously breathing, preterm infants of 32 weeks’ gestation or less.	Caffeine citrate (loading dose 20 mg/kg, maintenance dose 10 mg/kg per day).	Placebo (NaCl 0.9%)	Proportion of infants with recurrent hypoxaemic episodes (>20% fall in SpO_2_).	Caffeine reduced recurrent hypoxemic episodes compared with placebo from 57% to 51%, though effect size was modest.	Some concerns
7.	Erenberg, et al. (2000) [44]	Multicenter, double-blind, placebo-controlled study with an open-label rescue phase	82 preterm infants (28 to 32 weeks post conceptual age) with >6 apnea episodes >20 s per 24 h.	Caffeine citrate: IV loading dose of 10 mg/kg with daily dose of 2.5 mg/kg caffeine base, followed by 3 mg/kg daily.	Placebo	Reduction ≥ 50% in apnea frequency.	≥50% reduction in apnea achieved in 68.9% of caffeine group vs. placebo; consistent improvement over 10 days.	Some concerns
8.	Schmidt et al. (2006) [8]	Multicenter, randomized, double-blind, placebo-controlled trial (ClinicalTrials.gov, No. NCT00182312/22.03.2018	2006 preterm infants with birth weights 500–1250 g, enrolled within first 10 days of life	Caffeine citrate: loading dose 20 mg/kg IV, maintenance 5 mg/kg daily (increased to 10 mg/kg if needed) until apnea resolved.	Placebo (normal saline)	Composite of death cerebral palsy, cognitive delay, deafness, or blindness at 18–21 months; secondary—BPD and short-term morbidities.	Caffeine reduced BPD (36% vs. 47%; OR 0.63, *p* < 0.001), shortened ventilation by ~1 week, and lowered PDA treatment rates; no significant differences in mortality, NEC, or brain injury.	Low
9.	Jeong et al. (2015) [45]	Retrospective study	143 infants born at less than 33 weeks of gestation (Caffeine group: n = 54, Theophylline group: n = 89).	Caffeine group (n = 54): loading dose of 20 mg/kg intravenously for 30 min, followed by 5–8 mg/kg intravenously once a day.	Theophylline group (n = 89): loading dose of 5–7 mg/kg intravenously for 30 min, followed by 1.5–2 mg/kg every 8 h.	Apnea frequency, adverse effects, and major neonatal morbidity (e.g., BPD, PVL).	Caffeine and theophylline showed similar short-term efficacy for apnea control.	Moderate
10.	Armanian et al. (2016) [46]	Single-center, double-blind, placebo-controlled, randomized clinical trial (IRCT.ir (No. IRCT2013110610026N3).	52 premature infants with a birth weight of ≤ 1200 g and spontaneous breathing at 24 h of life.	Caffeine administered intravenously; a loading dose of 20 mg/kg was given on the first day of life, followed by a daily maintenance dose.	Placebo group (n = 26) received an equivalent volume of normal saline daily for the first 10 days of life.	Incidence of apnea, bradycardia, and cyanosis during the first 10 days of life.	Caffeine prophylaxis lowered apnea incidence (15% vs. 62%) and reduced bradycardia events.	Some concerns
11.	Wei et al. (2016) [47]	Prospective controlled clinical trial	59 preterm infants with RDS requiring mechanical ventilation; gestational age 27–33 + 6 weeks, birth weight < 1500 g.	Caffeine (citrate) intravenously; caffeine group: 20 mg/kg loading dose at 12–24 h after birth, followed by 8 mg/kg daily maintenance dose.	Control group (n = 29) received caffeine 4–6 h before planned extubation (same dosage as caffeine group).	Respirator parameters (PIP, PEEP, FiO2, intubation time, NCPAP time, oxygen time), incidence of complications.	Early caffeine reduced ventilator pressures, oxygen needs, and ventilator-associated pneumonia incidence.	Some concerns
12.	Dekker (2017) [48]	Randomized controlled trial	30 preterm infants (23 analyzed) of 24–30 weeks’ gestation; birth weight was 870 g in the caffeine group and 960 g the comparison group.	A loading dose of caffeine base (10 mg/kg) administered in the delivery room within the first 7 min after birth.	Infants who received a loading dose of caffeine base (10 mg/kg) later, after arrival in the NICU.	Respiratory effort—minute and tidal volumes during initial ventilation.	Delivery-room caffeine improved minute (189 vs. 162 mL/kg/min) and tidal volumes (5.2 vs. 4.4 mL/kg) during initial neonatal ventilation.	High
13.	Doyle et al. (2017) [49]	RCT–follow-up of the CAP trial (ClinicalTrials.gov, No. NCT00182312/22.03.2018)	142 children (74 caffeine, 68 placebo) from the CAP trial, born with birth weight less than 1251 g (range 500–1250 g).	Caffeine citrate 20 mg/kg loading dose and 5–10 mg/kg/d maintenance dose.	Placebo (saline)	Pulmonary function at 11 years (FEV_1_, FVC, FEV_1_/FVC, FEF_25_–_75_).	Former caffeine-treated infants had better expiratory flow rates (FEV_1_ z –1.00 vs. –1.53) at 11 years.	Some concerns
14.	Habibi et al. (2019) [50]	Double-blind randomized clinical trial	67 premature neonates with idiopathic apnea of prematurity; inclusion criteria: gestational age < 37 weeks.	Aminophylline: intravenous, 5–7 mg/kg loading dose, then 1–2 mg/kg every 6–12 h. Caffeine: intravenous, 20 mg/kg loading dose and 5–10 mg/kg/d maintenance dose.	Caffeine recipient group (n = 36).	Recurrent apnea frequency and short-term drug side effects.	Aminophylline and caffeine had similar efficacy and side-effect profiles; minimal recurrence of apnea.	Some concerns
15.	Lookzadeh et al. (2019) [51]	Randomized Clinical Trial (RCT) (IR code IRU.MEDICINE.REC.1397.78)	80 premature neonates (43 male, 37 female) with gestational age under 34 weeks.	Caffeine (initial dose of 30 mg/kg, 24 h maintenance dose of 10 mg/kg).	Aminophylline (initial dose of 5 mg/kg, maintenance dose of 2 mg/kg every 8 h).	Apnea frequency, oxygen/CPAP requirement, and adverse events.	No significant difference in the frequency of apnea or oxygen need between the two groups.	Some concerns
16.	Zulqarna et al. (2019) [52]	Randomized Control Trial	100 infants (50 in Theophylline group, 50 in Caffeine group).	Theophylline: loading dose 4.8 mg/kg intravenously in 30 min, maintenance dose 2 mg/kg.	Theophylline group compared to Caffeine group.	Daily apnea episodes and correlation with methylxanthine serum levels.	Daily apnea rates improved after caffeine treatment compared with theophylline.	Some concerns
17.	Fakoor et al. (2019) [53]	Clinical–experimental trial	100 very preterm infants with a gestational age ≤ 32 weeks and a birthweight ≤ 1500 g.	Group A (50 infants) received 20 mg/kg of venous caffeine on the 2nd day of birth (24–48 h), followed by a maintenance.	Group B (50 infants) did not receive caffeine.	Prevention of apnea episodes in very preterm infants.	There was no significant difference in the incidence of apnea between the caffeine group (14%) and the control group (18%).	Some concerns
18.	Liu et al. (2020) [54]	Randomized controlled trial (RCT)	Total of 194 preterm infants (≤32 weeks’ gestational age) initially; 160 infants included in final analysis.	Caffeine, administered within 72 h after birth.	Placebo group (n = 93)	White-matter maturation and apnea/ventilation duration.	Caffeine reduced apnea and ventilation duration and improved white-matter development on MRI.	Some concerns
19.	Lin et al. (2022) [55]	Retrospective case–control gestational age-matched study	144 premature infants (48 in caffeine group, 96 in aminophylline/theophylline group).	Caffeine: loading dose 20 mg/kg, maintenance 5 mg/kg/dose once per day, titrated by 10 mg/kg/dose per day.	Aminophylline/Theophylline group	Treatment duration and incidence of tachycardia (≥160 bpm).	Caffeine required shorter treatment (11 vs. 17 days) and caused less tachycardia (8% vs. 34%).	Some concerns
20.	Iranpour (2022) [61]	Randomized Controlled Trial	90 neonates (birth weight between 1250 and 2000 g) clinically diagnosed with RDS.	Caffeine: 20 mg/kg initial dose, then 10 mg/kg daily maintenance dose.	Control group (no placebo or similar drugs).	Duration of NCPAP respiratory support.	Caffeine shortened NCPAP duration (41 h vs. 78 h) compared with control.	Some concerns
21.	Oliphant (2023) [56]	Phase IIB, double-blind, five-arm, parallel, randomized controlled trial. (anzctr.org.au, no. ACTRN12618001745235/24.10.2018	132 late preterm infants born at 34 + 0–36 + 6 weeks’ gestation, with a mean (SD) birth weight of 2561 (481) g.	Caffeine citrate: infants were randomly assigned to receive a loading dose (10, 20, 30 or 40 mg/kg) followed by a daily maintenance dose.	Placebo group receiving an equivolume enteral solution.	Rate of intermittent hypoxemia events per hour.	Caffeine 10–20 mg/kg/day reduced IH events vs. placebo.	Some concerns
22.	Anggrainy (2024) [57]	Randomized clinical trial	Fifty premature neonates (gestational age 28–34 weeks, birth weight < 2500 g) with apnea of prematurity (AOP).	Oral caffeine citrate with an initial dose of 20 mg/kg BW, followed by a maintenance dose of 5–10 mg/kg BW/day.	Oral theophylline with an initial dose of 5–8 mg/kg, followed by 4–22 mg/kg BW every 6–8 h for seven days.	Mean daily apnea frequency after treatment.	Theophylline slightly reduced apnea episodes more than caffeine (3.16 vs. 2.28), but with longer CPAP use.	Some concerns
23.	Bashar (2024) [58]	Prospective, open-label, randomized controlled trial	55 preterm neonates (≤34 weeks’ gestational age); mean gestational age: 31.3 ± 2.1 weeks (range 26–34 weeks).	Caffeine (loading dose of 20 mg/kg, followed by maintenance doses of 5 mg/kg/day).	Aminophylline (loading dose of 5 mg/kg, followed by maintenance doses of 2 mg/kg every 8 h).	Comparative efficacy and safety of caffeine vs. aminophylline for AOP prophylaxis/treatment.	Caffeine and aminophylline had similar efficacy and safety; no major outcome differences.	High
24.	Raza (2024) [59]	Comparative randomized study	A total of 60 premature newborn babies (<37 weeks’ gestation) with their first apnea episode.	Caffeine (intravenous, 20 mg/kg anhydrous caffeine loading dose, 5 mg/kg maintenance every 24 h).	Aminophylline (intravenous, 5 mg/kg loading dose, 1.5 mg/kg maintenance every 12 h).	Reappearance of apnea within ≥3 days after initial episode.	Caffeine showed higher efficacy (87% vs. 63%), less oxygen need, and fewer reintubations than aminophylline.	High
25.	Carlo (2025) [60]	Randomized clinical trial (Clinicaltrials.gov, no. NCT03340727)	827 infants born at 29 to 33 weeks’ gestation (median gestational age, 31 weeks; 414 female [51%]).	Oral caffeine citrate 10 mg/kg/day	Placebo	Time to hospital discharge after randomization.	Extended caffeine therapy did not reduce hospital stay (18 vs. 16.5 days).	Low

**Table 3:** Comparative pharmacokinetic characteristics of caffeine versus theophylline for apnea management in preterm infants [73,74].

Characteristic	Caffeine	Theophylline
Half-life	Longer half-life	Shorter half-life
Dosing Frequency	Once-daily dosing	More frequent dosing required
Therapeutic Window	Wider therapeutic window with reduced toxicity risk	Narrower therapeutic window
Pharmacokinetic Predictability	More predictable pharmacokinetics in preterm infants	More complex metabolism with age-related changes
Drug Interactions	Minimal drug interactions	Greater potential for drug interactions

## Data Availability

No data was generated for this article.

## Supplementary Materials

The following supporting information can be downloaded at: https://www.mdpi.com/article/10.3390/jcm14238417/s1, Table S1: Prisma 2020 Search Strategy Table, Table S2: Risk of bias assessment for the included RCTs. Table S3: PRISMA 2020 for abstracts checklist. Table S4 PRISMA 2020 Checklist.
