# Supplementary material for: Methylxanthines: The Major Impact of Caffeine in Clinical Practice in Patients Diagnosed with Apnea of Prematurity
Source: J Clin Med. 2025 Nov 27;14(23):8417. doi: 10.3390/jcm14238417 (PMC12693140; doi:10.3390/jcm14238417)
Supplement: Supplementary file 1 [file jcm-14-08417-s001.zip › jcm-3941318-supplementary.pdf]

Supplementary Appendix

Apnea of prematurity (AOP) represents one of the most common and clinically significant respiratory complications affecting preterm infants in neonatal intensive care units worldwide. Defined as cessation of breathing that lasts for more than 15 seconds and is accompanied by bradycardia or desaturation, this condition occurs in at least 85 percent of infants who are born at less than 34 weeks of gestation [1]. The prevalence increases inversely with gestational age, affecting virtually all infants born before 28 weeks gestation and presenting substantial challenges for clinical management and long-term neurodevelopmental outcomes.

The pathophysiology of apnea of prematurity is multifactorial, involving immature respiratory control mechanisms, unstable respiratory drive, and inadequate responses to hypoxia and hypercapnia [2–7]. The developing brainstem respiratory centers in preterm infants exhibit decreased sensitivity to carbon dioxide, altered sleep-wake cycling, and immature neural pathways that coordinate breathing patterns. These physiological limitations result in frequent episodes of central apnea, obstructive apnea, or mixed apnea patterns that can lead to significant hypoxemia, bradycardia, and potential long-term complications including impaired neurodevelopmental outcomes and increased risk of sudden infant death syndrome [1].

Methylxanthines have emerged as the cornerstone of pharmacological management for apnea of prematurity over the past several decades, confirmed by the landmark study Caffeine for Apnea of Prematurity (CAP) [8]. This class of medications includes three primary compounds used in clinical practice: caffeine citrate, theophylline, and aminophylline. These agents work through multiple mechanisms including adenosine receptor antagonism, phosphodiesterase inhibition, and enhancement of respiratory muscle contractility. The adenosine receptor antagonism is particularly important, as adenosine acts as a respiratory depressant in the central nervous system, and blocking these receptors enhances respiratory drive and reduces apneic episodes [5].

The clinical significance of effective apnea management extends far beyond the immediate respiratory concerns. Recurrent apneic episodes with associated hypoxemia and bradycardia can contribute to feeding difficulties, growth retardation, and most critically, adverse neurodevelopmental outcomes. Studies have demonstrated associations between frequent apneic episodes and increased risk of cerebral palsy, cognitive impairment, and behavioral problems in later childhood. Furthermore, the need for mechanical ventilation to manage severe apnea introduces additional risks including ventilator-associated lung injury, bronchopulmonary dysplasia, and increased healthcare costs [9].

1.1 Key definitions and concepts

Apnea of prematurity: Cessation of breathing lasting more than 15 seconds, often accompanied by bradycardia (heart rate <100 beats per minute) and/or oxygen desaturation (<85-90%), occurring in preterm infants without other identifiable causes [1].

Central apnea: Absence of respiratory effort due to lack of neural drive from the respiratory control centers in the brainstem.

Obstructive apnea: Cessation of airflow despite continued respiratory effort, typically due to upper airway obstruction.

Mixed apnea: Combination of central and obstructive components, representing the most common pattern in preterm infants.

Methylxanthines: A class of purine alkaloids that includes caffeine, theophylline, and aminophylline, characterized by their ability to antagonize adenosine receptors and inhibit phosphodiesterase enzymes [10].

Bronchopulmonary dysplasia (BPD): A chronic lung disease of prematurity characterized by impaired alveolar and vascular development, typically defined as oxygen dependency at 36 weeks postmenstrual age.

Postmenstrual Age (PMA): Gestational age at birth plus chronological age, used as a standardized measure of infant maturity in neonatal research.

1.2 Pathophysiology of apnea of prematurity

The pathophysiology of apnea of prematurity is multifactorial, involving immature respiratory control mechanisms, unstable respiratory drive, and inadequate responses to hypoxia and hypercapnia [2–5]. The developing brainstem respiratory centers in preterm infants exhibit decreased sensitivity to carbon dioxide, altered sleep-wake cycling, and immature neural pathways that coordinate breathing patterns. These physiological limitations result in frequent episodes of central apnea, obstructive apnea, or mixed apnea patterns that can lead to significant hypoxemia, bradycardia, and potential long-term complications including impaired neurodevelopmental outcomes and increased risk of sudden infant death syndrome [11].

1.2.1 Developmental respiratory control

The pathophysiology of apnea of prematurity is rooted in the immature development of respiratory control mechanisms in preterm infants. The respiratory control system involves complex interactions between central pattern generators in the medulla oblongata, chemoreceptors that detect changes in oxygen and carbon dioxide levels, and mechanoreceptors that respond to lung inflation and airway pressure changes. In preterm infants, these systems are incompletely developed, leading to unstable breathing patterns and increased susceptibility to apneic episodes [12].

The pre-Bötzinger complex, located in the rostral ventrolateral medulla, serves as the primary respiratory rhythm generator. In preterm infants, this region exhibits decreased neuronal connectivity and reduced sensitivity to hypercapnic stimulation compared to term infants. Additionally, the pontine respiratory centers, which modulate respiratory rhythm and coordinate breathing with other physiological functions, are immature in preterm infants, contributing to irregular breathing patterns and increased apnea susceptibility [13].

Central chemoreceptors, located in the medulla oblongata, normally respond to changes in cerebrospinal fluid pH and carbon dioxide levels by adjusting respiratory drive. In preterm infants, these chemoreceptors demonstrate blunted responses to hypercapnia, resulting in inadequate compensatory increases in ventilation during periods of carbon dioxide retention. This diminished chemosensitivity contributes to prolonged apneic episodes and delayed recovery from respiratory depression [14].

Peripheral chemoreceptors in the carotid and aortic bodies detect changes in arterial oxygen tension, carbon dioxide levels, and pH. While these receptors are functional in preterm infants, their integration with central respiratory control mechanisms is immature. The hypoxic ventilatory response in preterm infants is characterized by an initial brief increase in ventilation followed by sustained respiratory depression, contrasting with the sustained hyperventilation seen in mature individuals [15].

1.2.2 Sleep-wake state influences

Sleep-wake cycling significantly influences respiratory stability in preterm infants. During active (REM) sleep, which comprises a larger proportion of sleep time in preterm infants compared to term infants, respiratory control becomes more variable and dependent on behavioral state. The pontine mechanisms that normally maintain respiratory stability during REM sleep are immature in preterm infants, leading to increased frequency and severity of apneic episodes during this sleep state [16].

Quiet (non-REM) sleep, while generally associated with more stable breathing patterns, can also be problematic in preterm infants due to reduced respiratory drive and decreased responsiveness to chemical stimuli. The transitions between sleep states are particularly vulnerable periods for apnea occurrence, as the respiratory control mechanisms must adapt to changing neurological and physiological demands [17].

1.2.3 Upper airway factors

Upper airway anatomy and function contribute significantly to apnea pathophysiology in preterm infants. The relatively large head, short neck, and proportionally large tongue create anatomical predisposition to upper airway obstruction. Additionally, the laryngeal and pharyngeal muscles that maintain airway patency are immature and may not provide adequate support during inspiration, particularly during sleep when muscle tone is reduced [18].

The laryngeal chemoreflex, a protective mechanism that causes apnea, bradycardia, and laryngospasm in response to laryngeal stimulation, is hyperactive in preterm infants. This reflex can be triggered by gastroesophageal reflux, secretions, or mechanical stimulation, leading to prolonged apneic episodes that may require intervention [17,19].

1.3. Effects of methylxantines on the physiology of newborns

1.3.1 Respiratory system effects

Methylxanthines produce multiple beneficial effects on respiratory function in preterm infants. Central respiratory drive enhancement is the primary therapeutic effect, mediated through adenosine receptor antagonism in brainstem respiratory centers. This results in increased minute ventilation, improved responsiveness to hypercapnic stimulation, and reduced frequency and duration of central apneic episodes [20].

Diaphragmatic contractility improvement is another important effect of methylxanthine therapy. These compounds enhance the strength and endurance of respiratory muscles, particularly the diaphragm, through both central neural mechanisms and direct effects on muscle contractility. This is particularly beneficial in preterm infants who may have respiratory muscle fatigue contributing to apneic episodes [21].

Methylxanthines also improve lung mechanics by reducing airway resistance and increasing lung compliance. These effects are mediated through bronchodilation via adenosine receptor antagonism and phosphodiesterase inhibition. Additionally, methylxanthines may enhance surfactant production and function, contributing to improved lung compliance and gas exchange [22].

1.3.2 Cardiovascular effects

The cardiovascular effects of methylxanthines are complex and dose-dependent. At therapeutic doses used for apnea treatment, these compounds typically produce mild increases in heart rate and cardiac contractility through adenosine receptor antagonism and phosphodiesterase inhibition. These effects are generally well-tolerated in preterm infants and may be beneficial by improving cardiac output and tissue oxygen delivery [23].

Methylxanthines also affect vascular tone, producing mild vasoconstriction in some vascular beds while causing vasodilation in others. The net effect on blood pressure is typically minimal at therapeutic doses, though careful monitoring is required, particularly in infants with cardiovascular instability [24].

1.3.3 Central nervous system effects

Beyond their respiratory effects, methylxanthines produce important central nervous system effects that may contribute to their therapeutic benefits in preterm infants. These compounds enhance arousal and wakefulness through adenosine receptor antagonism in sleep-wake regulatory centers. This effect may be particularly beneficial in reducing apnea frequency during sleep states when respiratory control is most vulnerable [25].

Methylxanthines also enhance neurotransmitter release, particularly dopamine and norepinephrine, which may contribute to improved respiratory drive and arousal responses. Additionally, these compounds may have neuroprotective effects through antioxidant mechanisms and enhancement of neuronal survival pathways, though the clinical significance of these effects in preterm infants requires further investigation [26].

1.4 Comparative pharmacology of methylxanthines

1.4.1 Caffeine characteristics

Caffeine citrate has emerged as the preferred methylxanthine for apnea of prematurity treatment due to its favorable pharmacological profile. Caffeine has a longer half-life than theophylline (approximately 100 hours in preterm infants compared to 30 hours for theophylline), allowing for once-daily dosing and more stable plasma concentrations. The therapeutic window for caffeine is wider than for theophylline, with less risk of toxicity at effective doses [1,27].

Caffeine demonstrates excellent bioavailability via both intravenous and oral routes, with minimal first-pass metabolism in preterm infants due to immature hepatic enzyme systems. The drug is primarily eliminated through renal excretion in preterm infants, with minimal hepatic metabolism until several months of age. This pharmacokinetic profile contributes to predictable dosing and reduced risk of drug interactions [1].

1.4.2 Theophylline characteristics

Theophylline was historically the first methylxanthine used for apnea of prematurity but has largely been replaced by caffeine in clinical practice. Theophylline has a shorter half-life than caffeine, requiring more frequent dosing (typically every 8-12 hours) and more intensive monitoring of plasma concentrations. The therapeutic window for theophylline is narrower than for caffeine, with increased risk of toxicity at supratherapeutic levels [28].

Theophylline metabolism is more complex than caffeine, involving multiple hepatic cytochrome P450 enzymes that are immature in preterm infants but develop rapidly during the first months of life. This leads to significant changes in clearance and dosing requirements as infants mature, necessitating frequent dose adjustments and plasma level monitoring [29].

1.4.3 Aminophylline characteristics

Aminophylline is a compound consisting of theophylline complexed with ethylenediamine to improve solubility for intravenous administration. The pharmacological effects of aminophylline are essentially identical to theophylline, as the ethylenediamine component has no significant pharmacological activity. Aminophylline is typically used when intravenous administration is required and oral theophylline is not feasible [30].

The dosing of aminophylline must account for the fact that it contains approximately 80% theophylline by weight, requiring dose calculations based on theophylline equivalents. Like theophylline, aminophylline requires frequent monitoring of plasma concentrations and dose adjustments based on individual pharmacokinetic variability [31].

1.5 Historical perspective and evolution of practice

1.5.1 Early recognition and treatment

The recognition of apnea of prematurity as a distinct clinical entity emerged in the 1960s as neonatal intensive care capabilities expanded and survival of very preterm infants increased. Early management approaches focused primarily on physical stimulation and mechanical ventilation, with limited understanding of the underlying pathophysiology or pharmacological treatment options [32].

The first use of methylxanthines for apnea of prematurity was reported in the late 1970s, initially with theophylline based on its known respiratory stimulant effects in adults with chronic obstructive pulmonary disease. Early studies demonstrated significant efficacy in reducing apnea frequency and facilitating weaning from mechanical ventilation, leading to rapid adoption of methylxanthine therapy in neonatal intensive care units [33].

1.5.2 Evolution of evidence base

The evidence base for methylxanthine therapy in apnea of prematurity has evolved significantly over the past four decades. Early studies were typically small, single-center trials with limited follow-up and variable outcome measures. The recognition of the need for larger, multicenter trials led to the development of collaborative research networks and standardized outcome measures[1].

The evolution from using methylxanthines solely for apnea to recognizing their broader respiratory benefits began with studies like Greenough et al. (1985), which demonstrated theophylline's efficacy in facilitating ventilator weaning [34]. This expanded indication represented a paradigm shift in understanding methylxanthine actions, moving beyond simple respiratory stimulation to recognition of effects on lung mechanics and respiratory muscle function. The study's finding that benefits were limited in extremely premature infants (<27 weeks) presaged later work identifying patient subgroups who derive maximal benefit from methylxanthine therapy.

The landmark Caffeine for Apnea of Prematurity (CAP) trial, published in 2006, represented a watershed moment in the field by providing definitive evidence for caffeine’s efficacy and safety in a large, multicenter, randomized controlled trial [8]. This trial not only confirmed caffeine’s effectiveness for apnea treatment but also demonstrated unexpected benefits including reduced bronchopulmonary dysplasia and improved neurodevelopmental outcomes [35].

1.5.3 Current practice patterns

Contemporary practice patterns for methylxanthine therapy in apnea of prematurity have been significantly influenced by the CAP trial results and subsequent follow-up studies. Caffeine citrate has become the preferred first-line therapy for most neonatal intensive care units, with standardized dosing protocols and monitoring guidelines widely adopted [43].

Current practice emphasizes early initiation of caffeine therapy, often within the first few days of life for very preterm infants, based on evidence suggesting that early treatment may provide additional benefits beyond apnea reduction. The duration of therapy typically continues until infants reach 34-37 weeks postmenstrual age or demonstrate consistent absence of apneic episodes for several days to weeks [40].

Supplementary table S1. Prisma 2020 Search Strategy Table.

Database	Date / Coverage	Search Terms and Boolean Combinations	Limits / Filters
**PubMed (MEDLINE)**	1970 – Jan 2025	**Population:** (“preterm infant” OR “premature infant” OR “neonate” OR “very low birth weight” OR “extremely low birth weight”) **Condition:** (“apnea of prematurity” OR “apnoea of prematurity” OR “neonatal apnea” OR “infant apnea”) **Intervention:** (“methylxanthine*” OR “caffeine” OR “theophylline” OR “aminophylline” OR “xanthine derivative*”) **Study Design:** (“randomized controlled trial” OR “controlled clinical trial” OR “prospective study” OR “retrospective study” OR “case-control”) **Final Boolean string:** `(("preterm infant" OR "premature infant" OR "neonate" OR "very low birth weight" OR "extremely low birth weight") AND ("apnea of prematurity" OR "apnoea of prematurity" OR "neonatal apnea" OR "infant apnea") AND ("methylxanthine*" OR "caffeine" OR "theophylline" OR "aminophylline" OR "xanthine derivative*") AND ("randomized controlled trial" OR "controlled clinical trial" OR "prospective" OR "retrospective" OR "case-control"))`	No language restriction; humans; 0–120 days postnatal age.
**Embase**	1970 – Jan 2025	(`premature infant`/exp OR `very low birth weight` OR `neonate`) AND (`apnea of prematurity`/exp OR `neonatal apnea` OR `infant apnea`) AND (`methylxanthine derivative`/exp OR caffeine/exp OR theophylline/exp OR aminophylline/exp) AND (`randomized controlled trial`/exp OR `cohort analysis`/exp OR `case control study`/exp)	Human studies; newborn < 37 weeks gestation.
**Web of Science (Core Collection)**	1970 – Jan 2025	TS = ((“apnea of prematurity” OR “neonatal apnea”) AND (“caffeine” OR “theophylline” OR “aminophylline” OR “methylxanthine*”) AND (“preterm infant” OR “premature infant” OR “low birth weight” OR “neonate”) AND (“randomized” OR “controlled” OR “clinical trial” OR “cohort” OR “case-control”))	Document types: Article or Clinical Trial; Language: All.

Supplementary table S2. Risk of bias assessment for the included RCTs.

**Unique ID**	1	**Study ID**	Aranda et al (1983)	**Assessor**	
**Ref or Label**	Aranda et al (1983)	**Aim**	assignment to intervention (the 'intention-to-treat' effect)		

**Experimental**	Theophylline (4 mg/kg 6-hourly, increased to 6 mg/kg if no response)	**Comparator**	Placebo (same base as theophylline)	**Source**	Journal article(s)
**Outcome**	Cessation of apneic episodes (defined as decrease within 6-12 hours with no further episodes requiring intervention over 48 hours)	**Results**	Success: 10/14 (71%) theophylline vs 0/11 (0%) placebo; Chi-square = 10.29, p<0.01	**Weight**	1

Domain	Signalling question	Response	Comments
**Bias arising from the randomization process**	1.1 Was the allocation sequence random?	PY	"Lucky dip" method from 30 slips marked A-F. While not a computerized random sequence, this represents a random selection process.<br><br>The key to the content of the bottles was known only to the pharmacist. Bottles marked A-F, central allocation by pharmacist
	1.2 Was the allocation sequence concealed until participants were enrolled and assigned to interventions?	Y	
	1.3 Did baseline differences between intervention groups suggest a problem with the randomization process?	N	Table 1 shows no statistical differences in birth weight, gestational age, or age at commencement between groups.
	**Risk of bias judgement**	**Low**	Adequate randomization with 'lucky dip' method and strong allocation concealment via pharmacy-controlled labeling. Baseline characteristics well-balanced between groups."
**Bias due to deviations from intended interventions**	2.1.Were participants aware of their assigned intervention during the trial?	N	Double-blind design with identical placebo.<br><br>Double blind trial - neither staff nor investigators knew allocation
	2.2.Were carers and people delivering the interventions aware of participants' assigned intervention during the trial?	N	
	2.3. If Y/PY/NI to 2.1 or 2.2: Were there deviations from the intended intervention that arose because of the experimental context?	NA	
	2.4 If Y/PY to 2.3: Were these deviations likely to have affected the outcome?	NA	
	2.5. If Y/PY/NI to 2.4: Were these deviations from intended intervention balanced between groups?	NA	
	2.6 Was an appropriate analysis used to estimate the effect of assignment to intervention?	PN	4 patients excluded post-randomization (3 placebo, 1 theophylline) due to deaths from fatal conditions.

			This violates ITT principles.
	2.7 If N/PN/NI to 2.6: Was there potential for a substantial impact (on the result) of the failure to analyse participants in the group to which they were randomized?	PN	Small number excluded (13.8% total), but unbalanced (21% of placebo vs 7% of theophylline). However, exclusions were due to clearly unrelated fatal conditions (IVH, NEC), not treatment effects.
	**Risk of bias judgement**	**Some concerns**	While double-blinding was maintained, 4 patients were excluded from analysis after randomization (3/14 placebo, 1/15 theophylline) due to deaths from conditions stated to be unrelated to apnea. This is a modified ITT analysis. The impact is likely limited as exclusion reasons appear unrelated to treatment, but violates strict ITT principles.
**Bias due to missing outcome data**	3.1 Were data for this outcome available for all, or nearly all, participants randomized?	PN	4/29 (13.8%) excluded: 3/14 placebo (21%), 1/15 theophylline (7%).
	3.2 If N/PN/NI to 3.1: Is there evidence that result was not biased by missing outcome data?	PY	Reasons for exclusion documented and appear outcome-independent: Deaths from IVH (n=2) and NEC (n=2) - conditions unrelated to apnea treatment. These were fatal conditions making outcome assessment impossible.
	3.3 If N/PN to 3.2: Could missingness in the outcome depend on its true value?	NA	
	3.4 If Y/PY/NI to 3.3: Is it likely that missingness in the outcome depended on its true value?	NA	
	**Risk of bias judgement**	**Low**	Although 13.8% of participants were excluded, the reasons (death from intraventricular hemorrhage and necrotizing enterocolitis) were clearly documented, clinically appropriate, and unrelated to the apnea outcome. These conditions made outcome assessment impossible and were not plausibly related to treatment effect.
**Bias in measurement of the outcome**	4.1 Was the method of measuring the outcome inappropriate?	N	Apnea measured by dual monitoring (apnoea mattress + cardiac monitor) with nurse observation. Appropriate for this outcome.
	4.2 Could measurement or ascertainment of the outcome have differed between intervention groups?	N	All infants monitored identically with same equipment and observation protocols.

	4.3 Were outcome assessors aware of the intervention received by study participants?	N	Double-blind trial - nurses and clinicians unaware of treatment allocation.
	4.4 If Y/PY/NI to 4.3: Could assessment of the outcome have been influenced by knowledge of intervention received?	NA	
	4.5 If Y/PY/NI to 4.4: Is it likely that assessment of the outcome was influenced by knowledge of intervention received?	NA	
	**Risk of bias judgement**	**Low**	Double-blind design with objective monitoring (apnoea mattress and cardiac monitor) plus experienced nurse observation. Measurement methods consistent across groups. Authors acknowledged potential for underestimation of apnea but noted this would apply equally to both groups.
**Bias in selection of the reported result**	5.1 Were the data that produced this result analysed in accordance with a pre-specified analysis plan that was finalized before unblinded outcome data were available for analysis?	NI	No protocol or trial registration mentioned (pre-dates trial registration era).
	5.2 ... multiple eligible outcome measurements (e.g. scales, definitions, time points) within the outcome domain?	PN	Primary outcome clearly defined (cessation of apnea within specific timeframe). No evidence of multiple outcome measurements.
	5.3 ... multiple eligible analyses of the data?	PN	Single analysis approach described. Authors note criteria for success were extremely rigid.
	**Risk of bias judgement**	**Some concerns**	No pre-specified analysis plan available (study conducted in 1981, predating trial registration requirements). However, outcome definition appears straightforward and consistently applied. Trial was stopped early as 'increasing number of reports were appearing in the literature supporting theophylline' - suggests interim analysis.
**Overall bias**	**Risk of bias judgement**	**Some concerns**	The study is at low risk of bias for randomization, missing outcome data, and outcome measurement (strong double-blind design). However, there are some concerns due to: (1) Post-randomization exclusions of 4 patients, violating strict ITT principles, though exclusions were for documented, outcome-independent reasons; (2) No pre-specified analysis plan available (pre-dates registration era); (3) Trial stopped early based on emerging literature. Overall, this is a well-

			conducted double-blind RCT with methodological rigor appropriate for its era.

**Unique ID**	2	**Study ID**	Gupta et al (1981)	**Assessor**	
**Ref or Label**	Gupta et al (1981)	**Aim**	assignment to intervention (the 'intention-to-treat' effect)		
**Experimental**	Theophylline 4 mg/kg q6h via nasogastric tube; increased to 6 mg/kg q6h if no clinical response within the predefined window	**Comparator**	Matching placebo (vehicle) via nasogastric tube (identical schedule)	**Source**	Journal article(s)
**Outcome**	Reduction in number of apneic episodes within 6–12 h of starting therapy; sustained absence of further episodes assessed over the next 48 h	**Results**	Response within 6–12 h: 10/15 in the theophylline arm showed a reduction in apnea frequency	**Weight**	1

**Domain**	**Signalling question**	**Response**	**Comments**
**Bias arising from the randomization process**	1.1 Was the allocation sequence random?	Y	Authors describe a double-blind randomized design using pharmacy-coded study bottles with identical placebo.
	1.2 Was the allocation sequence concealed until participants were enrolled and assigned to interventions?	Y	
	1.3 Did baseline differences between intervention groups suggest a problem with the randomization process?	N	Baseline infant characteristics were similar between groups.
	**Risk of bias judgement**	**Low**	Authors describe a double-blind randomized design using pharmacy-coded study bottles with identical placebo. Baseline infant characteristics were similar between groups, indicating effective randomization and concealment.

**Bias due to deviations from intended interventions**	2.1.Were participants aware of their assigned intervention during the trial?	N	Newborns cannot be aware.<br><br>Carers and people delivering the interventions were not aware of participants’ assigned intervention
	2.2.Were carers and people delivering the interventions aware of participants' assigned intervention during the trial?	N	
	2.3. If Y/PY/NI to 2.1 or 2.2: Were there deviations from the intended intervention that arose because of the experimental context?	NA	
	2.4 If Y/PY to 2.3: Were these deviations likely to have affected the outcome?	NA	
	2.5. If Y/PY/NI to 2.4: Were these deviations from intended intervention balanced between groups?	NA	
	2.6 Was an appropriate analysis used to estimate the effect of assignment to intervention?	PY	The trial was double-blind with identical dosing and administration in both arms.
	2.7 If N/PN/NI to 2.6: Was there potential for a substantial impact (on the result) of the failure to analyse participants in the group to which they were randomized?	NA	
	**Risk of bias judgement**	**Low**	The trial was double-blind with identical dosing and administration in both arms. No protocol deviations related to awareness of assignment were reported.
**Bias due to missing outcome data**	3.1 Were data for this outcome available for all, or nearly all, participants randomized?	PN	Infants excluded due to early death.
	3.2 If N/PN/NI to 3.1: Is there evidence that result was not biased by missing outcome data?	N	Is unclear if the deaths of infants impacted the study.
	3.3 If N/PN to 3.2: Could missingness in the outcome depend on its true value?	PY	In small sample size analysis per-protocol can induce bias.
	3.4 If Y/PY/NI to 3.3: Is it likely that missingness in the outcome depended on its true value?	PY	
	**Risk of bias judgement**	**Some concerns**	A few infants were excluded post-randomization due to early deaths or comorbid conditions. Analysis appears per-protocol rather than ITT, which could bias results given the small sample size.
**Bias in measurement of the outcome**	4.1 Was the method of measuring the outcome inappropriate?	N	Apnea measured by bedside cardiorespiratory monitors and clinical observation; while blinded, equipment precision and lack of independent

			adjudication could introduce modest measurement bias.
	4.2 Could measurement or ascertainment of the outcome have differed between intervention groups?	PN	
	4.3 Were outcome assessors aware of the intervention received by study participants?	N	
	4.4 If Y/PY/NI to 4.3: Could assessment of the outcome have been influenced by knowledge of intervention received?	NA	
	4.5 If Y/PY/NI to 4.4: Is it likely that assessment of the outcome was influenced by knowledge of intervention received?	NA	
	**Risk of bias judgement**	**Some concerns**	Apnea measured by bedside cardiorespiratory monitors and clinical observation; while blinded, equipment precision and lack of independent adjudication could introduce modest measurement bias.
**Bias in selection of the reported result**	5.1 Were the data that produced this result analysed in accordance with a pre-specified analysis plan that was finalized before unblinded outcome data were available for analysis?	PY	Primary success criteria (≥ 50 % apnea reduction within 6–12 h and sustained absence over 48 h) were explicitly defined in methods and reported as planned.
	5.2 ... multiple eligible outcome measurements (e.g. scales, definitions, time points) within the outcome domain?	NI	
	5.3 ... multiple eligible analyses of the data?	NI	
	**Risk of bias judgement**	**Low**	Primary success criteria (≥ 50 % apnea reduction within 6–12 h and sustained absence over 48 h) were explicitly defined in methods and reported as planned.
**Overall bias**	**Risk of bias judgement**	**Some concerns**	Randomization and blinding were robust; the small sample size and per-protocol analysis raise some uncertainty regarding missing data and outcome measurement reliability.

**Unique ID**	3	**Study ID**	Murat et al (1981)	**Assessor**	
**Ref or Label**	Murat et al (1981)	**Aim**	assignment to intervention (the 'intention-to-treat' effect)		
**Experimental**	Caffeine sodium citrate (20 mg/kg IM loading dose, then 5 mg/kg daily oral maintenance)	**Comparator**	CControl (no caffeine, tactile stimulation/facemask ventilation only)	**Source**	Journal article(s)
**Outcome**	Apnea index (number of apneic attacks per 100 minutes) - both severe and mild apnea	**Results**	Day 5 severe apnea: 0.24±0.08 (treated) vs 0.74±0.17 (control), p<0.01; Mild apnea: 1.24±0.25 vs 1.78±0.34, p<0.001	**Weight**	1

Domain	Signalling question	Response	Comments
**Bias arising from the randomization process**	1.1 Was the allocation sequence random?	NI	States "randomly assigned" but provides NO details on method of randomization - no computer generation, no table of random numbers, no sealed envelopes, nothing.<br><br>No information provided about allocation concealment methods.
	1.2 Was the allocation sequence concealed until participants were enrolled and assigned to interventions?	NI	
	1.3 Did baseline differences between intervention groups suggest a problem with the randomization process?	N	Table shows no significant differences between groups: gestational age (30.1±0.6 vs 29.8±0.5 weeks), birth weight (1247±101 vs 1411±71 gm), postnatal age (13.2±2.3 vs 16.1±3.3 days). Day 0 apnea indices also similar between groups.
	**Risk of bias judgement**	**Some concerns**	Study states infants were 'randomly assigned' but provides no details on randomization method or allocation concealment. However, baseline characteristics are well-balanced, suggesting randomization may have been adequate. Given the era (1981) and lack of reporting standards, this represents unclear rather than inadequate methods.

**Bias due to deviations from intended interventions**	2.1.Were participants aware of their assigned intervention during the trial?	Y	Authors explicitly state: "a double-blind trial was not believed necessary" - this is an OPEN-LABEL study.<br><br>Open-label design - nurses and physicians aware of treatment allocation.
	2.2.Were carers and people delivering the interventions aware of participants' assigned intervention during the trial?	Y	
	2.3. If Y/PY/NI to 2.1 or 2.2: Were there deviations from the intended intervention that arose because of the experimental context?	Y	In control group, 6/9 infants (67%) received rescue therapy (2 got IMV, 4 got caffeine) because "withholding additional treatment was believed to be unethical." These were protocol deviations arising from trial context.
	2.4 If Y/PY to 2.3: Were these deviations likely to have affected the outcome?	Y	Rescue interventions (IMV and caffeine) directly affect apnea outcomes.
	2.5. If Y/PY/NI to 2.4: Were these deviations from intended intervention balanced between groups?	N	Completely unbalanced: 6/9 control infants received rescue therapy vs 0/9 in caffeine group
	2.6 Was an appropriate analysis used to estimate the effect of assignment to intervention?	N	Authors excluded control group failures from analysis after they received rescue therapy. Day 5 analysis: 9 treated vs only 6 controls (3 excluded). Day 15: 9 treated vs only 3 controls (6 excluded).
	2.7 If N/PN/NI to 2.6: Was there potential for a substantial impact (on the result) of the failure to analyse participants in the group to which they were randomized?	Y	Excluding 67% of control group who failed treatment has major impact on results.
	**Risk of bias judgement**	**High**	Critical flaws: (1) Open-label design with unblinded assessment; (2) 6/9 control infants (67%) required rescue therapy and were subsequently EXCLUDED from analysis; (3) Complete lack of ITT analysis. The exclusion of treatment failures from the control group fundamentally undermines the validity of the comparison. This is effectively a 'per-protocol' analysis that excludes precisely those patients who demonstrate lack of efficacy in the control group.
**Bias due to missing outcome data**	3.1 Were data for this outcome available for all, or nearly all, participants randomized?	N	Day 5: 9/9 treated (100%) vs 6/9 control (67%);<br>Day 15: 9/9 treated vs 3/9 control (33%)
	3.2 If N/PN/NI to 3.1: Is there evidence that result was not biased by missing outcome data?	N	No sensitivity analyses performed. Authors simply excluded failures.
	3.3 If N/PN to 3.2: Could missingness in the outcome depend on its true value?	Y	Patients were excluded BECAUSE they had treatment failure (severe, persistent apnea

	3.4 If Y/PY/NI to 3.3: Is it likely that missingness in the outcome depended on its true value?	Y	requiring rescue therapy).<br><br>The missingness IS the outcome - exclusions were due to severe apnea requiring intervention.
	**Risk of bias judgement**	**High**	By day 15, 67% of the control group was excluded due to treatment failure requiring rescue therapy. The missingness is completely dependent on the outcome - patients with the worst apnea were systematically removed from the control group. This creates severe bias favoring the treatment group. This is not 'missing data' in the traditional sense but rather exclusion of treatment failures.
**Bias in measurement of the outcome**	4.1 Was the method of measuring the outcome inappropriate?	N	24-hour cardiorespirographic recordings (Hewlett-Packard) with objective criteria: severe apnea = cessation >10 sec with HR <80/min for >30 sec or <60/min for >15 sec; mild apnea = cessation >10 sec with HR 60-100/min for <15 sec.
	4.2 Could measurement or ascertainment of the outcome have differed between intervention groups?	PN	Same recording equipment used for all patients. However, interpretation of recordings could differ.
	4.3 Were outcome assessors aware of the intervention received by study participants?	Y	Open-label study - no blinding of outcome assessors who had to interpret the cardiorespirographic recordings.
	4.4 If Y/PY/NI to 4.3: Could assessment of the outcome have been influenced by knowledge of intervention received?	Y	Although recordings are objective, someone must interpret them and count apneic episodes. Authors noted in other studies "counting attacks depended on nurses" - suggesting some subjectivity.<br><br>In an open-label study with strong beliefs about efficacy, interpretation of borderline events (e.g., exactly 10 seconds? exactly 60 bpm?) could be influenced by knowledge of treatment.
	4.5 If Y/PY/NI to 4.4: Is it likely that assessment of the outcome was influenced by knowledge of intervention received?	PY	
	**Risk of bias judgement**	**High**	While the recording equipment was objective, the study was open-label and interpreters of the recordings knew which group patients were in. Given that apnea definition involves threshold judgments (duration, heart rate), knowledge of treatment allocation could influence classification of borderline events. Authors acknowledged this limitation by stating they used recordings to improve objectivity, but never addressed the lack of

			blinded interpretation.
**Bias in selection of the reported result**	5.1 Were the data that produced this result analysed in accordance with a pre-specified analysis plan that was finalized before unblinded outcome data were available for analysis?	NI	No protocol available (1981, pre-registration era). However, outcomes appear consistently defined.
	5.2 ... multiple eligible outcome measurements (e.g. scales, definitions, time points) within the outcome domain?	PN	Apnea index clearly defined with severe/mild categories. Multiple time points (day 1, 5, 15) all reported.
	5.3 ... multiple eligible analyses of the data?	PN	Single analysis approach reported throughout.
	**Risk of bias judgement**	**Some concerns**	
**Overall bias**	**Risk of bias judgement**	**High**	This study has critical methodological flaws that severely compromise its validity:<br><br>Open-label design with no blinding of participants, personnel, or outcome assessors<br><br>Massive exclusion of treatment failures from the control group: 67% of control patients were excluded from analysis by day 15 because they required rescue therapy for severe, persistent apnea<br><br>Complete absence of ITT analysis: The very patients who demonstrate treatment necessity in the control group were systematically removed from the comparison<br><br>Missingness completely dependent on outcome: Exclusions occurred because of treatment failure (severe apnea)<br><br>Subjective outcome assessment in an unblinded context with potential for detection bias

			While the study provides important early evidence for caffeine efficacy, the exclusion of control group failures who required rescue therapy fundamentally undermines the comparison. This is analogous to analyzing only patients who did well in the control group while keeping all patients in the treatment group.

**Unique ID**	4	**Study ID**	Greenough et al (1985)	**Assessor**	
**Ref or Label**	Greenough et al (1985)	**Aim**	assignment to intervention (the 'intention-to-treat' effect)		
**Experimental**	Oral theophylline (5 mg/kg loading dose, then 1 ml/kg/day divided in 4 doses, adjusted to maintain therapeutic levels 5-13 mg/L)	**Comparator**	Placebo (vehicle only - identical appearance)	**Source**	Journal article(s)
**Outcome**	(1) Lung compliance at 6 hours; (2) Duration of mechanical ventilation from trial entry	**Results**	Compliance at 6h: 0.95±0.42 (theophylline) vs 0.67±0.28 ml/cm H_2O (placebo), $p<0.05$. Time to extubation: $p<0.01$	**Weight**	1

**Domain**	**Signalling question**	**Response**	**Comments**
**Bias arising from the randomization process**	1.1 Was the allocation sequence random?	PY	"50 bottles were randomised by the pharmacy" - pharmacy-controlled randomization. Method not fully described but pharmacy randomization is typically appropriate.<br><br>Excellent concealment: "These had been made up by the hospital pharmacy...Both formed a clear solution and were indistinguishable. 50 bottles were randomised by the pharmacy who kept the
	1.2 Was the allocation sequence concealed until participants were enrolled and assigned to interventions?	Y	

			code which was not released until the end of the trial." Central pharmacy allocation with concealed code.
	1.3 Did baseline differences between intervention groups suggest a problem with the randomization process?	N	Table I shows well-balanced groups: gestational age (29.8±2.4 vs 29.5±2.3 weeks), birthweight (1448±503 vs 1423±492g), compliance at entry (0.67±0.35 vs 0.63±0.26). Postnatal age slightly younger in theophylline group (91.6±12.9 vs 111.0±75.6h) but "did not reach statistical significance." Table II shows similar complication rates.
	**Risk of bias judgement**	**Low**	Strong randomization with pharmacy-controlled allocation concealment. Code kept sealed until trial completion. Baseline characteristics well-balanced between groups. This represents optimal randomization methodology for the 1980s era
**Bias due to deviations from intended interventions**	2.1.Were participants aware of their assigned intervention during the trial?	N	Double-blind design with identical placebo: "Both formed a clear solution and were indistinguishable"<br><br>"The prescriber (A.G.) and the clinical team were unaware of which treatment the infant received...Both the clinicians and A.G. were kept 'blind'."
	2.2.Were carers and people delivering the interventions aware of participants' assigned intervention during the trial?	N	
	2.3. If Y/PY/NI to 2.1 or 2.2: Were there deviations from the intended intervention that arose because of the experimental context?	NA	
	2.4 If Y/PY to 2.3: Were these deviations likely to have affected the outcome?	NA	
	2.5. If Y/PY/NI to 2.4: Were these deviations from intended intervention balanced between groups?	NA	
	2.6 Was an appropriate analysis used to estimate the effect of assignment to intervention?	PN	Post-randomization exclusions: "Two infants were withdrawn from the trial within 4 h of entry...consequently their results were not included in the analysis." Both received active theophylline. This is a modified ITT excluding early withdrawals.
	2.7 If N/PN/NI to 2.6: Was there potential for a substantial impact (on the result) of the failure to analyse participants in the group to which they were randomized?	PN	Only 2/40 (5%) excluded, both from theophylline group due to documented adverse effects (tachycardia >200 bpm, agitation requiring paralysis) within 4 hours. Small numbers, clinically

			justified exclusions, unlikely to substantially bias results toward theophylline.
	**Risk of bias judgement**	**Low**	Excellent double-blind design maintained throughout. Two infants (both theophylline) withdrawn within 4 hours due to documented adverse effects and excluded from analysis. While this violates strict ITT, the small number (5%), early timing, documented safety reasons, and direction of bias (removing theophylline failures) make substantial bias unlikely. If anything, excluding these two might bias AGAINST theophylline.
**Bias due to missing outcome data**	3.1 Were data for this outcome available for all, or nearly all, participants randomized?	PY	38/40 (95%) included in analysis. 2 excluded very early (within 4h)
	3.2 If N/PN/NI to 3.1: Is there evidence that result was not biased by missing outcome data?	NA	
	3.3 If N/PN to 3.2: Could missingness in the outcome depend on its true value?	NA	
	3.4 If Y/PY/NI to 3.3: Is it likely that missingness in the outcome depended on its true value?	NA	
	**Risk of bias judgement**	**Low**	Only 5% missing data (2/40). Both exclusions were from the theophylline group due to documented adverse effects within 4 hours. This represents transparent, clinically appropriate safety-based exclusions. Direction of any potential bias is conservative (against theophylline). Authors noted 'During the course of the study there were no other serious side-effects' and included the one death (control group) in analyses.
**Bias in measurement of the outcome**	4.1 Was the method of measuring the outcome inappropriate?	N	Objective measurements: Static compliance using "equipment described previously" with peak pressure and volume change. Dynamic compliance post-extubation using pneumotachograph and oesophageal balloon with occlusion test validation. Duration of ventilation objectively recorded.
	4.2 Could measurement or ascertainment of the outcome have differed between intervention groups?	N	"Respiratory function was measured in all infants...using equipment described previously." Same equipment and protocols for both groups.

			"AG present to make the respiratory measurements" - single blinded investigator.
	4.3 Were outcome assessors aware of the intervention received by study participants?	N	"Both the clinicians and A.G. were kept 'blind'." A.G. performed all respiratory measurements while blinded. Clinicians managing ventilator weaning were also blinded.
	4.4 If Y/PY/NI to 4.3: Could assessment of the outcome have been influenced by knowledge of intervention received?	NA	
	4.5 If Y/PY/NI to 4.4: Is it likely that assessment of the outcome was influenced by knowledge of intervention received?	NA	
	**Risk of bias judgement**	**Low**	Excellent blinding of outcome assessment. Single investigator (A.G.) made all respiratory function measurements while blinded to treatment allocation. Objective compliance measurements using validated equipment. Clinicians making weaning decisions were also blinded, preventing detection bias in duration of ventilation outcome. This represents optimal outcome assessment methodology.
**Bias in selection of the reported result**	5.1 Were the data that produced this result analysed in accordance with a pre-specified analysis plan that was finalized before unblinded outcome data were available for analysis?	Y	Strong pre-specification: "The study was designed to detect a difference in compliance of 0.3 ml/cm H_2O...40 patients would be required...Consequently the randomisation code was not broken until 40 infants had been entered into the trial." Sample size calculated in advance, code kept sealed until target enrollment reached.
	5.2 ... multiple eligible outcome measurements (e.g. scales, definitions, time points) within the outcome domain?	N	Two co-primary outcomes clearly stated: compliance at 6 hours and duration of ventilation. Compliance measured at specified time points (entry, 30 min, 1, 2, 3, 6 hours). All time points reported in text.
	5.3 ... multiple eligible analyses of the data?	N	Pre-specified analysis approach. Authors also conducted sensitivity analysis: "Re-analysing the data, including only the surviving infants, still demonstrates that those infants receiving theophylline required significantly less ventilation (P<0.01)" and subgroup analysis by gestational age, appropriately labeled as exploratory.

	**Risk of bias judgement**	**Low**	Excellent pre-specification of study design, outcomes, and analysis plan. Sample size calculated prospectively. Randomization code kept sealed until target enrollment (40 patients) reached. Primary outcomes clearly defined. Sensitivity analyses appropriately labeled. This represents best-practice for 1985 and exceeds standards of many contemporary trials.
**Overall bias**	**Risk of bias judgement**	**Low**	This is a well-designed, well-conducted, double-blind randomized controlled trial with minimal risk of bias across all domains:<br><br>Strengths:<br><br>Optimal randomization: Pharmacy-controlled with central allocation concealment and sealed code until study completion<br><br>Rigorous double-blinding: Both participants, clinical team, and outcome assessors blinded using identical placebo<br><br>Objective outcomes: Compliance measured using validated equipment by single blinded investigator<br><br>Pre-specified design: Sample size calculated prospectively, code not broken until target enrollment reached<br><br>Transparent reporting: Early withdrawals clearly reported with reasons (both from theophylline group)<br><br>Minimal missing data: 95% retention (38/40 patients)<br><br>Well-balanced groups: No baseline differences in key prognostic factors

			Appropriate analysis: Sensitivity analyses conducted, subgroup analyses appropriately labeled<br><br>Minor Limitations:<br><br>Post-randomization exclusion of 2 patients (both theophylline) for safety reasons - violates strict ITT but:<br><br>Only 5% of sample<br><br>Both excluded within 4 hours for documented adverse effects<br><br>Direction of potential bias is conservative (AGAINST theophylline)<br><br>Transparently reported<br><br>Sensitivity analysis confirmed findings<br><br>Comparison to Other Studies:<br><br>Much stronger than: Aranda 1977 (no control group), Murat 1981 (open-label, 67% control exclusions)

			Similar quality to: Gupta 1981 (also double-blind RCT)<br><br>Exceptional for 1985: Pre-registration not required then, but authors effectively achieved this by keeping code sealed<br><br>This study represents gold-standard methodology for its era and would meet contemporary standards for clinical trial conduct.

**Unique ID**	5	**Study ID**	Bairam et al (1987)	**Assessor**	
**Ref or Label**	Bairam et al (1987)	**Aim**	assignment to intervention (the 'intention-to-treat' effect)		
**Experimental**	Theophylline (6 mg/kg loading, 2 mg/kg q12h maintenance)	**Comparator**	Caffeine (10 mg/kg loading, 1.25 mg/kg q12h maintenance)	**Source**	Journal article(s)
**Outcome**	Reduction in apnea/bradycardia episodes	**Results**	Effect of assignment to intervention (intention-to-treat)	**Weight**	1

**Domain**	**Signalling question**	**Response**	**Comments**
**Bias arising from the randomization process**	1.1 Was the allocation sequence random?	Y	The infants were "randomly assigned" to two groups; sample balance (10 vs 10) suggests true randomization.<br><br>Not explicitly described, but drugs were identical and prepared by a biochemist unaware of treatment, implying concealment.
	1.2 Was the allocation sequence concealed until participants were enrolled and assigned to interventions?	PY	

	1.3 Did baseline differences between intervention groups suggest a problem with the randomization process?	N	Groups had comparable birth weights, gestational and postnatal ages.
	**Risk of bias judgement**	**Low**	Randomization and concealment appear adequate; no imbalances observed.
**Bias due to deviations from intended interventions**	2.1.Were participants aware of their assigned intervention during the trial?	N	Both drugs had identical appearance and were injected IV.<br><br>Dosing adjustments were made by a biochemist unaware of the drug
	2.2.Were carers and people delivering the interventions aware of participants' assigned intervention during the trial?	N	
	2.3. If Y/PY/NI to 2.1 or 2.2: Were there deviations from the intended intervention that arose because of the experimental context?	NA	
	2.4 If Y/PY to 2.3: Were these deviations likely to have affected the outcome?	NA	
	2.5. If Y/PY/NI to 2.4: Were these deviations from intended intervention balanced between groups?	NA	
	2.6 Was an appropriate analysis used to estimate the effect of assignment to intervention?	PY	All infants appear included; random assignment and group comparisons reported without exclusions.
	2.7 If N/PN/NI to 2.6: Was there potential for a substantial impact (on the result) of the failure to analyse participants in the group to which they were randomized?	NA	
	**Risk of bias judgement**	**Low**	Double-blind design; interventions identical; no deviations likely.
**Bias due to missing outcome data**	3.1 Were data for this outcome available for all, or nearly all, participants randomized?	Y	All 20 infants analyzed; results for all included (10 per group).
	3.2 If N/PN/NI to 3.1: Is there evidence that result was not biased by missing outcome data?	NA	
	3.3 If N/PN to 3.2: Could missingness in the outcome depend on its true value?	NA	
	3.4 If Y/PY/NI to 3.3: Is it likely that missingness in the outcome depended on its true value?	NA	
	**Risk of bias judgement**	**Low**	No losses to follow-up; complete data.

**Bias in measurement of the outcome**	4.1 Was the method of measuring the outcome inappropriate?	N	Apnea and bradycardia episodes recorded continuously via physiological monitoring.
	4.2 Could measurement or ascertainment of the outcome have differed between intervention groups?	N	Assessments blinded; observer unaware of drug administered.
	4.3 Were outcome assessors aware of the intervention received by study participants?	NI	The report doesn't explicitly state whether outcome assessors were blinded.
	4.4 If Y/PY/NI to 4.3: Could assessment of the outcome have been influenced by knowledge of intervention received?	PN	Primary outcomes are objective (apnea/bradycardia episodes via cardiorespiratory monitoring); assessor influence is unlikely even if aware.
	4.5 If Y/PY/NI to 4.4: Is it likely that assessment of the outcome was influenced by knowledge of intervention received?	NA	
	**Risk of bias judgement**	**Low**	Blinding and objective measurement (monitors).
**Bias in selection of the reported result**	5.1 Were the data that produced this result analysed in accordance with a pre-specified analysis plan that was finalized before unblinded outcome data were available for analysis?	NI	No protocol/SAP available; analysis details (Student t-test) reported but not pre-registered.
	5.2 ... multiple eligible outcome measurements (e.g. scales, definitions, time points) within the outcome domain?	PY	Several outcomes (apnea rate, HR, Na^{+} balance, GI tolerance), but only key results emphasized.
	5.3 ... multiple eligible analyses of the data?		
	**Risk of bias judgement**	**Some concerns**	Lack of pre-specified analysis plan and selective reporting possible.
**Overall bias**	**Risk of bias judgement**	**Some concerns**	Double-blind randomized controlled trial of theophylline vs. caffeine in 20 premature infants with apnea. Allocation likely concealed, groups balanced, and outcomes objectively measured. However, absence of trial registration or analysis plan raises some concerns for selective reporting.

**Unique ID**	6	**Study ID**	Bucher et al (1988)	**Assessor**	

**Ref or Label**	Bucher et al (1988)	**Aim**	assignment to intervention (the 'intention-to-treat' effect)		
**Experimental**	Caffeine citrate (20 mg/kg load at 48 h; 10 mg/kg/day at 72 h & 96 h)	**Comparator**	Placebo (0.9% NaCl)	**Source**	
**Outcome**	Hypoxaemic episodes (≥20% fall in $tcPO_2$ within 20 s), proportion with >6 episodes/12 h (primary); also bradycardia	**Results**	number of hypoxaemic episodes	**Weight**	1

**Domain**	**Signalling question**	**Response**	**Comments**
**Bias arising from the randomization process**	1.1 Was the allocation sequence random?	Y	Phials were numbered in a sequence determined by a table of random numbers; infants received phials in numerical order.
	1.2 Was the allocation sequence concealed until participants were enrolled and assigned to interventions?	Y	Phials prepared in hospital pharmacy; contents not known to investigator nor nursing staff and could not be identified by eye.
	1.3 Did baseline differences between intervention groups suggest a problem with the randomization process?	N	Groups similar at entry for GA, birthweight, sex, delivery mode, Apgar, FiO_2, labs, etc. (Table 1).
	**Risk of bias judgement**	**Low**	Allocation used numbered phials prepared by the hospital pharmacy according to a random-number table; phials were identical in appearance and dispensed in numerical order. Investigators and nursing staff did not know contents, which indicates adequate concealment. Baseline characteristics were well balanced (gestational age, birthweight, sex, delivery mode, Apgar, FiO_2, labs), with no differences suggesting a failed randomization.
**Bias due to deviations from intended interventions**	2.1.Were participants aware of their assigned intervention during the trial?	N	Double-blind; infants cannot be aware; phials identical; staff not aware.
	2.2.Were carers and people delivering the interventions aware of participants' assigned intervention during the trial?	N	Investigators and nursing staff not aware of content; visually indistinguishable phials.
	2.3. If Y/PY/NI to 2.1 or 2.2: Were there deviations from the intended intervention that arose because of the experimental context?	NA	

	2.4 If Y/PY to 2.3: Were these deviations likely to have affected the outcome?	NA	
	2.5. If Y/PY/NI to 2.4: Were these deviations from intended intervention balanced between groups?	NA	
	2.6 Was an appropriate analysis used to estimate the effect of assignment to intervention?	Y	All randomized infants (25 vs 25) reported; “treatment failures” (mechanical ventilation) counted; group comparisons presented—consistent with ITT.
	2.7 If N/PN/NI to 2.6: Was there potential for a substantial impact (on the result) of the failure to analyse participants in the group to which they were randomized?	NA	
	**Risk of bias judgement**	**Low**	Trial reported as double-blind: infants (not aware), carers, and investigators were masked; study medication and placebo were visually indistinguishable. Care pathways and monitoring procedures were the same in both arms, and there is no evidence of protocol-inconsistent co-interventions arising from the trial context. All randomized infants (25 vs 25) appear in the group comparisons, consistent with an appropriate ITT/mITT analysis for this short follow-up window.
**Bias due to missing outcome data**	3.1 Were data for this outcome available for all, or nearly all, participants randomized?	Y	Outcomes reported for all 50 infants (25 per group); no attrition noted across 48–100 h period. (p.2–3, Figs 1–4).
	3.2 If N/PN/NI to 3.1: Is there evidence that result was not biased by missing outcome data?	NA	
	3.3 If N/PN to 3.2: Could missingness in the outcome depend on its true value?	NA	
	3.4 If Y/PY/NI to 3.3: Is it likely that missingness in the outcome depended on its true value?	NA	
	**Risk of bias judgement**	**Low**	Outcome data for the hypoxaemia/bradycardia endpoints are reported for all randomized infants over the predefined 48–100 h window; no attrition or exclusions are described. Given complete (or near-complete) ascertainment in both arms, missingness cannot have depended on true outcome values.

**Bias in measurement of the outcome**	4.1 Was the method of measuring the outcome inappropriate?	N	Continuous $tcPO_2$ and HR monitoring stored digitally; hypoxaemia prespecified (≥20% fall within 20 s); episodes analysed by computer.
	4.2 Could measurement or ascertainment of the outcome have differed between intervention groups?	N	Same monitors/procedures for both arms; central computerized analysis.
	4.3 Were outcome assessors aware of the intervention received by study participants?	N	Episodes validated manually by an examiner blind to the treatment. (p.2, Data collection and evaluation).
	4.4 If Y/PY/NI to 4.3: Could assessment of the outcome have been influenced by knowledge of intervention received?	NA	
	4.5 If Y/PY/NI to 4.4: Is it likely that assessment of the outcome was influenced by knowledge of intervention received?	NA	
	**Risk of bias judgement**	**Low**	Outcomes were measured via continuous transcutaneous PO_2 and heart-rate monitoring with computerized episode detection using predefined thresholds; this method is appropriate and objective. Same devices/procedures were used in both groups, and episodes were manually validated by an examiner blinded to treatment, minimizing differential ascertainment.
**Bias in selection of the reported result**	5.1 Were the data that produced this result analysed in accordance with a pre-specified analysis plan that was finalized before unblinded outcome data were available for analysis?	NI	No protocol/SAP reported; analyses described (χ^2, t-tests), but pre-spec timing/threshold choices not documented.
	5.2 ... multiple eligible outcome measurements (e.g. scales, definitions, time points) within the outcome domain?	PY	Outcome domain includes several 12-h periods and both “proportion with >6 episodes/12 h” and mean frequency; selection emphasis could vary without a protocol. (Results, Figs 1 & 3; 2 & 4).
	5.3 ... multiple eligible analyses of the data?	NI	Only unadjusted χ^2 and t-tests reported; unclear whether alternative analyses were considered or pre-specified.
	**Risk of bias judgement**	**Some concerns**	No pre-registered protocol or SAP is cited, so it’s unclear whether the primary time window/metric (e.g., proportion with >6 episodes per 12 h vs mean frequency, across multiple 12-h blocks) was pre-specified. Because several plausible outcome definitions/time points exist within the same domain and only a subset is emphasized, selective emphasis can’t be excluded even though the

			reported analyses are standard (χ^2/t-tests).
**Overall bias**	**Risk of bias judgement**	**Some concerns**	Domains 1–4 are Low risk (robust randomization/concealment, double-blind conduct, complete data, objective/ blinded outcome assessment). Domain 5 has Some concerns due to lack of a pre-specified analysis plan and potential flexibility in choosing time windows/metrics within the outcome domain.<br><br>Randomization & concealment: Random-number table sequence; pharmacy-prepared, visually identical phials; investigators/nurses unaware. Groups balanced (Table 1).<br><br>Deviations/ITT: Double-blind; identical care; 25 vs 25 analysed with “treatment failures” retained.<br><br>Missing data: None reported for outcome window.<br><br>Measurement: Continuous $tcPO_2$/HR recordings; computer analysis; manual validation by blinded examiner.<br><br>Reporting selection: No protocol/SAP; multiple 12-h windows and two outcome summaries reported (proportion with >6 episodes; mean frequency); choice could be selective.

Unique ID	7	Study ID	Erenberg,et al (2000)	Assessor	
Ref or Label	Erenberg,et al (2000)	Aim	assignment to intervention (the 'intention-to-treat' effect)		
Experimental	Caffeine citrate 10 mg/kg (as base) IV load; 2.5 mg/kg/day (base) IV/PO maintenance (≤10–12 days)	Comparator	Placebo (identical appearance/composition w/o caffeine)	Source	Journal article(s)
Outcome	Apnea burden (≥50% reduction from baseline and elimination of apnea), assessed daily over double-blind phase	Results	68.9% 50% reduction of apnea in caffeine group compared with 43.2% in controls	Weight	1

Domain	Signalling question	Response	Comments
Bias arising from the randomization process	1.1 Was the allocation sequence random?	Y	Computer-generated random numbers in blocks of six.
	1.2 Was the allocation sequence concealed until participants were enrolled and assigned to interventions?	PY	Study drugs prepared centrally; placebo and caffeine solutions were clear and indistinguishable, investigators/nurses not aware of contents; explicit concealment mechanism not fully detailed.
	1.3 Did baseline differences between intervention groups suggest a problem with the randomization process?	N	Table 1 shows no significant differences (GA, weight, baseline apnea count, sex, race).
	**Risk of bias judgement**	**Low**	Randomized by computer-generated blocks; indistinguishable study solutions; no baseline imbalances.
Bias due to deviations from intended interventions	2.1.Were participants aware of their assigned intervention during the trial?	N	Neonates; trial double-blind; indistinguishable study solutions.
	2.2.Were carers and people delivering the interventions aware of participants' assigned intervention during the trial?	N	Investigators and nursing staff not aware of contents; identical appearance.
	2.3. If Y/PY/NI to 2.1 or 2.2: Were there deviations from the intended intervention that arose because of the experimental context?	NA	

	2.4 If Y/PY to 2.3: Were these deviations likely to have affected the outcome?	NA	
	2.5. If Y/PY/NI to 2.4: Were these deviations from intended intervention balanced between groups?	NA	
	2.6 Was an appropriate analysis used to estimate the effect of assignment to intervention?	PN	Not strict ITT: 2 placebo infants randomized never received drug and were excluded from all analyses; 3 more (2 placebo, 1 caffeine) were excluded from efficacy for <6 baseline events discovered post-randomization. Daily outcomes then analyzed on n=82 with last-value-carried-forward after withdrawal/rescue.
	2.7 If N/PN/NI to 2.6: Was there potential for a substantial impact (on the result) of the failure to analyse participants in the group to which they were randomized?	PY	Post-randomization exclusions (including 2 never-treated placebo) could bias treatment effect; magnitude uncertain.
	**Risk of bias judgement**	**Some concerns**	Double-blind with identical care; but efficacy set excluded 5 randomized infants and used LOCF.
**Bias due to missing outcome data**	3.1 Were data for this outcome available for all, or nearly all, participants randomized?	PY	Efficacy analyses included 82/87 randomized (94%); 5 randomized infants excluded from efficacy (two placebo never dosed; three with <6 baseline events). Within the analyzed set, daily status was carried forward, limiting additional missingness.
	3.2 If N/PN/NI to 3.1: Is there evidence that result was not biased by missing outcome data?	NA	
	3.3 If N/PN to 3.2: Could missingness in the outcome depend on its true value?	NA	
	3.4 If Y/PY/NI to 3.3: Is it likely that missingness in the outcome depended on its true value?	NA	
	**Risk of bias judgement**	**Low**	82/87 randomized included; exclusions could relate to outcomes; no sensitivity analysis.
**Bias in measurement of the outcome**	4.1 Was the method of measuring the outcome inappropriate?	N	Cardiorespiratory monitors; apnea defined a priori (>20 s), daily counts standardized.
	4.2 Could measurement or ascertainment of the outcome have differed between intervention groups?	N	Same NICU monitoring procedures and schedules used across arms.

	4.3 Were outcome assessors aware of the intervention received by study participants?	N	Double-blind conduct; solutions indistinguishable; staff not aware of allocation.
	4.4 If Y/PY/NI to 4.3: Could assessment of the outcome have been influenced by knowledge of intervention received?	NA	
	4.5 If Y/PY/NI to 4.4: Is it likely that assessment of the outcome was influenced by knowledge of intervention received?	NA	
	**Risk of bias judgement**	**Low**	Objective monitor-based outcome; same procedures; blinded staff.
**Bias in selection of the reported result**	5.1 Were the data that produced this result analysed in accordance with a pre-specified analysis plan that was finalized before unblinded outcome data were available for analysis?	NI	No protocol/SAP reported; methods list χ^2/ANOVA/t-tests and carry-forward but no pre-registration.
	5.2 ... multiple eligible outcome measurements (e.g. scales, definitions, time points) within the outcome domain?	PY	Multiple daily time points (days 1–10) and two success metrics (≥50% reduction; elimination of apnea); selection/emphasis could vary without prespecification.
	5.3 ... multiple eligible analyses of the data?	NI	Only unadjusted comparisons and last-value-carried-forward described; unclear whether alternatives were considered or prespecified.
	**Risk of bias judgement**	**Some concerns**	No protocol/SAP; multiple timepoints and metrics available.
**Overall bias**	**Risk of bias judgement**	**Some concerns**	Randomization and blinding adequate (Domain 1 & 4 = Low). However, post-randomization exclusions and non-strict ITT (Domain 2) and absence of a pre-specified analysis plan with multiple possible outcome summaries/timepoints (Domain 5) yield Some concerns; Missing data domain also Some concerns given exclusions and no sensitivity checks.

**Unique ID**	8	**Study ID**	Schmidt et al (2006)	**Assessor**	

**Ref or Label**	Schmidt et al (2006)	**Aim**	assignment to intervention (the 'intention-to-treat' effect)		
**Experimental**	Caffeine citrate (20 mg/kg load; 5 mg/kg/day maintenance, up-titrate to 10 mg/kg/day; IV→PO allowed)	**Comparator**	Placebo (normal saline), indistinguishable	**Source**	Journal article(s)
**Outcome**	BPD (need for supplemental O_2 at 36 weeks PMA)	**Results**	36.3% BPD in caffeine group compared with 46.9% in control group	**Weight**	1

**Domain**	**Signalling question**	**Response**	**Comments**
**Bias arising from the randomization process**	1.1 Was the allocation sequence random?	Y	Computer-generated sequence; stratified by center; random blocks of size 2 or 4.
	1.2 Was the allocation sequence concealed until participants were enrolled and assigned to interventions?	Y	Prespecified assignment lists were held only by designated pharmacists; access restricted; investigators/clinical staff masked.
	1.3 Did baseline differences between intervention groups suggest a problem with the randomization process?	N	Maternal/infant characteristics well balanced at baseline (Table 1).
	**Risk of bias judgement**	**Low**	Computer-generated, stratified, blocked randomization; pharmacist-controlled concealment; balanced baseline.
**Bias due to deviations from intended interventions**	2.1.Were participants aware of their assigned intervention during the trial?	N	Double-blind; neonates cannot be aware; study solutions indistinguishable.
	2.2.Were carers and people delivering the interventions aware of participants' assigned intervention during the trial?	N	Only pharmacists and the external safety committee had access; bedside staff masked.
	2.3. If Y/PY/NI to 2.1 or 2.2: Were there deviations from the intended intervention that arose because of the experimental context?	NA	
	2.4 If Y/PY to 2.3: Were these deviations likely to have affected the outcome?	NA	
	2.5. If Y/PY/NI to 2.4: Were these deviations from intended intervention balanced between groups?	NA	
	2.6 Was an appropriate analysis used to estimate the effect of assignment to intervention?	Y	All 2006 randomized infants followed to first discharge; short-term outcomes analyzed with center-adjusted logistic regression (ITT principle).

	2.7 If N/PN/NI to 2.6: Was there potential for a substantial impact (on the result) of the failure to analyse participants in the group to which they were randomized?	NA	
	**Risk of bias judgement**	**Low**	Double-blind; deviations (some open-label methylxanthine) occurred but masking/ITT preserved; center-adjusted models.
**Bias due to missing outcome data**	3.1 Were data for this outcome available for all, or nearly all, participants randomized?	PY	BPD defined among infants alive at 36 weeks PMA (963/1006 caffeine; 954/1000 placebo); deaths (≈5% both arms) are reported separately; follow-up to discharge for all.
	3.2 If N/PN/NI to 3.1: Is there evidence that result was not biased by missing outcome data?	NA	
	3.3 If N/PN to 3.2: Could missingness in the outcome depend on its true value?	NA	
	3.4 If Y/PY/NI to 3.3: Is it likely that missingness in the outcome depended on its true value?	NA	
	**Risk of bias judgement**	**Low**	All infants followed to discharge; BPD assessed among those alive at 36 weeks; mortality similar across arms.
**Bias in measurement of the outcome**	4.1 Was the method of measuring the outcome inappropriate?	N	Standard clinical definition: supplemental oxygen at 36 weeks PMA; widely accepted BPD criterion.
	4.2 Could measurement or ascertainment of the outcome have differed between intervention groups?	N	Same hospital practices within centers; analysis adjusted for center to address practice variability.
	4.3 Were outcome assessors aware of the intervention received by study participants?	N	Trial double-blind; caregivers and outcome assessors masked to allocation.
	4.4 If Y/PY/NI to 4.3: Could assessment of the outcome have been influenced by knowledge of intervention received?	NA	
	4.5 If Y/PY/NI to 4.4: Is it likely that assessment of the outcome was influenced by knowledge of intervention received?	NA	
	**Risk of bias judgement**	**Low**	Standard BPD definition; same procedures; assessors masked; analysis adjusted for center.
**Bias in selection of the reported result**	5.1 Were the data that produced this result analysed in accordance with a pre-specified analysis plan that was finalized before unblinded outcome data were available for analysis?	Y	Trial registered (ClinicalTrials.gov noted in paper) with protocol-specified short-term outcomes.

	5.2 ... multiple eligible outcome measurements (e.g. scales, definitions, time points) within the outcome domain?	N	BPD defined a priori as oxygen at 36 weeks PMA; no alternative BPD definitions/timepoints reported for selection.
	5.3 ... multiple eligible analyses of the data?	PN	Primary analysis adjusted for center (pre-planned); additional adjustment for key covariates shown but consistent; no evidence of selective model choice driving conclusions.
	**Risk of bias judgement**	**Low**	Registered trial with pre-specified short-term outcomes; fixed BPD definition; analyses appropriate and consistent
**Overall bias**	**Risk of bias judgement**	**Low**	Robust randomization/concealment, double-blinding, complete short-term ascertainment, objective outcome, prespecified analyses.

**Unique ID**	9	**Study ID**	Armanian et al (2015)	**Assessor**	
**Ref or Label**	Armanian et al (2015)	**Aim**	assignment to intervention (the 'intention-to-treat' effect)		
**Experimental**	Caffeine 20 mg/kg IV load day 1 + 5 mg/kg IV daily × 10 day	**Comparator**	Placebo (0.9 % saline IV daily × 10 days)	**Source**	Journal article(s)
**Outcome**	Incidence of apnea ≥ 20 s + bradycardia/cyanosis in first 10 days of life	**Results**	15.4% in the caffeine group developed apnea compared with 61.50% in control group	**Weight**	1

**Domain**	**Signalling question**	**Response**	**Comments**
**Bias arising from the randomization process**	1.1 Was the allocation sequence random?	Y	Computer-generated list prepared by independent statistician.
	1.2 Was the allocation sequence concealed until participants were enrolled and assigned to interventions?	Y	Caffeine & placebo drawn into identical numbered syringes by blinded nurse; investigator unaware of numbering.

	1.3 Did baseline differences between intervention groups suggest a problem with the randomization process?	N	Groups balanced for sex, GA, birth weight (Table 1 p. 411).
	**Risk of bias judgement**	**Low**	Random computer sequence; identical numbered syringes; concealed; baseline balanced
**Bias due to deviations from intended interventions**	2.1.Were participants aware of their assigned intervention during the trial?	N	Infants blinded; solutions identical.<br><br>Investigators and nurses blinded to contents.
	2.2.Were carers and people delivering the interventions aware of participants' assigned intervention during the trial?	N	
	2.3. If Y/PY/NI to 2.1 or 2.2: Were there deviations from the intended intervention that arose because of the experimental context?	NA	
	2.4 If Y/PY to 2.3: Were these deviations likely to have affected the outcome?	NA	
	2.5. If Y/PY/NI to 2.4: Were these deviations from intended intervention balanced between groups?	NA	
	2.6 Was an appropriate analysis used to estimate the effect of assignment to intervention?	PY	1 infant (caffeine group) never dosed and 5 (2 + 3) withdrew for sepsis; analysis performed on 26 vs 26 per protocol.
	2.7 If N/PN/NI to 2.6: Was there potential for a substantial impact (on the result) of the failure to analyse participants in the group to which they were randomized?	NA	
	**Risk of bias judgement**	**Some concerns**	Double-blind conduct; protocol consistent across arms; one infant not treated and five withdrew for sepsis.
**Bias due to missing outcome data**	3.1 Were data for this outcome available for all, or nearly all, participants randomized?	Y	52/58 (90 %) completed; all analyzed for primary outcome.
	3.2 If N/PN/NI to 3.1: Is there evidence that result was not biased by missing outcome data?	NA	
	3.3 If N/PN to 3.2: Could missingness in the outcome depend on its true value?	NA	
	3.4 If Y/PY/NI to 3.3: Is it likely that missingness in the outcome depended on its true value?	NA	
	**Risk of bias judgement**	**Low**	52/58 analyzed with complete outcomes.

Bias in measurement of the outcome	4.1 Was the method of measuring the outcome inappropriate?	N	Apnea ≥ 20 s + bradycardia/cyanosis recorded from NICU monitors and daily reports.
	4.2 Could measurement or ascertainment of the outcome have differed between intervention groups?	N	Same monitors and staff for both arms.
	4.3 Were outcome assessors aware of the intervention received by study participants?	N	Certified personnel blinded to randomization recorded outcomes.
	4.4 If Y/PY/NI to 4.3: Could assessment of the outcome have been influenced by knowledge of intervention received?	NA	
	4.5 If Y/PY/NI to 4.4: Is it likely that assessment of the outcome was influenced by knowledge of intervention received?	NA	
	**Risk of bias judgement**	**Low**	Objective monitor-based measurements by blinded personnel.
**Bias in selection of the reported result**	5.1 Were the data that produced this result analysed in accordance with a pre-specified analysis plan that was finalized before unblinded outcome data were available for analysis?	PY	Trial registered (IRCT2013110610026N3); protocol not published but registration before analysis.
	5.2 ... multiple eligible outcome measurements (e.g. scales, definitions, time points) within the outcome domain?	N	Single definition of apnea/bradycardia/cyanosis; no alternate time points.
	5.3 ... multiple eligible analyses of the data?	PN	Basic t-test/logistic regression specified; no evidence of selective modeling.
	**Risk of bias judgement**	**Low**	Registered trial; single predefined outcome; basic statistical plan
**Overall bias**	**Risk of bias judgement**	**Some concerns**	Randomization and blinding robust; complete data and objective measurements. Minor concerns arise from small sample and slight post-randomization losses (ITT not strict).

**Unique ID**	10	**Study ID**	Wei et al (2016)	**Assessor**	

**Ref or Label**	Wei et al (2016)	**Aim**	assignment to intervention (the 'intention-to-treat' effect)		
**Experimental**	Early caffeine: 20 mg/kg IV load at 12–24 h after birth + 8 mg/kg/day IV maintenance	**Comparator**	Late caffeine: same dose 4–6 h before planned extubation	**Source**	
**Outcome**	Need for ventilatory support (PIP, FiO_2, ventilation/NCPAP/oxygen duration) and apnea incidence after extubation	**Results**		**Weight**	1

**Domain**	**Signalling question**	**Response**	**Comments**
**Bias arising from the randomization process**	1.1 Was the allocation sequence random?	Y	Random number table method" used to assign 59 infants (30 vs 29).
	1.2 Was the allocation sequence concealed until participants were enrolled and assigned to interventions?	NI	Randomization described, but no concealment details (e.g., sealed envelopes not stated).
	1.3 Did baseline differences between intervention groups suggest a problem with the randomization process?	N	Table 1 shows no significant differences in GA, weight, sex, RDS stage, infection, antenatal steroids.
	**Risk of bias judgement**	**Some concerns**	Random number table used; concealment not described; baseline balanced
**Bias due to deviations from intended interventions**	2.1.Were participants aware of their assigned intervention during the trial?	N	Neonates cannot be aware.
	2.2.Were carers and people delivering the interventions aware of participants' assigned intervention during the trial?	NI	Open-label described as "prospective controlled," but blinding not mentioned; likely unblinded clinicians.
	2.3. If Y/PY/NI to 2.1 or 2.2: Were there deviations from the intended intervention that arose because of the experimental context?	PY	Care teams may have altered weaning decisions given knowledge of early vs late caffeine.
	2.4 If Y/PY to 2.3: Were these deviations likely to have affected the outcome?	PY	Clinicians were likely unblinded; knowing a baby was in the early-caffeine arm could plausibly influence timing of extubation/weaning and ventilator settings (PIP/FiO_2)—which are the study outcomes. The trial contrasts caffeine at 12–24 h after birth vs only 4–6 h before planned extubation, leaving room for expectation-driven care differences that affect results.

	2.5. If Y/PY/NI to 2.4: Were these deviations from intended intervention balanced between groups?	PN	Any expectation-driven deviations (earlier weaning, extubation decisions) would systematically favor the early-caffeine group given the design; the paper does not document procedures ensuring balance of such deviations across arms.
	2.6 Was an appropriate analysis used to estimate the effect of assignment to intervention?	Y	All 59 infants analyzed by assigned group; no attrition reported (Table 3 includes 59).
	2.7 If N/PN/NI to 2.6: Was there potential for a substantial impact (on the result) of the failure to analyse participants in the group to which they were randomized?	NA	
	**Risk of bias judgement**	**Some concerns**	Likely open-label; early vs late caffeine may influence care decisions
**Bias due to missing outcome data**	3.1 Were data for this outcome available for all, or nearly all, participants randomized?	Y	59 randomized, all included in analysis (Tables 1–3 show 30 + 29 = 59).
	3.2 If N/PN/NI to 3.1: Is there evidence that result was not biased by missing outcome data?	NA	
	3.3 If N/PN to 3.2: Could missingness in the outcome depend on its true value?	NA	
	3.4 If Y/PY/NI to 3.3: Is it likely that missingness in the outcome depended on its true value?	NA	
	**Risk of bias judgement**	**Low**	All 59 infants analyzed; no attrition
**Bias in measurement of the outcome**	4.1 Was the method of measuring the outcome inappropriate?	N	Objective measures: ventilator parameters (PIP, FiO_2), duration (hours/days), monitored apnea counts.
	4.2 Could measurement or ascertainment of the outcome have differed between intervention groups?	PY	Same NICU and protocols, but unblinded care could influence timing of extubation/weaning.
	4.3 Were outcome assessors aware of the intervention received by study participants?	NA	
	4.4 If Y/PY/NI to 4.3: Could assessment of the outcome have been influenced by knowledge of intervention received?	NA	
	4.5 If Y/PY/NI to 4.4: Is it likely that assessment of the outcome was influenced by knowledge of intervention received?	NA	

	**Risk of bias judgement**	**Some concerns**	Objective ventilator data but unblinded staff; extubation/VAP assessments subjective
**Bias in selection of the reported result**	5.1 Were the data that produced this result analysed in accordance with a pre-specified analysis plan that was finalized before unblinded outcome data were available for analysis?	NI	No protocol or registration reported.
	5.2 ... multiple eligible outcome measurements (e.g. scales, definitions, time points) within the outcome domain?	PY	Several endpoints (PIP, FiO_2, intubation time, NCPAP time, VAP, AOP) could be highlighted selectively.
	5.3 ... multiple eligible analyses of the data?	NI	Only simple t/χ^2 tests reported; no mention of adjusted models or hierarchy.
	**Risk of bias judgement**	**Some concerns**	No pre-registration; multiple outcomes/timepoints; simple analyses.
**Overall bias**	**Risk of bias judgement**	**Some concerns**	Random sequence adequate, but concealment and blinding unclear. Outcome measurement and reporting could be affected by open-label design, though missing data minimal and objective metrics used.

**Unique ID**	11	**Study ID**	Dekker et al (2017)	**Assessor**	
**Ref or Label**	Dekker et al (2017)	**Aim**	assignment to intervention (the 'intention-to-treat' effect)		
**Experimental**	Caffeine base 10 mg/kg IV bolus in the delivery room within ~7 min after birth; then 5 mg/kg at 24 h	**Comparator**	Same caffeine regimen started later (median ~48 min) after NICU arrival	**Source**	
**Outcome**	Respiratory effort at 7–9 min after birth (minute volume (MV), tidal volume, respiratory rate, rate-of-rise, % recruitment breaths).	**Results**	MV = 189 ± 74 mL/kg/min vs 162 ± 70 mL/kg/min at 7–9 min (Δ = +27 mL/kg/min favoring early caffeine).	**Weight**	1

Domain	Signalling question	Response	Comments
**Bias arising from the randomization process**	1.1 Was the allocation sequence random?	Y	Computer-generated, stratified by GA; blocks of 4; sequentially numbered sealed envelopes.
	1.2 Was the allocation sequence concealed until participants were enrolled and assigned to interventions?	Y	Sealed, sequentially numbered envelopes; allocation performed per protocol.
	1.3 Did baseline differences between intervention groups suggest a problem with the randomization process?	PY	Significant GA imbalance: caffeine-DR infants were younger GA; authors performed post-hoc GA-adjusted analyses.
	**Risk of bias judgement**	**Some concerns**	Randomized with sealed envelopes/blocks; GA imbalance at baseline
**Bias due to deviations from intended interventions**	2.1.Were participants aware of their assigned intervention during the trial?	N	Neonates cannot be aware..
	2.2.Were carers and people delivering the interventions aware of participants' assigned intervention during the trial?	Y	Open-label timing (caffeine at birth vs later); no blinding of clinicians.
	2.3. If Y/PY/NI to 2.1 or 2.2: Were there deviations from the intended intervention that arose because of the experimental context?	PY	Knowledge of group could influence mask-ventilation assistance and timing/pressure titration during the first minutes.
	2.4 If Y/PY to 2.3: Were these deviations likely to have affected the outcome?	PY	Early clinical support choices (pressures/assistance) could alter MV and tidal volumes measured at 7–9 min—the primary outcomes.
	2.5. If Y/PY/NI to 2.4: Were these deviations from intended intervention balanced between groups?	NI	Paper doesn’t document whether any expectation-driven care deviations were standardized or balanced across arms.
	2.6 Was an appropriate analysis used to estimate the effect of assignment to intervention?	PN	Not strict ITT: 30 randomized → 23 analyzed (exclusions for no recordings, protocol-timing violation, and being apneic at 7–9 min).
	2.7 If N/PN/NI to 2.6: Was there potential for a substantial impact (on the result) of the failure to analyse participants in the group to which they were randomized?	PY	Excluding infants apneic at 7–9 min and those without recordings can bias MV upward; impact plausible given small sample.
	**Risk of bias judgement**	**Some concerns**	Open-label timing; likely care deviations; non-ITT subset analysis; exclusions could affect MV.
**Bias due to missing**	3.1 Were data for this outcome available for all, or nearly all, participants randomized?	N	Only 23/30 infants contributed primary outcome (7 exclusions).

outcome data	3.2 If N/PN/NI to 3.1: Is there evidence that result was not biased by missing outcome data?	N	No sensitivity analyses for exclusions.
	3.3 If N/PN to 3.2: Could missingness in the outcome depend on its true value?	Y	Infants apneic at 7–9 min had no MV value—missingness likely linked to low MV.
	3.4 If Y/PY/NI to 3.3: Is it likely that missingness in the outcome depended on its true value?	PY	Directionally plausible and impactful in a small trial.
	**Risk of bias judgement**	**High**	23/30 analysed; missingness related to apnea/low MV; no sensitivity analyses.
**Bias in measurement of the outcome**	4.1 Was the method of measuring the outcome inappropriate?	N	Standard respiratory function monitor; minute-by-minute MV, Vti, RoR; predefined window (7–9 min).
	4.2 Could measurement or ascertainment of the outcome have differed between intervention groups?	PY	Unblinded care during stabilization could influence support while measuring.
	4.3 Were outcome assessors aware of the intervention received by study participants?	NA	
	4.4 If Y/PY/NI to 4.3: Could assessment of the outcome have been influenced by knowledge of intervention received?	NA	
	4.5 If Y/PY/NI to 4.4: Is it likely that assessment of the outcome was influenced by knowledge of intervention received?	NA	
	**Risk of bias judgement**	**Some concerns**	Objective monitor outputs, but assessors unblinded.
**Bias in selection of the reported result**	5.1 Were the data that produced this result analysed in accordance with a pre-specified analysis plan that was finalized before unblinded outcome data were available for analysis?	NI	No public protocol/registration; post-hoc GA adjustment performed.
	5.2 ... multiple eligible outcome measurements (e.g. scales, definitions, time points) within the outcome domain?	PY	Several respiratory-effort metrics (MV, Vti, RoR, recruitment breaths, RR) and two time windows (pre/post dose within DR group).
	5.3 ... multiple eligible analyses of the data?	NI	Basic group comparisons; exclusions and post-hoc adjustment introduce flexibility.
	**Risk of bias judgement**	**Some concerns**	No preregistered SAP; multiple metrics/time-windows; post-hoc GA adjustment
**Overall bias**	**Risk of bias judgement**	**High**	substantial missing outcome data linked to clinical status (apnea), unblinded care with possible deviations, baseline GA imbalance, and analytic

			departures from strict ITT in a very small sample.

**Unique ID**	12	**Study ID**	Doyle et al (2017)	**Assessor**	
**Ref or Label**	Doyle et al (2017)	**Aim**	assignment to intervention (the 'intention-to-treat' effect)		
**Experimental**	Neonatal caffeine citrate (20 mg/kg load; 5–10 mg/kg/day maintenance, per CAP)	**Comparator**	Neonatal placebo	**Source**	Journal article(s)
**Outcome**	Expiratory flows at 11 years (FEV_1, FVC, FEV_1/FVC, $FEF_{25–75}$%), z-scores (GLI), % predicted; “low” defined as <5th centile	**Results**	FEV_1 = −1.00 ± 1.17 z vs −1.53 ± 1.35 z (Δ = +0.54 z; 95 % CI 0.14–0.94; p = 0.008). FVC Δ = +0.44 z (p = 0.031); $FEF_{25–75}$ Δ = +0.45 z (p = 0.028). Low FVC < 5th centile: 11 % vs 28 %, OR 0.27 (0.10–0.74). Differences attenuated after BPD adjustment, suggesting mediation via improved neonatal respiratory outcomes.	**Weight**	1

**Domain**	**Signalling question**	**Response**	**Comments**
**Bias arising from the randomization process**	1.1 Was the allocation sequence random?	Y	Computer-generated CAP randomization; 2,006 infants overall; this is the Melbourne subset.
	1.2 Was the allocation sequence concealed until participants were enrolled and assigned to interventions?	Y	CAP was double-blind with concealed allocation; identical caffeine/placebo solutions.

	1.3 Did baseline differences between intervention groups suggest a problem with the randomization process?	PN	At this center, some prerandomization differences (antenatal steroids higher; male proportion trend) but no indication the sequence wasn't random; main neonatal outcomes favored caffeine as in CAP.
	**Risk of bias judgement**	**Low**	CAP neonatal randomization and concealment; minor prerandomization imbalances at this site; no sign of compromised sequence.
**Bias due to deviations from intended interventions**	2.1.Were participants aware of their assigned intervention during the trial?	N	Neonatal phase double-blind; 11-year children not informed.<br><br>Neonatal clinicians were blinded in CAP; 11-year assessors blinded to group.
	2.2.Were carers and people delivering the interventions aware of participants' assigned intervention during the trial?	N	
	2.3. If Y/PY/NI to 2.1 or 2.2: Were there deviations from the intended intervention that arose because of the experimental context?	NA	
	2.4 If Y/PY to 2.3: Were these deviations likely to have affected the outcome?	NA	
	2.5. If Y/PY/NI to 2.4: Were these deviations from intended intervention balanced between groups?	NA	
	2.6 Was an appropriate analysis used to estimate the effect of assignment to intervention?	Y	Group comparisons by original assignment; GEE with robust SEs; adjusted for baseline perinatal covariates; ITT principle for randomized groups.
	2.7 If N/PN/NI to 2.6: Was there potential for a substantial impact (on the result) of the failure to analyse participants in the group to which they were randomized?	NA	
	**Risk of bias judgement**	**Low**	Double-blind neonatal phase; 11-year assessors blinded; appropriate group-wise analysis.
**Bias due to missing outcome data**	3.1 Were data for this outcome available for all, or nearly all, participants randomized?	PN	At this center, 182 survivors; 137 (75%) had usable spirometry; plus 5 from other centers → 142 total with data. Follow-up <100%.
	3.2 If N/PN/NI to 3.1: Is there evidence that result was not biased by missing outcome data?	PY	Authors state perinatal characteristics were similar in those with vs without lung data (Table E1), mitigating risk
	3.3 If N/PN to 3.2: Could missingness in the outcome depend on its true value?	NA	
	3.4 If Y/PY/NI to 3.3: Is it likely that missingness in the outcome depended on its true value?	NA	

	**Risk of bias judgement**	**Some concerns**	142 with spirometry (≈75% of survivors); baseline similarities in those with vs without data reduce bias but don't eliminate it.
**Bias in measurement of the outcome**	4.1 Was the method of measuring the outcome inappropriate?	N	Spirometry followed ATS/ERS standards; GLI reference used.
	4.2 Could measurement or ascertainment of the outcome have differed between intervention groups?	N	Same protocols/equipment; single center testing.
	4.3 Were outcome assessors aware of the intervention received by study participants?	N	Children "assessed blinded to knowledge of treatment."
	4.4 If Y/PY/NI to 4.3: Could assessment of the outcome have been influenced by knowledge of intervention received?	NA	
	4.5 If Y/PY/NI to 4.4: Is it likely that assessment of the outcome was influenced by knowledge of intervention received?	NA	
	**Risk of bias judgement**	**Low**	ATS/ERS-standard spirometry; GLI z-scores; blinded assessment.
**Bias in selection of the reported result**	5.1 Were the data that produced this result analysed in accordance with a pre-specified analysis plan that was finalized before unblinded outcome data were available for analysis?	NI	CAP was registered; this lung-function substudy at one site wasn't pre-specified in the main trial publications; SAP not provided.
	5.2 ... multiple eligible outcome measurements (e.g. scales, definitions, time points) within the outcome domain?	PY	Several spirometric indices (FEV_1, FVC, FEV_1/FVC, FEF_{25-75}%) and "low" vs continuous metrics allow analytic flexibility.
	5.3 ... multiple eligible analyses of the data?	PY	Unadjusted vs adjusted models; analyses with/without BPD covariate; multiple ways to summarize.
	**Risk of bias judgement**	**Some concerns**	Registered parent trial but single-centre lung-function substudy without published SAP; multiple indices/analyses.
**Overall bias**	**Risk of bias judgement**	**Some concerns**	Randomization/concealment and outcome measurement are strong and blinded; analysis compares randomized groups appropriately. Primary limitation is incomplete follow-up (≈75% of survivors with spirometry) and analytic flexibility in a single-site substudy.

**Unique ID**	13	**Study ID**	Habibi et al (2019)	**Assessor**	
**Ref or Label**	Habibi et al (2019)	**Aim**	assignment to intervention (the 'intention-to-treat' effect)		
**Experimental**	Caffeine IV 20 mg/kg load, then 5 mg/kg/day in 2 doses for 5–7 days	**Comparator**	Aminophylline IV 5–7 mg/kg load, then 1–2 mg/kg q6–12h for 5–7 days	**Source**	Journal article(s)
**Outcome**	Recurrence of apnea during treatment (5–7 days); GI adverse effects	**Results**	Apnea recurrence: 3.2% (1/31) aminophylline vs 0% (0/36) caffeine; GI adverse effects 22.6% vs 13.9% (p=0.3). No significant difference between groups.	**Weight**	1

Domain	Signalling question	Response	Comments
**Bias arising from the randomization process**	1.1 Was the allocation sequence random?	PY	Cards labeled for treatment were prepared by a person not involved; parents drew a card from identical sealed envelopes. "Randomly divided" is stated, but no explicit random sequence (e.g., random number list) is described.<br><br>Identical envelopes; treatment identity masked from parents and investigators at assignment.
	1.2 Was the allocation sequence concealed until participants were enrolled and assigned to interventions?	Y	
	1.3 Did baseline differences between intervention groups suggest a problem with the randomization process?	N	No significant differences for sex, GA, birthweight categories, apnea onset/duration, or associated symptoms (Table 1).
	**Risk of bias judgement**	**Low**	Randomly divided with identical sealed envelopes; baseline balanced; sequence method not fully described.
**Bias due to deviations**	2.1.Were participants aware of their assigned intervention during the trial?	N	Neonates cannot be aware; trial reported double-blind.

**from intended interventions**	2.2.Were carers and people delivering the interventions aware of participants' assigned intervention during the trial?	NI	Trial called "double-blind" and project staff/parents were not informed, but bedside blinding is not explicitly detailed; dosing schedules differed (q6–12h vs BID) which could unblind staff.
	2.3. If Y/PY/NI to 2.1 or 2.2: Were there deviations from the intended intervention that arose because of the experimental context?	NI	No deviations reported; same escalation plan (CPAP/intubation) for non-responders.
	2.4 If Y/PY to 2.3: Were these deviations likely to have affected the outcome?	NA	
	2.5. If Y/PY/NI to 2.4: Were these deviations from intended intervention balanced between groups?	NA	
	2.6 Was an appropriate analysis used to estimate the effect of assignment to intervention?	Y	All 67 randomized infants were analyzed by assigned group with χ^2 tests; no post-randomization exclusions reported.
	2.7 If N/PN/NI to 2.6: Was there potential for a substantial impact (on the result) of the failure to analyse participants in the group to which they were randomized?	NA	
	**Risk of bias judgement**	**Some concerns**	Claimed double-blind; assessor blinded; bedside blinding unclear given dosing frequency differences; ITT used.
**Bias due to missing outcome data**	3.1 Were data for this outcome available for all, or nearly all, participants randomized?	Y	Results reported for 31 aminophylline and 36 caffeine (total = 67).
	3.2 If N/PN/NI to 3.1: Is there evidence that result was not biased by missing outcome data?	NA	
	3.3 If N/PN to 3.2: Could missingness in the outcome depend on its true value?	NA	
	3.4 If Y/PY/NI to 3.3: Is it likely that missingness in the outcome depended on its true value?	NA	
	**Risk of bias judgement**	**Low**	67/67 analysed; no attrition.
**Bias in measurement of the outcome**	4.1 Was the method of measuring the outcome inappropriate?	N	Apnea defined clinically (≥15–20 s or shorter with bradycardia/cyanosis) using neonatologist exam, monitors, and pulse oximetry.
	4.2 Could measurement or ascertainment of the outcome have differed between intervention groups?	N	Same NICU, same monitoring/criteria for both arms.

	4.3 Were outcome assessors aware of the intervention received by study participants?	N	Data recorded on a checklist by someone unaware of treatment.
	4.4 If Y/PY/NI to 4.3: Could assessment of the outcome have been influenced by knowledge of intervention received?	NA	
	4.5 If Y/PY/NI to 4.4: Is it likely that assessment of the outcome was influenced by knowledge of intervention received?	NA	
	**Risk of bias judgement**	**Low**	Objective clinical/monitor outcomes; blinded recorder.
**Bias in selection of the reported result**	5.1 Were the data that produced this result analysed in accordance with a pre-specified analysis plan that was finalized before unblinded outcome data were available for analysis?	NI	No registration or public SAP reported
	5.2 ... multiple eligible outcome measurements (e.g. scales, definitions, time points) within the outcome domain?	N	Primary domain was apnea recurrence (yes/no) during treatment; no alternative scales/time-windows reported.
	5.3 ... multiple eligible analyses of the data?	NI	Only basic χ^2 comparisons shown; analytic flexibility not described.
	**Risk of bias judgement**	**Some concerns**	No registration/SAP; simple analyses; single primary measure.
**Overall bias**	**Risk of bias judgement**	**Some concerns**	Good blinding of assessors and complete data; however, sequence generation not fully detailed and bedside blinding could have been compromised by differing dosing schedules; no published protocol/SAP.

**Unique ID**	14	**Study ID**	Lookza et al (2019)	**Assessor**	
**Ref or Label**	Lookza et al (2019)	**Aim**	assignment to intervention (the 'intention-to-treat' effect)		

**Experimental**	Caffeine 30 mg/kg IV load; 10 mg/kg IV q24h maintenance (72 h observation)	**Comparator**	Aminophylline 5 mg/kg IV load; 2 mg/kg IV q8h maintenance (72 h)	**Source**	Journal article(s)
**Outcome**	Apnea within 72 h (yes/no); respiratory support mode (CPAP vs oxyhood); adverse effects	**Results**	Apnea ≤72 h: 17.5% (caffeine) vs 32.5% (aminophylline), p=0.121. CPAP use higher with caffeine (95% vs 75%, p=0.012); oxyhood use lower (5% vs 25%, p=0.012). Adverse effects similar.	**Weight**	1

Domain	Signalling question	Response	Comments
**Bias arising from the randomization process**	1.1 Was the allocation sequence random?	Y	Randomization was done using random numbers by a researcher not involved in care.
	1.2 Was the allocation sequence concealed until participants were enrolled and assigned to interventions?	NI	No explicit concealment method (e.g., sealed opaque envelopes) described.
	1.3 Did baseline differences between intervention groups suggest a problem with the randomization process?	N	Groups similar for GA, birth weight, age, Apgar, sex, glucose (Table 1).
	**Risk of bias judgement**	**Some concerns**	Random numbers used; concealment not described; baseline balanced.
**Bias due to deviations from intended interventions**	2.1.Were participants aware of their assigned intervention during the trial?	N	Neonates cannot be aware.
	2.2.Were carers and people delivering the interventions aware of participants' assigned intervention during the trial?	NI	Trial not described as blinded; dosing schedules differ (q8h vs q24h), which could reveal assignment.
	2.3. If Y/PY/NI to 2.1 or 2.2: Were there deviations from the intended intervention that arose because of the experimental context?	PY	Knowledge/suspicions of group could influence bedside decisions about respiratory support (CPAP vs oxyhood), which are reported outcomes.
	2.4 If Y/PY to 2.3: Were these deviations likely to have affected the outcome?	PY	Clinician-driven support choices (e.g., CPAP vs oxyhood) could plausibly be influenced by perceived assignment and thus affect reported outcomes.
	2.5. If Y/PY/NI to 2.4: Were these deviations from intended intervention balanced between groups?	NI	No process described to ensure any expectation-driven deviations were balanced across arms.

	2.6 Was an appropriate analysis used to estimate the effect of assignment to intervention?	Y	All 80 randomized infants (40/40) appear in the analyses with χ^2 tests; no post-randomization exclusions stated.
	2.7 If N/PN/NI to 2.6: Was there potential for a substantial impact (on the result) of the failure to analyse participants in the group to which they were randomized?	NA	
	**Risk of bias judgement**	**Some concerns**	Likely open-label at bedside; near-ITT analysis; support decisions could be expectation-sensitive.
**Bias due to missing outcome data**	3.1 Were data for this outcome available for all, or nearly all, participants randomized?	Y	Apnea within 72 h and respiratory support reported for all 40 vs 40 (Tables 2–3).
	3.2 If N/PN/NI to 3.1: Is there evidence that result was not biased by missing outcome data?	NA	
	3.3 If N/PN to 3.2: Could missingness in the outcome depend on its true value?	NA	
	3.4 If Y/PY/NI to 3.3: Is it likely that missingness in the outcome depended on its true value?	NA	
	**Risk of bias judgement**	**Low**	40/40 per arm analyzed for primary outcomes.
**Bias in measurement of the outcome**	4.1 Was the method of measuring the outcome inappropriate?	N	Apnea episodes recorded over 72 h with continuous monitoring/clinical checks; respiratory support mode recorded routinely.
	4.2 Could measurement or ascertainment of the outcome have differed between intervention groups?	PY	Open-label conduct likely; bedside documentation and timing of support may vary by expectations.
	4.3 Were outcome assessors aware of the intervention received by study participants?	NA	
	4.4 If Y/PY/NI to 4.3: Could assessment of the outcome have been influenced by knowledge of intervention received?	NA	
	4.5 If Y/PY/NI to 4.4: Is it likely that assessment of the outcome was influenced by knowledge of intervention received?	NA	
	**Risk of bias judgement**	**Some concerns**	Objective monitoring, but assessor blinding not stated; support classification clinician-dependent.
**Bias in selection of the reported result**	5.1 Were the data that produced this result analysed in accordance with a pre-specified analysis plan that was finalized before unblinded outcome data were available for analysis?	NI	No trial registration or SAP reported.

	5.2 ... multiple eligible outcome measurements (e.g. scales, definitions, time points) within the outcome domain?	PY	Multiple outcomes in the same domain: apnea within 72 h, CPAP/oxyhood use, several adverse events.
	5.3 ... multiple eligible analyses of the data?	N	Only basic χ² tests described; flexibility cannot be ruled out.
	**Risk of bias judgement**	**Some concerns**	No registration/SAP; several outcomes reported.
**Overall bias**	**Risk of bias judgement**	**Some concerns**	Randomization reported but concealment and blinding unclear; outcomes partly clinician-driven (support mode) with possible expectation effects; no pre-specified analysis plan. Missing data minimal.

**Unique ID**	15	**Study ID**	Zulqarnain et al (2019)	**Assessor**	
**Ref or Label**	Zulqarnain et al (2019)	**Aim**	assignment to intervention (the 'intention-to-treat' effect)		
**Experimental**	Caffeine citrate 20 mg/kg IV load; 5 mg/kg IV q24 h maintenance; serum levels sampled (day 3, 7, 14, 21)	**Comparator**	Theophylline ≈6 mg/kg load IV; 2 mg/kg q12 h maintenance; TDM targeted 5–12 mg/L	**Source**	Journal article(s)
**Outcome**	Mean apnea events/day over time windows (0; 1–3; 4–7; 8–14; 15–21 days); supportive care and serum levels reported	**Results**	Apnea events/day: at 1–3 days, caffeine 1 ± 0.22 vs theophylline 2 ± 0.12 ($p<0.05$); at 4–7 days, reported significant difference ($p<0.05$). Other windows similar. GA at baseline lower in theophylline arm (30.44 w vs 32.02 w).	**Weight**	1

Domain	Signalling question	Response	Comments
**Bias arising from the randomization process**	1.1 Was the allocation sequence random?	PY	“Randomized control study”; “modification design… computer propagated” is mentioned, but no explicit sequence generation details.<br><br>Group assignment by “lottery method”; concealment method (e.g., sealed opaque envelopes) not described.
	1.2 Was the allocation sequence concealed until participants were enrolled and assigned to interventions?	NI	
	1.3 Did baseline differences between intervention groups suggest a problem with the randomization process?	Y	Substantial GA imbalance between arms (30.44 vs 32.02 weeks, p<0.001); other baseline variables similar.
	**Risk of bias judgement**	**Some concerns**	Randomized trial but concealment not detailed; GA imbalance at baseline
**Bias due to deviations from intended interventions**	2.1.Were participants aware of their assigned intervention during the trial?	N	Neonates cannot be aware.<br><br>Clinicians were not blinded because drugs had different dosing and blood-sampling schedules.
	2.2.Were carers and people delivering the interventions aware of participants' assigned intervention during the trial?	Y	
	2.3. If Y/PY/NI to 2.1 or 2.2: Were there deviations from the intended intervention that arose because of the experimental context?	PY	Open-label context could influence supportive care decisions and interpretation/response to alarms.
	2.4 If Y/PY to 2.3: Were these deviations likely to have affected the outcome?	PY	Caregiver knowledge of drug (and different monitoring schedules) could plausibly affect bedside management and apnea documentation.
	2.5. If Y/PY/NI to 2.4: Were these deviations from intended intervention balanced between groups?	NI	No procedures described to ensure any expectation-driven deviations were balanced.
	2.6 Was an appropriate analysis used to estimate the effect of assignment to intervention?	Y	All 100 randomized appear included (50/50); outcomes compared between randomized groups.
	2.7 If N/PN/NI to 2.6: Was there potential for a substantial impact (on the result) of the failure to analyse participants in the group to which they were randomized?	NA	
	**Risk of bias judgement**	**Some concerns**	Clinicians unblinded; analysis includes all randomized; deviations could affect bedside care; balance unknown
**Bias due to missing**	3.1 Were data for this outcome available for all, or nearly all, participants randomized?	Y	Results presented for all 50 vs 50 across windows; no attrition reported.

outcome data	3.2 If N/PN/NI to 3.1: Is there evidence that result was not biased by missing outcome data?	NA	
	3.3 If N/PN to 3.2: Could missingness in the outcome depend on its true value?	NA	
	3.4 If Y/PY/NI to 3.3: Is it likely that missingness in the outcome depended on its true value?	NA	
	**Risk of bias judgement**	**Low**	Complete outcome ascertainment over windows.
**Bias in measurement of the outcome**	4.1 Was the method of measuring the outcome inappropriate?	N	Continuous cardiopulmonary monitoring and pulse oximetry; predefined apnea definition (>20 s or shorter with hypoxemia/bradycardia).
	4.2 Could measurement or ascertainment of the outcome have differed between intervention groups?	PY	Open-label care and different blood-sampling schedules could influence observation/recording.
	4.3 Were outcome assessors aware of the intervention received by study participants?	NA	
	4.4 If Y/PY/NI to 4.3: Could assessment of the outcome have been influenced by knowledge of intervention received?	NA	
	4.5 If Y/PY/NI to 4.4: Is it likely that assessment of the outcome was influenced by knowledge of intervention received?	NA	
	**Risk of bias judgement**	**Some concerns**	Objective monitoring, but bedside assessors not blinded.
**Bias in selection of the reported result**	5.1 Were the data that produced this result analysed in accordance with a pre-specified analysis plan that was finalized before unblinded outcome data were available for analysis?	NI	No registration/SAP reported.
	5.2 ... multiple eligible outcome measurements (e.g. scales, definitions, time points) within the outcome domain?	PY	Multiple windows (0; 1–3; 4–7; 8–14; 15–21 days) and several supportive indices allow selective emphasis.
	5.3 ... multiple eligible analyses of the data?	NI	Only basic t-tests/χ² reported; analytic flexibility cannot be excluded.
	**Risk of bias judgement**	**Some concerns**	No SAP/registration; multiple time windows.
**Overall bias**	**Risk of bias judgement**	**Some concerns**	Unclear sequence concealment, open-label care with potential deviations, baseline GA imbalance,

			and flexible reporting across several time windows; outcome data otherwise complete with objective monitoring.

**Unique ID**	16	**Study ID**	Liu et al (2020)	**Assessor**	
**Ref or Label**	Liu et al (2020)	**Aim**	assignment to intervention (the 'intention-to-treat' effect)		
**Experimental**	Caffeine citrate 20 mg/kg load, then 5–10 mg/kg once daily; first dose within 72 h after birth until weaning criteria (per NICU protocol).	**Comparator**	Equal-volume saline placebo; usual AOP rescue (aminophylline or naloxone) allowed per clinician discretion.	**Source**	Journal article(s)
**Outcome**	Primary—White matter microstructure at ~34–36 weeks PMA by DTI: fractional anisotropy (FA) and apparent diffusion coefficient (ADC) in 10 predefined white-matter ROIs; Secondary—AOP frequency, ventilation duration, short-term complications.	**Results**	DTI at ~35 w PMA—mean FA↑ and ADC↓ with caffeine: group effect FA $+5.55\times10^{-3}$, ADC -54.53×10^{-6} mm^2/s (both $p<0.001$); e.g., PLIC FA 0.579 vs 0.541; ADC 1307 vs 1394 ($\times10^{-6}$ mm^2/s). AOP rate/24 h 1.24 vs 1.83 ($p = 0.01$); assisted-ventilation duration lower ($p = 0.034$).	**Weight**	1

**Domain**	**Signalling question**	**Response**	**Comments**
**Bias arising from the**	1.1 Was the allocation sequence random?	Y	Computer-based random number generator; 1:1 to caffeine or placebo.

**randomization process**	1.2 Was the allocation sequence concealed until participants were enrolled and assigned to interventions?	NI	Placebo was equal-volume saline, but explicit concealment method (e.g., sealed opaque envelopes/pharmacy control) not reported.
	1.3 Did baseline differences between intervention groups suggest a problem with the randomization process?	N	Maternal/infant baseline characteristics similar between groups (Table 1).
	**Risk of bias judgement**	**Some concerns**	Computer-generated sequence; concealment not detailed; baseline balanced
**Bias due to deviations from intended interventions**	2.1.Were participants aware of their assigned intervention during the trial?	N	Neonates cannot be aware.<br><br>Identical-volume saline used; clinician blinding not explicitly stated; rescue methylxanthines permitted per discretion.
	2.2.Were carers and people delivering the interventions aware of participants' assigned intervention during the trial?	NI	
	2.3. If Y/PY/NI to 2.1 or 2.2: Were there deviations from the intended intervention that arose because of the experimental context?	NI	No protocol-deviation patterns reported; supportive care stated to be identical between groups.
	2.4 If Y/PY to 2.3: Were these deviations likely to have affected the outcome?	NA	
	2.5. If Y/PY/NI to 2.4: Were these deviations from intended intervention balanced between groups?	NA	
	2.6 Was an appropriate analysis used to estimate the effect of assignment to intervention?	PN	Primary DTI outcomes analysed in a per-protocol subset (160/189 with completed MRI+DTI: 80 vs 80); post-randomization exclusions include deaths, NEC surgery, inability to stop oxygen, early discharge/refusal (flowchart, Fig. 2).
	2.7 If N/PN/NI to 2.6: Was there potential for a substantial impact (on the result) of the failure to analyse participants in the group to which they were randomized?	PY	Exclusions (e.g., severe BPD/NRDS deaths, NEC surgery, inability to stop oxygen) are related to illness severity that may correlate with brain microstructure, risking bias in the DTI outcome.
	**Risk of bias judgement**	**Some concerns**	Caregiver blinding not explicit; primary analysis excludes non-DTI completers.
**Bias due to missing outcome data**	3.1 Were data for this outcome available for all, or nearly all, participants randomized?	N	189 randomized (96 caffeine; 93 placebo) → 160 analysed for MRI+DTI (80/80); 29 (15%) had no primary outcome (reasons in Fig. 2).
	3.2 If N/PN/NI to 3.1: Is there evidence that result was not biased by missing outcome data?	N	No sensitivity analyses for missing DTI data reported.

	3.3 If N/PN to 3.2: Could missingness in the outcome depend on its true value?	Y	Exclusions due to illness/death or inability to stop oxygen likely relate to brain microstructure..
	3.4 If Y/PY/NI to 3.3: Is it likely that missingness in the outcome depended on its true value?	PY	Plausible in this context; although counts excluded were similar between arms.
	**Risk of bias judgement**	**Some concerns**	160/189 with DTI (≈85%); exclusions tied to illness.
**Bias in measurement of the outcome**	4.1 Was the method of measuring the outcome inappropriate?	N	DTI-derived FA/ADC in prespecified white-matter ROIs; standard acquisition/analysis.
	4.2 Could measurement or ascertainment of the outcome have differed between intervention groups?	N	Same scanners/protocols; identical ROI set.
	4.3 Were outcome assessors aware of the intervention received by study participants?	NI	Two operators extracted ROI values and cross-checked; paper does not explicitly state masking to group.
	4.4 If Y/PY/NI to 4.3: Could assessment of the outcome have been influenced by knowledge of intervention received?	PN	Quantitative DTI metrics reduce subjective influence.
	4.5 If Y/PY/NI to 4.4: Is it likely that assessment of the outcome was influenced by knowledge of intervention received?	NA	
	**Risk of bias judgement**	**Low**	Quantitative DTI (FA/ADC) with identical protocols; assessor masking not stated but influence limited.
**Bias in selection of the reported result**	5.1 Were the data that produced this result analysed in accordance with a pre-specified analysis plan that was finalized before unblinded outcome data were available for analysis?	NI	No registration/SAP reported.
	5.2 ... multiple eligible outcome measurements (e.g. scales, definitions, time points) within the outcome domain?	PY	Ten WM ROIs for FA and ADC → multiple choices for emphasis.
	5.3 ... multiple eligible analyses of the data?	PY	Univariate t-tests per ROI + regression models; several analytic choices available.
	**Risk of bias judgement**	**Some concerns**	No preregistration; many ROIs/metrics and models.
**Overall bias**	**Risk of bias judgement**	**Some concerns**	Strong randomization; objective DTI measurements. However, per-protocol analysis with post-randomization exclusions and no registered SAP introduce selection and reporting concerns; missing primary outcomes may relate to

			true brain status.

**Unique ID**	17	**Study ID**	Iranpour et al (2020)	**Assessor**	
**Ref or Label**	Iranpour et al (2020)	**Aim**	assignment to intervention (the 'intention-to-treat' effect)		
**Experimental**	Caffeine citrate 20 mg/kg load, then 10 mg/kg daily maintenance (IV → PO when feeding)	**Comparator**	No placebo / usual care (control did not receive similar drug)	**Source**	Journal article(s)
**Outcome**	Duration of NCPAP (hours)	**Results**	NCPAP duration (hours): Caffeine 41.53 ± 43.25 vs Control 78.48 ± 114.25; mean difference −36.95 h (95% CI −73.14 to −0.76), p = 0.04	**Weight**	1

**Domain**	**Signalling question**	**Response**	**Comments**
**Bias arising from the randomization process**	1.1 Was the allocation sequence random?	Y	Random numbers generated by a computer.<br><br>They state allocation not revealed until the study began, but no specific concealment method (e.g., pharmacy control / sealed opaque envelopes).
	1.2 Was the allocation sequence concealed until participants were enrolled and assigned to interventions?	NI	
	1.3 Did baseline differences between intervention groups suggest a problem with the randomization process?	N	No imbalances reported as problematic in text; identical ventilation protocol used across arms. (No explicit baseline table is presented as imbalanced.)
	**Risk of bias judgement**	**Some concerns**	Computer-generated randomization; concealment not detailed; no problematic baseline differences reported.

Bias due to deviations from intended interventions	2.1.Were participants aware of their assigned intervention during the trial?	N	Neonates cannot be aware.
	2.2.Were carers and people delivering the interventions aware of participants' assigned intervention during the trial?	Y	Control received no placebo; authors state the study was not blinded.
	2.3. If Y/PY/NI to 2.1 or 2.2: Were there deviations from the intended intervention that arose because of the experimental context?	PY	Open-label care could influence bedside weaning/pressure titration decisions during NCPAP (identical written protocol, but clinicians knew assignment).
	2.4 If Y/PY to 2.3: Were these deviations likely to have affected the outcome?	PY	Knowledge of receiving caffeine could plausibly hasten NCPAP weaning, directly shortening the duration outcome.
	2.5. If Y/PY/NI to 2.4: Were these deviations from intended intervention balanced between groups?	NI	Paper does not document monitoring to ensure any expectation-driven deviations were balanced.
	2.6 Was an appropriate analysis used to estimate the effect of assignment to intervention?	Y	All 45 vs 45 randomized infants were analyzed by assigned group for the primary outcome.
	2.7 If N/PN/NI to 2.6: Was there potential for a substantial impact (on the result) of the failure to analyse participants in the group to which they were randomized?	NA	
	**Risk of bias judgement**	**High**	Open-label (no placebo); ITT used; clinician knowledge could hasten weaning; balance of such deviations unreported.
Bias due to missing outcome data	3.1 Were data for this outcome available for all, or nearly all, participants randomized?	Y	Primary outcome reported for all 90 randomized (45/45).
	3.2 If N/PN/NI to 3.1: Is there evidence that result was not biased by missing outcome data?	NA	
	3.3 If N/PN to 3.2: Could missingness in the outcome depend on its true value?	NA	
	3.4 If Y/PY/NI to 3.3: Is it likely that missingness in the outcome depended on its true value?	NA	
	**Risk of bias judgement**	**Low**	Primary outcome available for all 90 infants.
Bias in measurement of the outcome	4.1 Was the method of measuring the outcome inappropriate?	N	NCPAP hours recorded under a prespecified weaning protocol (FiO_2 targets; INSURE; stop criteria).

	4.2 Could measurement or ascertainment of the outcome have differed between intervention groups?	PY	Same protocol, but open-label clinicians could apply weaning decisions differently despite identical guidance.
	4.3 Were outcome assessors aware of the intervention received by study participants?	NA	
	4.4 If Y/PY/NI to 4.3: Could assessment of the outcome have been influenced by knowledge of intervention received?	NA	
	4.5 If Y/PY/NI to 4.4: Is it likely that assessment of the outcome was influenced by knowledge of intervention received?	NA	
	**Risk of bias judgement**	**Some concerns**	Standardized NCPAP protocol, but unblinded assessors and clinician-managed outcome
**Bias in selection of the reported result**	5.1 Were the data that produced this result analysed in accordance with a pre-specified analysis plan that was finalized before unblinded outcome data were available for analysis?	PY	Trial registered in IRCT (IRCT20170627034782N2; 2019-12-12); SAP not in article.
	5.2 ... multiple eligible outcome measurements (e.g. scales, definitions, time points) within the outcome domain?	N	Primary outcome was one metric (NCPAP duration).
	5.3 ... multiple eligible analyses of the data?	NI	Basic between-group comparisons; no model selection catalogue provided.
	**Risk of bias judgement**	**Some concerns**	Trial registered (IRCT…N2); single primary metric; limited SAP detail.
**Overall bias**	**Risk of bias judgement**	**Some concerns**	Open-label design with outcome sensitive to clinician decision-making (Domains 2 & 4), and unclear concealment; complete data and registered protocol support confidence otherwise.

**Unique ID**	18	**Study ID**	Oliphant et al (2023)	**Assessor**	
**Ref or Label**	Oliphant et al (2023)	**Aim**	assignment to intervention (the 'intention-to-treat' effect)		

**Experimental**	Enteral caffeine citrate loading 10/20/30/40 mg/kg then 5/10/15/20 mg/kg/day to term-equivalent age.	**Comparator**	Placebo water daily. Same volume for all.	**Source**	Journal article(s)
**Outcome**	Rate of intermittent hypoxaemia (IH) on overnight oximetry 2 weeks post-randomisation (events/h, SpO_2 drop ≥10% below baseline for <2 min). (Methods p.2–3; Table 2 p.4.)	**Results**	Mean SpO_2 higher and % time SpO_2<90% lower with 10 and 20 mg/kg/day; more tachycardia time with caffeine (dose-related). (Table 2 p.4; Results p.6.)	**Weight**	1

Domain	Signalling question	Response	Comments
**Bias arising from the randomization process**	1.1 Was the allocation sequence random?	Y	Internet randomisation service; varying block sizes; stratified by site and GA.
	1.2 Was the allocation sequence concealed until participants were enrolled and assigned to interventions?	Y	Identical-appearing, equal-volume trial meds; parents/clinicians/assessors blinded.
	1.3 Did baseline differences between intervention groups suggest a problem with the randomization process?	N	aseline characteristics similar across groups (Table 1).
	**Risk of bias judgement**	**Low**	Internet randomisation; concealed, double-blind; balanced baseline.
**Bias due to deviations from intended interventions**	2.1.Were participants aware of their assigned intervention during the trial?	N	Neonates cannot be aware. All investigators were blinded.
	2.2.Were carers and people delivering the interventions aware of participants' assigned intervention during the trial?	N	
	2.3. If Y/PY/NI to 2.1 or 2.2: Were there deviations from the intended intervention that arose because of the experimental context?	NA	
	2.4 If Y/PY to 2.3: Were these deviations likely to have affected the outcome?	NA	
	2.5. If Y/PY/NI to 2.4: Were these deviations from intended intervention balanced between groups?	NA	
	2.6 Was an appropriate analysis used to estimate the effect of assignment to intervention?	Y	Prespecified ITT using GLMMs; adjusted for site/GA and clustering of multiples.

	2.7 If N/PN/NI to 2.6: Was there potential for a substantial impact (on the result) of the failure to analyse participants in the group to which they were randomized?	NA	
	**Risk of bias judgement**	**Low**	Double-blind conduct; ITT GLMMs used.
**Bias due to missing outcome data**	3.1 Were data for this outcome available for all, or nearly all, participants randomized?	PN	Primary outcome available for 107/131 infants at 2 weeks (flow diagram Fig 1). Group-level usable traces noted in Table 2 footnote.
	3.2 If N/PN/NI to 3.1: Is there evidence that result was not biased by missing outcome data?	N	Authors note withdrawals (often admin/taste issues) and acknowledge potential attrition bias; no strong demonstration that missingness is ignorable.
	3.3 If N/PN to 3.2: Could missingness in the outcome depend on its true value?	NI	Likely unrelated (administration difficulties), but cannot exclude link with IH burden..
	3.4 If Y/PY/NI to 3.3: Is it likely that missingness in the outcome depended on its true value?	PN	No evidence it did; reasons seem logistical/tolerability. Still <100% follow-up at the primary timepoint.
	**Risk of bias judgement**	**Some concerns**	107/131 with primary oximetry at 2 weeks; withdrawals mainly admin/tolerability → bias cannot be excluded.
**Bias in measurement of the outcome**	4.1 Was the method of measuring the outcome inappropriate?	N	Standard overnight oximetry (Masimo), edited by a single investigator per protocol; ≥6 h edited data required.
	4.2 Could measurement or ascertainment of the outcome have differed between intervention groups?	N	Same devices/protocols; blinded editing/assessment.
	4.3 Were outcome assessors aware of the intervention received by study participants?	N	All personnel was blinded.
	4.4 If Y/PY/NI to 4.3: Could assessment of the outcome have been influenced by knowledge of intervention received?	NA	
	4.5 If Y/PY/NI to 4.4: Is it likely that assessment of the outcome was influenced by knowledge of intervention received?	NA	
	**Risk of bias judgement**	**Low**	Standardised, blinded oximetry processing; objective metric.
**Bias in selection of the reported result**	5.1 Were the data that produced this result analysed in accordance with a pre-specified analysis plan that was finalized before unblinded outcome data were available for analysis?	Y	Registered (ACTRN12618001745235) with published protocol; primary outcome/timepoint prespecified; analysis approach described a priori.

	5.2 ... multiple eligible outcome measurements (e.g. scales, definitions, time points) within the outcome domain?	N	Single primary definition (IH events/h at 2 weeks).
	5.3 ... multiple eligible analyses of the data?	PN	Dose vs placebo comparisons prespecified; no multiplicity adjustment for secondaries, but primary fixed.
	**Risk of bias judgement**	**Low**	Registered protocol; single prespecified primary
**Overall bias**	**Risk of bias judgement**	**Some concerns**	Reason: Strong randomisation, concealment, blinding, and objective measurement; primary limitation is incomplete primary-outcome data (107/131) with uncertain ignorability.

**Unique ID**	19	**Study ID**	Anggrainy et al (2024)	**Assessor**	
**Ref or Label**	Anggrainy et al (2024)	**Aim**	assignment to intervention (the 'intention-to-treat' effect)		
**Experimental**	Oral caffeine citrate 20 mg/kg load, then 5–10 mg/kg/day × 7 days	**Comparator**	Oral theophylline 5–8 mg/kg load, then 4–22 mg/kg every 6–8 h × 7 days	**Source**	Journal article(s)
**Outcome**	Daily frequency of apnea after treatment (episodes/24 h), averaged over the treatment period; episodes defined as >20 s or shorter with bradycardia <100 bpm and SpO_2 <85% (counted nightly)	**Results**	Mean daily apnea episodes after treatment: 3.16 ± 1.31 (caffeine) vs 2.28 ± 1.40 (theophylline); mean difference = +0.88 episodes/24 h (higher with caffeine), p = 0.031. (Baseline initial apnea frequency similar: 3.12 ± 0.72 vs 3.40 ± 0.91.)	**Weight**	1

Domain	Signalling question	Response	Comments
**Bias arising from the randomization process**	1.1 Was the allocation sequence random?	Y	"Randomized…using simple randomization" by the pharmacist at AOP diagnosis.
	1.2 Was the allocation sequence concealed until participants were enrolled and assigned to interventions?	NI	Randomized by pharmacist, but no explicit concealment method (e.g., sealed opaque envelopes/pharmacy-controlled packs) described.
	1.3 Did baseline differences between intervention groups suggest a problem with the randomization process?	N	Baseline characteristics (sex, GA, BW, mode of delivery, age at AOP, initial apnea frequency) were similar (Table 1).
	**Risk of bias judgement**	**Some concerns**	Simple randomization by pharmacist; concealment not described; baseline balanced.
**Bias due to deviations from intended interventions**	2.1.Were participants aware of their assigned intervention during the trial?	N	Neonates cannot be aware.
	2.2.Were carers and people delivering the interventions aware of participants' assigned intervention during the trial?	Y	Trial not blinded to treating staff; different oral dosing schedules.
	2.3. If Y/PY/NI to 2.1 or 2.2: Were there deviations from the intended intervention that arose because of the experimental context?	NI	No specific protocol-deviations reported; identical outcome-counting schedule used.
	2.4 If Y/PY to 2.3: Were these deviations likely to have affected the outcome?	NA	
	2.5. If Y/PY/NI to 2.4: Were these deviations from intended intervention balanced between groups?	NA	
	2.6 Was an appropriate analysis used to estimate the effect of assignment to intervention?	Y	All 25 vs 25 randomized infants were included for the primary outcome; between-group comparison by appropriate tests.
	2.7 If N/PN/NI to 2.6: Was there potential for a substantial impact (on the result) of the failure to analyse participants in the group to which they were randomized?	NA	
	**Risk of bias judgement**	**Some concerns**	Open-label to carers; all randomized analysed; no reported protocol-deviations affecting outcome.
**Bias due to missing**	3.1 Were data for this outcome available for all, or nearly all, participants randomized?	Y	Outcomes are reported for all 50 infants.

**outcome data**	3.2 If N/PN/NI to 3.1: Is there evidence that result was not biased by missing outcome data?	NA	
	3.3 If N/PN to 3.2: Could missingness in the outcome depend on its true value?	NA	
	3.4 If Y/PY/NI to 3.3: Is it likely that missingness in the outcome depended on its true value?	NA	
	**Risk of bias judgement**	**Low**	Primary outcome available for all 50.
**Bias in measurement of the outcome**	4.1 Was the method of measuring the outcome inappropriate?	N	Apnea episodes defined a priori and counted daily using monitor/clinical criteria.
	4.2 Could measurement or ascertainment of the outcome have differed between intervention groups?	N	Same NICU setting, same daily counting procedure for both arms.
	4.3 Were outcome assessors aware of the intervention received by study participants?	N	"The study treatment…was unknown to the investigator assessing the outcomes."
	4.4 If Y/PY/NI to 4.3: Could assessment of the outcome have been influenced by knowledge of intervention received?	NA	
	4.5 If Y/PY/NI to 4.4: Is it likely that assessment of the outcome was influenced by knowledge of intervention received?	NA	
	**Risk of bias judgement**	**Low**	Apnea episodes counted daily per definition; assessor blinded.
**Bias in selection of the reported result**	5.1 Were the data that produced this result analysed in accordance with a pre-specified analysis plan that was finalized before unblinded outcome data were available for analysis?	NI	No trial registration or SAP is reported.
	5.2 ... multiple eligible outcome measurements (e.g. scales, definitions, time points) within the outcome domain?	PY	Apnea was counted daily across 7 days; authors report a mean after treatment, leaving potential flexibility in summarisation.
	5.3 ... multiple eligible analyses of the data?	NI	They used standard (Mann–Whitney/ttest) comparisons; analytic alternatives not detailed.
	**Risk of bias judgement**	**Some concerns**	No registration/SAP; daily counts summarised as mean after treatment.
**Overall bias**	**Risk of bias judgement**	**Some concerns**	Unclear allocation concealment and unblinded caregivers (D1–D2), plus some flexibility in summarising daily counts (D5). Outcome

			measurement itself was blinded and complete (D3–D4).

Unique ID	20	Study ID	Bashar et al (2024)	Assessor	
Ref or Label	Bashar et al (2024)	Aim	assignment to intervention (the 'intention-to-treat' effect)		
Experimental	Aminophylline 5 mg/kg load then 2 mg/kg q8h	Comparator	Caffeine 20 mg/kg load then 5 mg/kg daily.	Source	Journal article(s)
Outcome	Need for CPAP during admission (yes/no). (A main outcome is not explicitly pre-specified; CPAP need is the key management endpoint reported with a significant between-group difference.)	Results	Caffeine 20 mg/kg load then 5 mg/kg daily.	Weight	1

Domain	Signalling question	Response	Comments
Bias arising from the randomization process	1.1 Was the allocation sequence random?	N	Allocation based on birth-date odd/even (predictable; not a truly random sequence).
	1.2 Was the allocation sequence concealed until participants were enrolled and assigned to interventions?	N	Birth-date rule makes allocation foreseeable to recruiters/clinicians.
	1.3 Did baseline differences between intervention groups suggest a problem with the randomization process?	Y	Birth weight higher in aminophylline (1.5 ± 0.4 kg) vs caffeine (1.3 ± 0.3 kg), p=0.019; 5-min Apgar lower in aminophylline (7.4 ± 1.8) vs caffeine (8.9 ± 0.9), p<0.001.
	**Risk of bias judgement**	**High**	Allocation by birth-date odd/even (predictable); baseline imbalances in weight and Apgar.
Bias due to deviations	2.1.Were participants aware of their assigned intervention during the trial?	N	Neonates cannot be aware.

**from intended interventions**	2.2.Were carers and people delivering the interventions aware of participants' assigned intervention during the trial?	Y	Open-label; different dosing schedules (q8h vs daily).
	2.3. If Y/PY/NI to 2.1 or 2.2: Were there deviations from the intended intervention that arose because of the experimental context?	PY	Knowledge of treatment could influence thresholds to start CPAP or ventilate.
	2.4 If Y/PY to 2.3: Were these deviations likely to have affected the outcome?	PY	CPAP/ventilator initiation is clinician-managed and plausibly affected by knowing the infant received caffeine vs aminophylline.
	2.5. If Y/PY/NI to 2.4: Were these deviations from intended intervention balanced between groups?	NI	The report does not document measures to ensure any expectation-driven decisions were balanced.
	2.6 Was an appropriate analysis used to estimate the effect of assignment to intervention?	Y	Outcomes are presented for all 55 infants by allocated group (no post-randomization exclusions reported).
	2.7 If N/PN/NI to 2.6: Was there potential for a substantial impact (on the result) of the failure to analyse participants in the group to which they were randomized?	NA	
	**Risk of bias judgement**	**Some concerns**	Open-label with clinician-managed outcome; all infants analysed by group; deviations could affect CPAP decisions; balance unknown.
**Bias due to missing outcome data**	3.1 Were data for this outcome available for all, or nearly all, participants randomized?	Y	Tables report CPAP need for the entire cohort (n=55).
	3.2 If N/PN/NI to 3.1: Is there evidence that result was not biased by missing outcome data?	NA	
	3.3 If N/PN to 3.2: Could missingness in the outcome depend on its true value?	NA	
	3.4 If Y/PY/NI to 3.3: Is it likely that missingness in the outcome depended on its true value?	NA	
	**Risk of bias judgement**	**Low**	CPAP outcome reported for all 55 neonates.
**Bias in measurement of the outcome**	4.1 Was the method of measuring the outcome inappropriate?	N	CPAP use is a standard clinical endpoint recorded in routine care.
	4.2 Could measurement or ascertainment of the outcome have differed between intervention groups?	PY	Same NICU, but open-label conduct may change clinicians' thresholds for starting CPAP.
	4.3 Were outcome assessors aware of the intervention received by study participants?	NA	

	4.4 If Y/PY/NI to 4.3: Could assessment of the outcome have been influenced by knowledge of intervention received?	NA	
	4.5 If Y/PY/NI to 4.4: Is it likely that assessment of the outcome was influenced by knowledge of intervention received?	NA	
	**Risk of bias judgement**	**Some concerns**	Standard clinical endpoint but unblinded assessors and management → Some concerns.
**Bias in selection of the reported result**	5.1 Were the data that produced this result analysed in accordance with a pre-specified analysis plan that was finalized before unblinded outcome data were available for analysis?	NI	No trial registration or SAP reported.
	5.2 ... multiple eligible outcome measurements (e.g. scales, definitions, time points) within the outcome domain?	PY	Several management outcomes reported (CPAP need/duration, ventilator need/duration, apnea, bradycardia).
	5.3 ... multiple eligible analyses of the data?	NI	Simple χ²/t/Mann–Whitney described; flexibility not detailed.
	**Risk of bias judgement**	**Some concerns**	No protocol/SAP; multiple management endpoints reported
**Overall bias**	**Risk of bias judgement**	**High**	Quasi-random predictable allocation with baseline imbalances (Domain 1 = High), open-label management of a clinician-driven outcome (Domains 2 & 4 = Some concerns), and no pre-registered SAP (Domain 5).

**Unique ID**	21	**Study ID**	Raza et al(2024)	**Assessor**	
**Ref or Label**	Raza et al(2024)	**Aim**	assignment to intervention (the 'intention-to-treat' effect)		
**Experimental**	Caffeine citrate IV — loading 20 mg/kg (caffeine base), maintenance 5 mg/kg q24h	**Comparator**	Aminophylline IV — loading 5 mg/kg, maintenance 1.5 mg/kg q12h.	**Source**	Journal article(s)

Outcome	no reappearance of apnea after the initial episode over a ≥3-day observation window (in-hospital).	Results	Caffeine 87% vs Aminophylline 63%.	Weight	1

Domain	Signalling question	Response	Comments
**Bias arising from the randomization process**	1.1 Was the allocation sequence random?	NI	The report does not describe a random sequence or method (e.g., computer-generated, tables).
	1.2 Was the allocation sequence concealed until participants were enrolled and assigned to interventions?	NI	No details on concealment (e.g., pharmacy codes, sealed envelopes).
	1.3 Did baseline differences between intervention groups suggest a problem with the randomization process?	PN	Baseline characteristics appear broadly similar.
	**Risk of bias judgement**	**High**	Randomization not demonstrated; information insufficient to verify concealment.
**Bias due to deviations from intended interventions**	2.1.Were participants aware of their assigned intervention during the trial?	Y	
	2.2.Were carers and people delivering the interventions aware of participants' assigned intervention during the trial?	Y	
	2.3. If Y/PY/NI to 2.1 or 2.2: Were there deviations from the intended intervention that arose because of the experimental context?	PN	No protocol deviations reported; however, open-label care could influence co-interventions.
	2.4 If Y/PY to 2.3: Were these deviations likely to have affected the outcome?	NA	
	2.5. If Y/PY/NI to 2.4: Were these deviations from intended intervention balanced between groups?	NA	
	2.6 Was an appropriate analysis used to estimate the effect of assignment to intervention?	N	No intention-to-treat framework; unadjusted comparisons only; no protection against performance bias.
	2.7 If N/PN/NI to 2.6: Was there potential for a substantial impact (on the result) of the failure to analyse participants in the group to which they were randomized?		
	**Risk of bias judgement**	**High**	Open-label; analysis not appropriate for effect of assignment.
**Bias due to missing**	3.1 Were data for this outcome available for all, or nearly all, participants randomized?	Y	In-hospital follow-up complete; no meaningful loss to follow-up reported.

outcome data	3.2 If N/PN/NI to 3.1: Is there evidence that result was not biased by missing outcome data?	NA	
	3.3 If N/PN to 3.2: Could missingness in the outcome depend on its true value?	NA	
	3.4 If Y/PY/NI to 3.3: Is it likely that missingness in the outcome depended on its true value?	NA	
	**Risk of bias judgement**	**Low**	In-hospital follow-up complete; no meaningful loss to follow-up reported.
**Bias in measurement of the outcome**	4.1 Was the method of measuring the outcome inappropriate?	N	Clinical monitoring for apnea/oxygen/ventilation is standard in NICU settings.
	4.2 Could measurement or ascertainment of the outcome have differed between intervention groups?	PY	Open-label design could influence thresholds for oxygen/ventilation initiation.
	4.3 Were outcome assessors aware of the intervention received by study participants?	NA	
	4.4 If Y/PY/NI to 4.3: Could assessment of the outcome have been influenced by knowledge of intervention received?	NA	
	4.5 If Y/PY/NI to 4.4: Is it likely that assessment of the outcome was influenced by knowledge of intervention received?	NA	
	**Risk of bias judgement**	**Some concerns**	Potential detection/performance bias for clinician-driven outcomes.
**Bias in selection of the reported result**	5.1 Were the data that produced this result analysed in accordance with a pre-specified analysis plan that was finalized before unblinded outcome data were available for analysis?	NI	No protocol/preregistration available; analytical approach not pre-specified.
	5.2 ... multiple eligible outcome measurements (e.g. scales, definitions, time points) within the outcome domain?	NI	Insufficient information to determine multiplicity handling.
	5.3 ... multiple eligible analyses of the data?	NI	
	**Risk of bias judgement**	**Some concerns**	Insufficient information to determine multiplicity handling.
**Overall bias**	**Risk of bias judgement**	**High**	Randomization not demonstrated (D1 High), open-label with non-ITT/unadjusted analysis (D2 High), plus some concerns in measurement and reporting.

**Unique ID**	22	**Study ID**	Carlo et al (2025)	**Assessor**	
**Ref or Label**	Carlo et al (2025)	**Aim**	assignment to intervention (the 'intention-to-treat' effect)		
**Experimental**	Oral caffeine citrate 10 mg/kg/day (5 mg/kg base), continued through hospitalization and 28 days post-discharge	**Comparator**	Placebo (same excipients/volume); pharmacy-controlled; all staff, families, and assessors masked	**Source**	
**Outcome**	Days of hospitalization from randomization to discharge (censored at transfer, death, or 48 w PMA).	**Results**	Days to discharge (median [IQR]): 18.0 (10–30) caffeine vs 16.5 (10–27) placebo; adjusted median difference 0 days (95% CI −1.7 to +1.7), P > .99. (No reduction with extended caffeine.)	**Weight**	1

Domain	Signalling question	Response	Comments
**Bias arising from the randomization process**	1.1 Was the allocation sequence random?	Y	Internet central randomization; stratified by site and GA; varying block size.
	1.2 Was the allocation sequence concealed until participants were enrolled and assigned to interventions?	Y	Pharmacy-controlled identical study drug; all staff/families/assessors masked.
	1.3 Did baseline differences between intervention groups suggest a problem with the randomization process?	N	Maternal and neonatal characteristics similar between groups (Table 1).
	**Risk of bias judgement**	**Low**	Central internet randomization; concealed; balanced baseline.

**Bias due to deviations from intended interventions**	2.1.Were participants aware of their assigned intervention during the trial?	N	Neonates cannot be aware.<br><br>All staff and families were blinded.
	2.2.Were carers and people delivering the interventions aware of participants' assigned intervention during the trial?	N	
	2.3. If Y/PY/NI to 2.1 or 2.2: Were there deviations from the intended intervention that arose because of the experimental context?	NA	
	2.4 If Y/PY to 2.3: Were these deviations likely to have affected the outcome?	NA	
	2.5. If Y/PY/NI to 2.4: Were these deviations from intended intervention balanced between groups?	NA	
	2.6 Was an appropriate analysis used to estimate the effect of assignment to intervention?	Y	ITT; median regression with censoring, adjusted for site & GA (pre-specified).
	2.7 If N/PN/NI to 2.6: Was there potential for a substantial impact (on the result) of the failure to analyse participants in the group to which they were randomized?	NA	
	**Risk of bias judgement**	**Low**	Double-blind; prespecified ITT median-regression; individualized management under masking.
**Bias due to missing outcome data**	3.1 Were data for this outcome available for all, or nearly all, participants randomized?	Y	Primary outcome analyzed for all randomized (416 vs 411); censoring rules pre-specified.
	3.2 If N/PN/NI to 3.1: Is there evidence that result was not biased by missing outcome data?	NA	
	3.3 If N/PN to 3.2: Could missingness in the outcome depend on its true value?	NA	
	3.4 If Y/PY/NI to 3.3: Is it likely that missingness in the outcome depended on its true value?	NA	
	**Risk of bias judgement**	**Low**	Primary outcome available for all randomized; censoring per protocol.
**Bias in measurement of the outcome**	4.1 Was the method of measuring the outcome inappropriate?	N	Days to discharge from hospital records; standard, objective.
	4.2 Could measurement or ascertainment of the outcome have differed between intervention groups?	N	Same discharge criteria; blinding minimizes differential application.

	4.3 Were outcome assessors aware of the intervention received by study participants?	N	Masked clinicians/outcome assessors.
	4.4 If Y/PY/NI to 4.3: Could assessment of the outcome have been influenced by knowledge of intervention received?	NA	
	4.5 If Y/PY/NI to 4.4: Is it likely that assessment of the outcome was influenced by knowledge of intervention received?	NA	
	**Risk of bias judgement**	**Low**	Objective administrative outcome; masked assessors.
**Bias in selection of the reported result**	5.1 Were the data that produced this result analysed in accordance with a pre-specified analysis plan that was finalized before unblinded outcome data were available for analysis?	Y	Protocol & SAP available (Supplements); primary & analyses prespecified.
	5.2 ... multiple eligible outcome measurements (e.g. scales, definitions, time points) within the outcome domain?	N	Single primary metric: days from randomization to discharge.
	5.3 ... multiple eligible analyses of the data?	PN	Primary analysis model prespecified; sensitivity survival models explored but not primary.
	**Risk of bias judgement**	**Low**	Protocol & SAP published; single primary outcome; prespecified model.
**Overall bias**	**Risk of bias judgement**	**Low**	Strong randomization & concealment, double-blinding, ITT analysis, objective primary outcome with complete ascertainment.

Supplementary table S3. PRISMA 2020 for abstracts checklist.

Section and Topic	Item #	Checklist item	Reported (Yes/No)
**TITLE**			
Title	1	Identify the report as a systematic review.	Yes
**BACKGROUND**			
Objectives	2	Provide an explicit statement of the main objective(s) or question(s) the review addresses.	Yes
**METHODS**			
Eligibility criteria	3	Specify the inclusion and exclusion criteria for the review.	Yes

Section and Topic	Item #	Checklist item	Reported (Yes/No)
Information sources	4	Specify the information sources (e.g. databases, registers) used to identify studies and the date when each was last searched.	Yes
Risk of bias	5	Specify the methods used to assess risk of bias in the included studies.	Yes
Synthesis of results	6	Specify the methods used to present and synthesise results.	Yes
**RESULTS**			
Included studies	7	Give the total number of included studies and participants and summarise relevant characteristics of studies.	Yes
Synthesis of results	8	Present results for main outcomes, preferably indicating the number of included studies and participants for each. If meta-analysis was done, report the summary estimate and confidence/credible interval. If comparing groups, indicate the direction of the effect (i.e. which group is favoured).	Yes
**DISCUSSION**			
Limitations of evidence	9	Provide a brief summary of the limitations of the evidence included in the review (e.g. study risk of bias, inconsistency and imprecision).	Yes
Interpretation	10	Provide a general interpretation of the results and important implications.	Yes
**OTHER**			
Funding	11	Specify the primary source of funding for the review.	No
Registration	12	Provide the register name and registration number.	No

Supplementary table S4. PRISMA 2020 Checklist.

Section and Topic	Item #	Checklist item	Location where item is reported
**TITLE**			
Title	1	Identify the report as a systematic review.	1
**ABSTRACT**			
Abstract	2	See the PRISMA 2020 for Abstracts checklist.	2
**INTRODUCTION**			
Rationale	3	Describe the rationale for the review in the context of existing knowledge.	9
Objectives	4	Provide an explicit statement of the objective(s) or question(s) the review addresses.	9
**METHODS**			
Eligibility criteria	5	Specify the inclusion and exclusion criteria for the review and how studies were grouped for the syntheses.	10
Information sources	6	Specify all databases, registers, websites, organisations, reference lists and other sources searched or consulted to identify studies. Specify the date when each source was last searched or consulted.	10
Search strategy	7	Present the full search strategies for all databases, registers and websites, including any filters and limits used.	10, Suppl. Table S1.
Selection process	8	Specify the methods used to decide whether a study met the inclusion criteria of the review, including how many reviewers screened each record and each report retrieved, whether they worked independently, and if applicable, details of automation tools used in the process.	11
Data collection process	9	Specify the methods used to collect data from reports, including how many reviewers collected data from each report, whether they worked independently, any processes for obtaining or confirming data from study investigators, and if applicable, details of automation tools used in the process.	11
Data items	10a	List and define all outcomes for which data were sought. Specify whether all results that were compatible with each outcome domain in each study were sought (e.g. for all measures, time points, analyses), and if not, the methods used to decide which results to collect.	15
	10b	List and define all other variables for which data were sought (e.g. participant and intervention characteristics, funding sources). Describe any assumptions made about any missing or unclear information.	12
Study risk of bias assessment	11	Specify the methods used to assess risk of bias in the included studies, including details of the tool(s) used, how many reviewers assessed each study and whether they worked independently, and if applicable, details of automation tools used in the process.	14, Figure 3, Supplementary Table S2
Effect measures	12	Specify for each outcome the effect measure(s) (e.g. risk ratio, mean difference) used in the synthesis or presentation of results.	16
Synthesis methods	13a	Describe the processes used to decide which studies were eligible for each synthesis (e.g. tabulating the study intervention characteristics and comparing against the planned groups for each synthesis (item #5)).	11
	13b	Describe any methods required to prepare the data for presentation or synthesis, such as handling of missing summary statistics, or data conversions.	12
	13c	Describe any methods used to tabulate or visually display results of individual studies and syntheses.	23
	13d	Describe any methods used to synthesize results and provide a rationale for the choice(s). If meta-analysis was performed, describe the model(s), method(s) to identify the presence and extent of statistical heterogeneity, and software package(s) used.	15

Section and Topic	Item #	Checklist item	Location where item is reported
	13e	Describe any methods used to explore possible causes of heterogeneity among study results (e.g. subgroup analysis, meta-regression).	18
	13f	Describe any sensitivity analyses conducted to assess robustness of the synthesized results.	18
Reporting bias assessment	14	Describe any methods used to assess risk of bias due to missing results in a synthesis (arising from reporting biases).	14
Certainty assessment	15	Describe any methods used to assess certainty (or confidence) in the body of evidence for an outcome.	17
**RESULTS**			
Study selection	16a	Describe the results of the search and selection process, from the number of records identified in the search to the number of studies included in the review, ideally using a flow diagram.	14
	16b	Cite studies that might appear to meet the inclusion criteria, but which were excluded, and explain why they were excluded.	12
Study characteristics	17	Cite each included study and present its characteristics.	24-29, Table 2
Risk of bias in studies	18	Present assessments of risk of bias for each included study.	14-15, Figure 3, Supplementary Table S2
Results of individual studies	19	For all outcomes, present, for each study: (a) summary statistics for each group (where appropriate) and (b) an effect estimate and its precision (e.g. confidence/credible interval), ideally using structured tables or plots.	24-29, Table 2
Results of syntheses	20a	For each synthesis, briefly summarise the characteristics and risk of bias among contributing studies.	19, 22, 32
	20b	Present results of all statistical syntheses conducted. If meta-analysis was done, present for each the summary estimate and its precision (e.g. confidence/credible interval) and measures of statistical heterogeneity. If comparing groups, describe the direction of the effect.	19-23
	20c	Present results of all investigations of possible causes of heterogeneity among study results.	18
	20d	Present results of all sensitivity analyses conducted to assess the robustness of the synthesized results.	19
Reporting biases	21	Present assessments of risk of bias due to missing results (arising from reporting biases) for each synthesis assessed.	14-15, Figure 3, Supplementary Table S2
Certainty of evidence	22	Present assessments of certainty (or confidence) in the body of evidence for each outcome assessed.	17
**DISCUSSION**			
Discussion	23a	Provide a general interpretation of the results in the context of other evidence.	34
	23b	Discuss any limitations of the evidence included in the review.	36
	23c	Discuss any limitations of the review processes used.	36

Section and Topic	Item #	Checklist item	Location where item is reported
	23d	Discuss implications of the results for practice, policy, and future research.	37
**OTHER INFORMATION**			
Registration and protocol	24a	Provide registration information for the review, including register name and registration number, or state that the review was not registered.	N/A (10)
	24b	Indicate where the review protocol can be accessed, or state that a protocol was not prepared.	N/A (10)
	24c	Describe and explain any amendments to information provided at registration or in the protocol.	N/A (10)
Support	25	Describe sources of financial or non-financial support for the review, and the role of the funders or sponsors in the review.	39
Competing interests	26	Declare any competing interests of review authors.	39
Availability of data, code and other materials	27	Report which of the following are publicly available and where they can be found: template data collection forms; data extracted from included studies; data used for all analyses; analytic code; any other materials used in the review.	N/A
